# Natural 6-hydroxy-chromanols and -chromenols: structural diversity, biosynthetic pathways and health implications†

**DOI:** 10.1039/c7ra11819h

**Published:** 2018-01-26

**Authors:** Marc Birringer, Karsten Siems, Alexander Maxones, Jan Frank, Stefan Lorkowski

**Affiliations:** Department of Nutritional, Food and Consumer Sciences, Fulda University of Applied Sciences Leipziger Straße 123 36037 Fulda Germany marc.birringer@oe.hs-fulda.de; AnalytiCon Discovery GmbH Hermannswerder Haus 17 14473 Potsdam Germany k.siems@ac-discovery.com; Institute of Biological Chemistry and Nutrition, University of Hohenheim Garbenstr. 28 70599 Stuttgart Germany jan.frank@nutres.de; Institute of Nutrition, Friedrich Schiller University Jena Dornburger Str. 25 07743 Jena Germany stefan.lorkowski@uni-jena.de; Competence Cluster for Nutrition and Cardiovascular Health (nutriCARD), Halle-Jena-Leipzig Germany

## Abstract

We present the first comprehensive and systematic review on the structurally diverse toco-chromanols and -chromenols found in photosynthetic organisms, including marine organisms, and as metabolic intermediates in animals. The focus of this work is on the structural diversity of chromanols and chromenols that result from various side chain modifications. We describe more than 230 structures that derive from a 6-hydroxy-chromanol- and 6-hydroxy-chromenol core, respectively, and comprise di-, sesqui-, mono- and hemiterpenes. We assort the compounds into a structure–activity relationship with special emphasis on anti-inflammatory and anti-carcinogenic activities of the congeners. This review covers the literature published from 1970 to 2017.

## Abstract 2

We present the first comprehensive and systematic review on the structurally diverse toco-chromanols and -chromenols found in photosynthetic organisms, including marine organisms, and as metabolic intermediates in animals.

## Graphical abstract

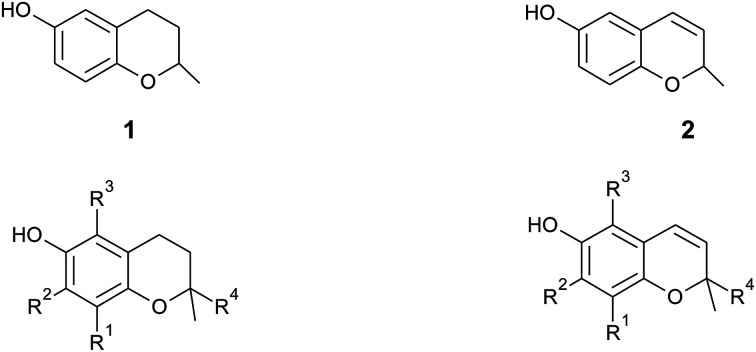

## 1. Introduction

In 1922, Bishop and Evans discovered α-tocopherol as an essential lipid-soluble factor that promotes the gestation of rat fetuses.^1^ Since then, numerous structurally related 6-hydroxy-chromanols and -chromenols have been discovered. Tocochromanols of the vitamin E class represent the most widely distributed and predominant chromanols in nature. However, only photosynthetic organisms, such as plants, algae, and cyanobacteria as well as fungi, corals, sponges and tunicates, are able to perform the biosynthetic steps leading to a chromanol ring system. However, mammals, including humans, rely on these resources (esp. plant oils), since vitamin E is essential for a wide range of higher organisms.^2^

The term vitamin E is traditionally used for the eight structurally related vitamers α-, β-, γ-, δ-tocopherol, and α-, β-, γ-, δ-tocotrienol, with α-tocopherol being the compound with the highest vitamin activity.^3^

Tocochromanols belong to the family of prenylquinones that also include plastochromanol-8, phylloquinones (vitamin K), and ubiquinones (coenzyme Q_10_). Due to its unique 6-hydroxy-chromanol structure, the vitamin E forms may act as antioxidants that prevent lipid peroxidation in cellular membranes and quench harmful reactive oxygen species (ROS) in plants and animals (including humans). The proton of the 6-hydroxy group can quench a reactive radical, in turn leading to a tocopheryl radical that, depending on the substitution pattern of the ring system, remains stable, with a half-life of several seconds, and can be subsequently recycled by vitamin C. The review does not aim to discuss the complex antioxidant and redox chemistry of tocopherols forming corresponding radicals, quinones, dimers or polymers. These issues have already been discussed in several excellent reviews.^4,5^ Further, biosynthesis, bioactivity and chemical properties of tocopherols and tocotrienols are summarized in several outstanding reviews,^6^ and will be discussed here only briefly. This work focuses on the structural diversity of chromanols due to side chain modifications and attempts to merge structural aspects with biological activity.

**Fig. 1:**
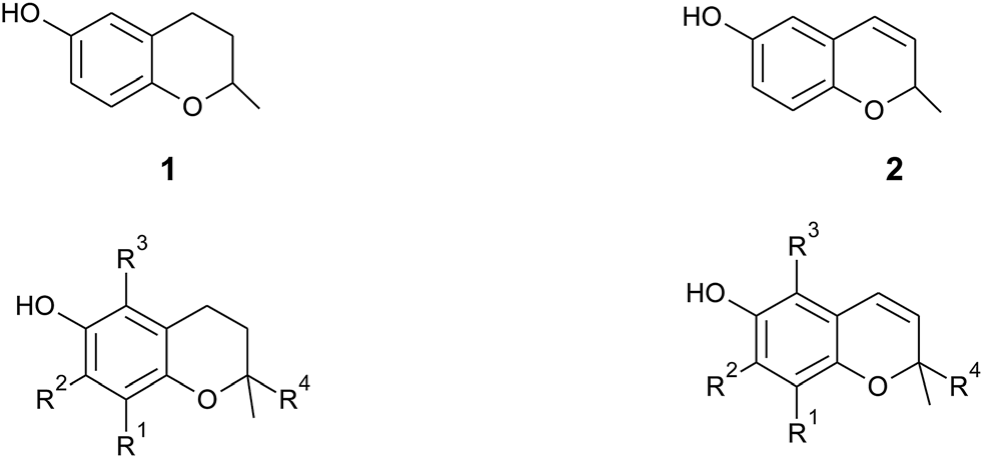
Core structures 2-methyl-3,4-dihydro-2*H*-chromen-6-ol (1) and 2-methyl-2*H*-chromen-6-ol (2) with substitution patterns of the chromanol and chromenol ring systems.

In general, 6-hydroxy-chromanols derive from the parent structure 2-methyl-3,4-dihydro-2*H*-chromen-6-ol (1) and 6-hydroxy-chromenols derive from 2-methyl-2*H*-chromen-6-ol (2) that comprise a class of bicyclic heterocycles formed by cyclisation of substituted 1,4-benzoquinones (Fig. 1).

Besides the methylation pattern (R_1_–R_3_) of the chromanol ring system, side chain modifications (at R_4_) show the highest structural variability. In particular tocotrienols are prone to (partial) reduction of the double bonds or oxidation of the methyl groups by cytochrome P_450_-dependent hydroxylases and oxidases, which ultimately results in the formation of oxidation products, such as alcohols, ketones, aldehydes, carboxylic acids, and truncations of the side chain. Furthermore, intramolecular cyclisation and/or rearrangements of the isoprene units can build up mono-, bi-, and tri-cyclic ring systems. These modifications are well known for compounds in marine organisms, especially in brown algae and sponges (see below), but have been found also in higher plant species. Along with side chain modifications, increased bioactivity is observed for many of these structures *in vitro* and *in vivo*.

The following chapters describe these compounds, sorted by the length of the carbon skeleton, following the order (mero)-diterpenes, -sesquiterpenes, -monoterpenes and -hemiterpenes.

## 2. Methods

Chromanols and chromenols presented here were selected by a chemical substructure search of 1 and 2, respectively, within several databases. We received 307 matches from the Dictionary of Natural Products and 128 matches from the Dictionary of Marine Natural Products (both at Chemnet BASE). We included a PubChem substructure search and PubMed keyword searches for “tocochromanol*”, “tocochromenol*” and “mero(di)terpenoid*”. Patents were searched by chemical names at the website of the European Patent Office.^7^ Finally, we performed a reference-related snowball sampling and deleted all doublets. All identified meroterpenoids were sorted by the length of their carbon-skeleton and number of prenyl units, respectively. Numbering of the carbon skeleton of metabolites was conducted in analogy to IUPAC rules, however for better clearness, side chain numbers were primed (*e.g.* 13′, see Fig. 3). Metabolites were further classified by their occurrence in the above-mentioned species (including animal metabolism) and not by structural matching. Within each species, metabolites were sorted by functionalization of the side chain (*e.g.* saturated, unsaturated and oxidized). Physio-chemical properties were predicted by Molinspiration WebME editor version 1.16 (http://www.molinspiration.com).

With respect of the extent of the review, we excluded corresponding oxidized 1,4-benzoquinones or dimeric (and polymeric) structures that derive from natural or chemical oxidation processes that may occur during work-up procedures.

Many natural products with phenolic hydroxy groups, *e.g.* flavonoids, cumarins, caffeic acids, anthraquinones, or xanthones, bear a prenyl or to a minor extent geranyl or farnesyl residues in ortho position to the phenol. In some cases, this phenol forms a six-membered chromene ring by addition to the double bond of the prenyl (geranyl, farnesyl) residue. These mainly plant-derived compounds are not related to tocopherol biosynthesis and usually do not have the substructure of the 6-OH-chromanol. These chromanols are also not covered by this review.

## 3. Meroditerpenes

### 3.1 Plants

In the last decades, hundreds of publications referring to tocopherols and tocotrienols have been published, covering chemical, physical and biological properties of vitamin E as well as analytical procedures to detect the vitamers from biological origin.

**Fig. 2:**
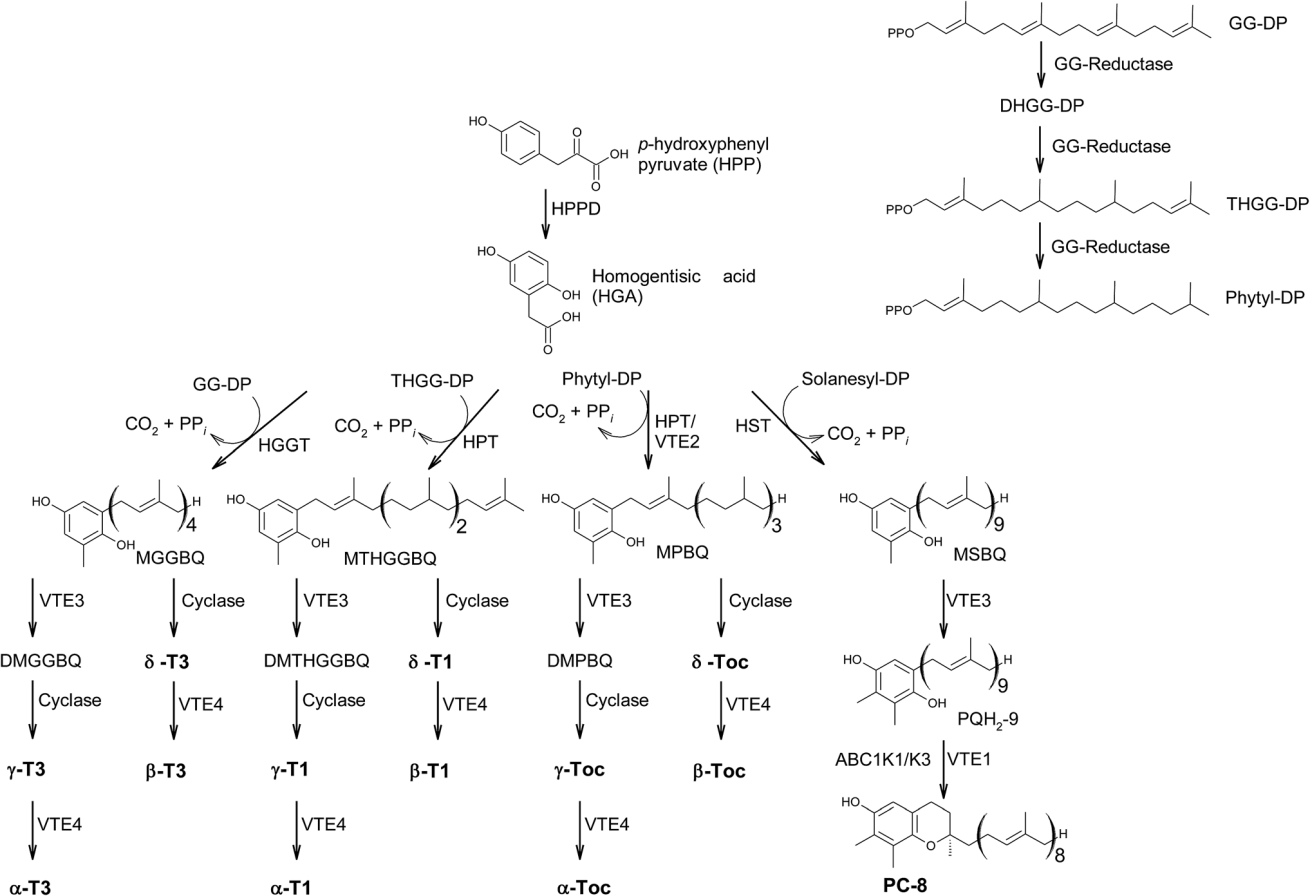
Biosynthetic pathway towards the formation of chromanols within photosynthetic organisms. Abr.: HPP: *p*-hydroxyphenylpyruvate; HGA: homogentisic acid; HPPD hydroxyphenylpyruvate dioxygenase; DOXP: 1-deoxy-d-xylulose-5-phosphate; GG-reductase: geranylgeraniol reductase; DHGG-DP: dihydro-geranylgeraniol diphosphate; THGG-DP: tetrahydro-geranylgeraniol diphosphate; MGGBQ: methyl-geranygeraniol benzoquinol; MTHGGBQ: methyl-tetrahydro-geranylgeraniol benzoquinol; MPBQ: methyl-phytyl benzoquinol; MSBQ: methyl-solanesyl benzoquinol.

**Fig. 3:**
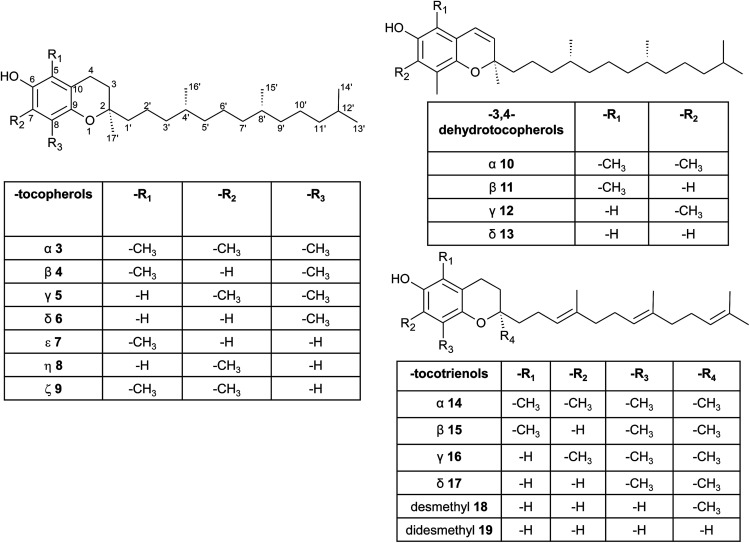
Structures and substitution patterns of tocopherols (3 to 9), dehydrotocopherols (10 to 13) and tocotrienols (14 to 19).

The main sources of the fat-soluble vitamin E are plant oils. To understand the structural variability of tocochromanols in plants and other photosynthetic organisms, a brief introduction into their biosynthesis is presented. The biosynthesis of tocochromanols was primarily investigated in the leaves of green plants, however all photosynthetic organisms as well as apicomplexa parasites such as *Plasmodium falciparum*^8^ are capable of the necessary biosynthetic steps. The biosynthetic pathways of tocotrienols, tocomonoenols, tocopherols and plastochromanol-8 are depicted in Fig. 2 and consist of five main steps. First, the transformation of *p*-hydroxyphenylpyruvate (HPP) to homogentisic acid (HGA), which is catalyzed by hydroxyphenylpyruvate dioxygenase (HPPD). Second, the synthesis of the isoprenoid side chain that originates from the 1-deoxy-d-xylulose-5-phosphate (DOXP) pathway in plastids. Here, geranylgeraniol reductase (GG-reductase) determines the degree of side chain saturation that leads to dihydro-geranylgeraniol diphosphate (DHGG-DP), tetrahydro-geranylgeraniol diphosphate (THGG-DP) and phytyl diphosphate, respectively. The reduction of the double bonds between C-3′–C-4′ and C-7′–C-8′ results in two *R*-configurated stereogenic centers at C-4′ and C-8′ of the later tocopherols (Fig. 3).

In addition, solanesyl diphosphate, containing nine isoprene units, is formed by solanesyl diphosphate synthase.^9^ The above-mentioned diphosphates serve as substrates for transferases, which catalyze the alkylation of HGA, leading to benzoquinol derivatives, such as methyl-geranygeraniol benzoquinol (MGGBQ), methyl-tetrahydro-geranylgeraniol benzoquinol (MTHGGBQ), methyl-phytyl benzoquinol (MPBQ), and methyl-solanesyl benzoquinol (MSBQ), respectively (step 3).

The methylation pattern of tocochromanols depends on the next steps (step 4 and 5) of the biosynthesis. δ- and β-tocochromanols are formed by immediate cyclization, followed by S-adenosyl methionine-dependent methylation of the chromanol ring, whereas γ-tocochromanols are build by methylation followed by cyclization. Finally, α-tocochromanols results from methylation of γ-tocochromanols. The cyclization of the prenylated quinones to chromanols by tocopherol cyclase occurs within plastoglobules. The latter biosynthetic step yields *R*-configuration at C-2 atom and thus seems to be unique for plant species. For in-depth details of the biosynthetic pathways, the reader is referred to previously published excellent reviews.^10,11^

According to the methylation pattern of the 6-hydroxy-chromanol ring system, tocopherols are divided into the most prominent vitamers α(5,7,8-trimethyl)-tocopherol (3), β(5,8-dimethyl)-tocopherol (4), γ(7,8-dimethyl)-tocopherol (5) and δ(8-methyl)-tocopherol (6), respectively (Fig. 3). The tocopherols are ubiquitously found in most plant oils, whereas tocotrienols occur only in non-photosynthetic organs of higher plants, mainly eudicots and monocots.^11,12^

Alternative methylations of the chromanol ring lead to ε-tocopherol (5-methyltocol) (7), η-tocopherol (7-methyltocol) (8) and ζ-tocopherol (5,7-dimethyltocol) (9), which are found in trace amounts in rice bran.^13^ The latter congeners have not been described in recent literature and therefore their existence seems to be questionable and may have been the result of analytical artifacts.

In the past, all tocopherols and tocotrienols were studied in gestation-fetal resorption assays, with *RRR*-α-tocopherol (3) being the most potent vitamer.^14^

Although described in textbooks, primary literature on tocopherylesters at C-6 is scarce.^15,16^ Acylesters of saturated fatty acids (C12:0, C14:0, C16:0 and C18:0) and tocopherols were found in *Nuphar luteum* and *Nymphea alba*^15^ and in the pulp of yellow bell pepper (*Capsicum annuum*).^16^ In the last decade, the occurrence, angiogenic and vasculogenic properties of α-tocopheryl-phosphate were intensively studied by Azzi *et al.*^17,18^ This molecule was found in food but only in low amounts.^18^

Dehydrotocopherols derive from the biochemical elimination between C-3 and C-4 of the chromanol ring and were first isolated as α-, β- and γ-dehydrotocopherols (10, 11, 12) from wheat germ oil^19^ and from various *Stemona* species, such as Korean Stemonae Radix (Fig. 3) (*Stemona tuberosa*).^20,21^ γ-Dehydrotocopherol shows proliferative effects on mouse NIH 3T3 fibroblasts and a potential use as wound healing agent has been suggested.^21^

As mentioned above, α-, β-, γ- and δ-tocotrienols (14, 15, 16, 17) usually occur as trace vitamers, however, several plant tissues and oils accumulate higher amounts of tocotrienols. For example, α-tocotrienol was found in barley (76 mg/100 g), γ- and δ-tocotrienol in palm oil (36 mg and 8 mg/100 g, respectively). The distribution of tocotrienols in plant has been reviewed in detail elsewhere.^6,12,22^

In contrast to tocopherols, tocotrienols exhibit higher bioactivity in vertebrates. Ashan *et al.* recently reviewed the bioactivity of tocotrienols, which may act as anti-cancer, anti-diabetic, anti-inflammatory, antioxidant, immune-stimulatory, cardio-, neuro-, hepato- and nephro-protective molecules.^23^

**Fig. 4:**
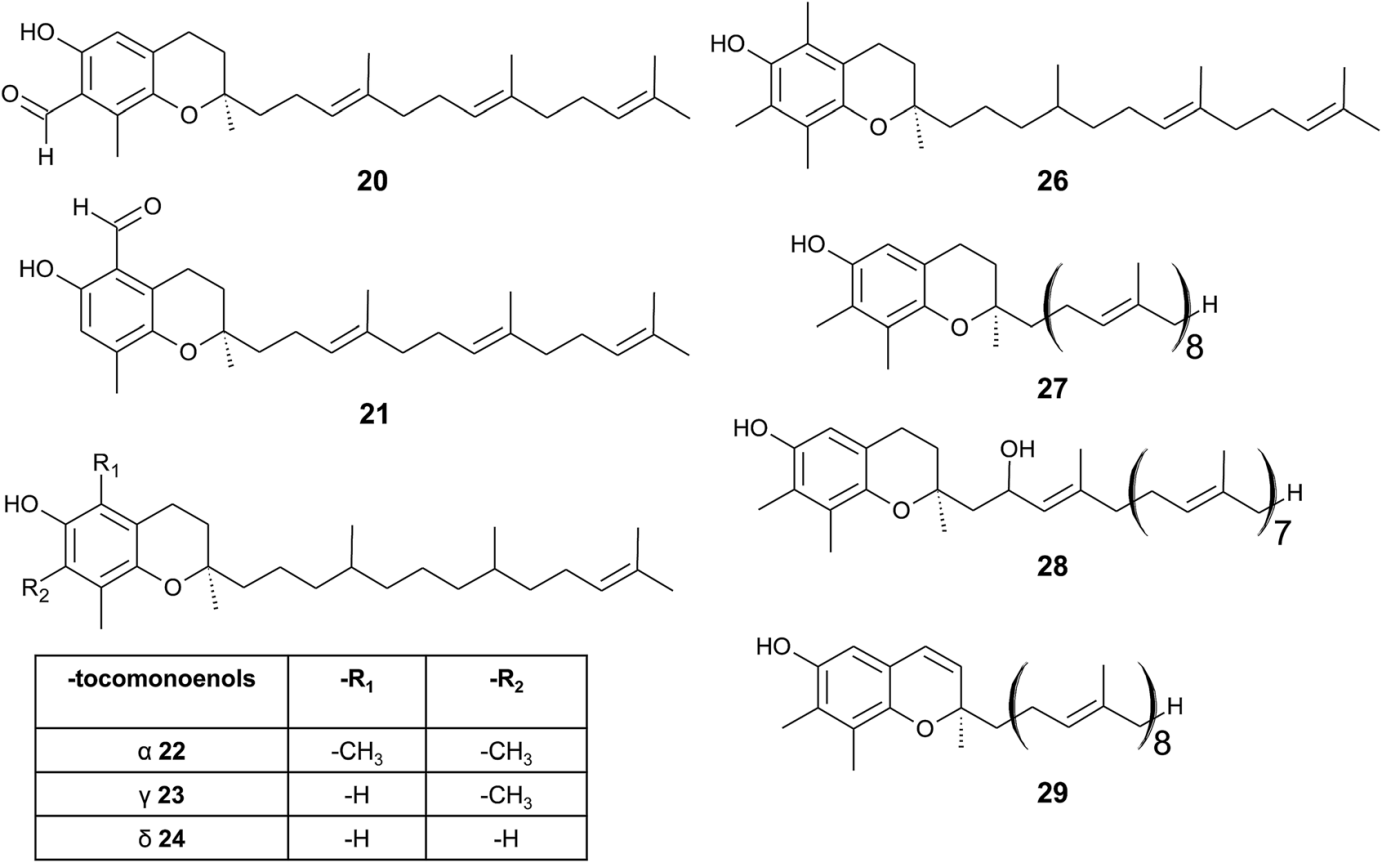
Structures and substitution patterns of formyl derivatives (20) and (21), tocomonoenols (22 to 24), tocodienol (26) and plastochromanols (27 to 29).

Alternation in the methylation pattern has been also described for tocotrienols. Qureshi *et al.* found desmethyltocotrienol (3,4-dihydro-2-methyl-2-(4,8,12-trimethyltrideca-3′(*E*),7′(*E*),11′-trienyl)-2*H*-1-benzopyran-6-ol) (18) and didesmethyltocotrienol (3,4-dihydro-2-(4,8,12-trimethyltrideca-3′(*E*),7′(*E*),11′-trienyl)-2*H*-1-benzopyran-6-ol) (19) in rice bran^24^ (Fig. 3). Most interestingly, the latter compounds show cholesterol lowering activity in chicken, most likely by inhibition of 3-hydroxy-3-methylglutaryl-CoA reductase, which catalayzes the rate-limiting step of the cholesterol biosynthesis pathway. The compounds reduced total serum cholesterol by 26% and 31% relative to a control diet and reduced LDL cholesterol by 41% and 48%, respectively. Similar to tocotrienols, both compounds suppress proliferation B16 melanoma cells^24,25^ (Table 3). Interestingly, oxidation of the aromatic methyl groups are rare in nature. Two unusual formyl-derivatives (at C-5 (20) and C-7 (21), respectively) of δ-tocotrienol have been isolated in trace amounts from *Garcinia virgata* (Fig. 4).^26^

As a result of a partial reduction of the prenyl side chain by geranylgeraniol reductase during the synthesis of tocopherols, tocodienols and tocomonoenols were found in several plant species (Fig. 4).^11^ α-Tocomonoenol (22) was isolated in palm seed and pumpkin seed oils,^27,28^ γ-tocomonoenol (23) in pumpkin seed oil as well as green leaves and etiolated beans of *Kalanchoe daigremontiana* and *Phaseolus coccineus*,^11,28^ and δ-tocomonoenol (24) was found in kiwi fruits (*Actinidia chinensis*).^29^ Of note, marine-derived α-tocomonoenol (25) (MDT) is a structural isomer of the above mentioned α-tocomonoenol with a terminal double bond at C-13′ (see section on Phytoplankton (3.3.2)).

α-Tocodienol (26) has recently been discovered as a trace compound (0.2% of the total vitamin E content) in palm oil.^30^ Interestingly to note is a recent publication by Hammann *et al.*, who have tentatively identified 170 unsaturated tocochromanol compounds in palm oil by GC-MS, which were most likely produced (in trace amounts) by the thermal oil refining process and are thus unlikely genuine natural products.^31^

Beside tocopherols and tocotrienols, some plant species produce plastochromanol-8 (27), a γ-tocochromanol with eight isoprenoid units in the side chain. The biosynthesis of the polyterpene follows that of tocotrienols except of the use of solanesyl-diphosphate synthase to form the elongated side chain of plastochromanol-8 (Fig. 4).^32^ Plastochromanol-8 was first discovered in leaves of the rubber tree (*Hevea brasiliensis*) and since then in many higher plants, where it acts as a fat-soluble antioxidant.^32–34^ Nutritional sources, such as rapeseed and linseed oil, accumulate between 5.57 and 18.47 mg/100 g, respectively.^34^ Nutritional or physiological effects of plastochromanol-8 in animals or humans have not been described so far. As a result of the non-enzymatic oxidation of plastochromanol-8 by singlet oxygen, hydroxy-plastochromanol (28) was identified in *Arabidopsis* leaves.^35^ Solanachromene (29) (plastochromenol-8) contains a double bond in the chromanol ring and was found in relatively high amounts (0.05% of dry weight) in aged flue-cured tobacco leaves.^33,36^

**Fig. 5:**
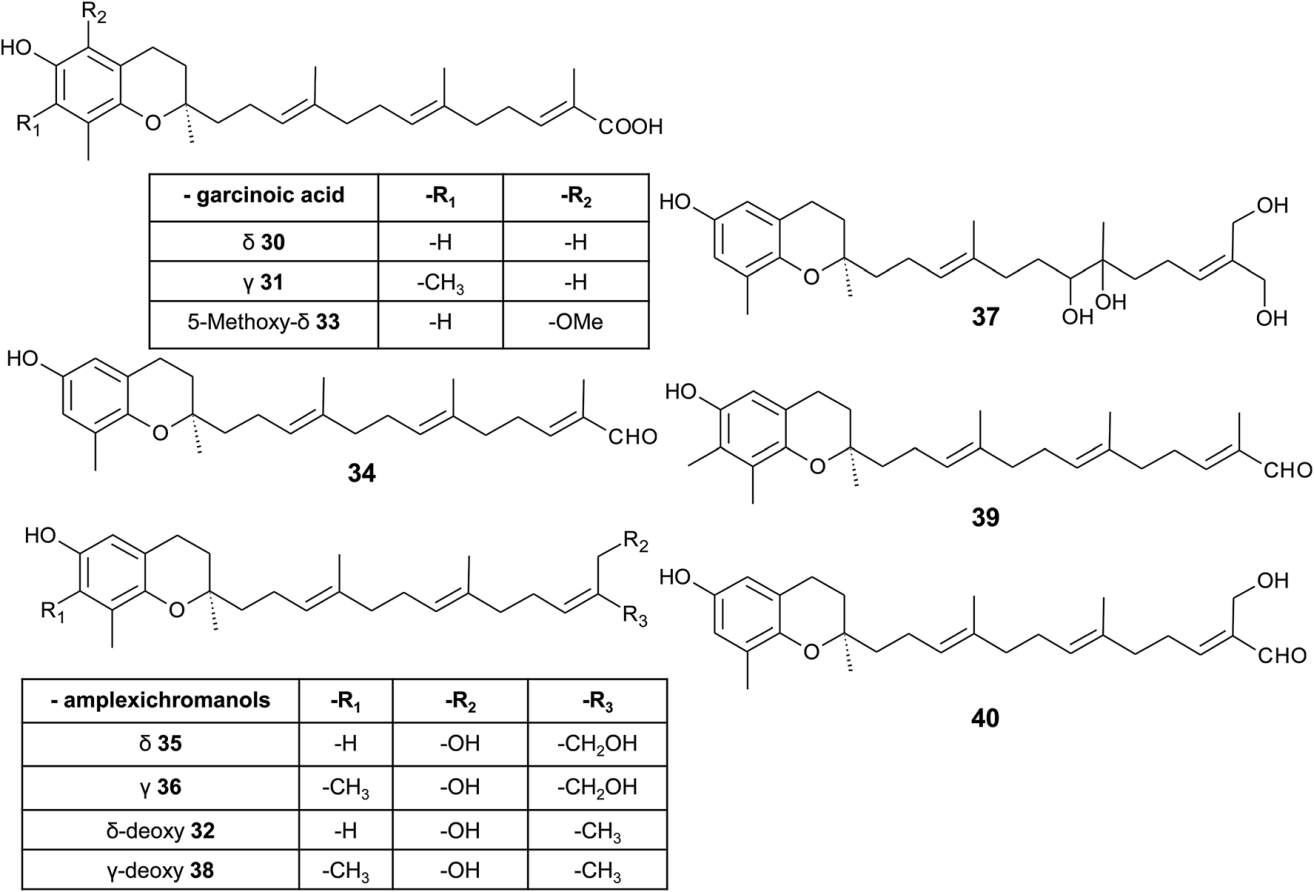
Structures and substitution patterns of garcinoic acids (30 to 33) and meroditerpenes (34 to -40) from *Garcinia amplexicaulis*.

δ-Garcinoic acid (30) (*E*-13′-carboxy-δ-tocotrienol, δ-garcinoic acid), an oxidation product of δ-tocotrienol, is probably the most investigated plant tocotrienol with side chain modification, so far (Fig. 5).^37^ δ-Garcinoic acid was first isolated from *Clusia grandiflora* by Delle Monache *et al.* and later by Terashima *et al.* from the African bitter nut *Garcinia kola* and was further characterized for its chemical and physiological properties.^37–42^ It has been detected in different amounts within the Clusiaceae family including *Tovomitopsis psychotriifolia*, *Clusia obdeltifolia*, *Clusia burlemarxii*, *Clusia pernambucensis*, *Garcinia kola* and together with γ-garcinoic acid (31) in the bark of *Garcinia amplexicaulis*.^43,44^ Recently, γ-garcinoic acid was isolated in small amounts from the Algerian conifer *Cedrus atlantica* (Pinaceae).^45^ A mixture of 2(*Z*)-δ-garcinoic acid and 2(*E*)-δ-garcinoic acid was isolated from the stem of *Clusia obdeltifolia*.^46^

δ-Garcinoic acid exerts potent anti-inflammatory, anti-proliferatory and antibacterial properties (see corresponding sections). As a possible target for its anti-inflammatory action, microsomal prostaglandin E_2_ synthase has been identified recently.^44^ The two natural (δ- and γ-garcinoic acid) isoforms as well as semi-synthesized β- and α-garcinoic acid inhibited the enzyme with IC_50_ values of 6.7, 2.0, 2.8 and 7.8 μM, respectively.

δ-Garcinoic acid reduced the growth of C6 cells and RAW264.7 mouse macrophages with an EC_50_ of 10 μM and 5 μM, respectively.^37,38^ As demonstrated by Maloney and Hecht, δ-garcinoic acid inhibits DNA polymerase β with an IC_50_ of about 4 μM.^47^ Whether this inhibition is a useful approach to prevent growth of cancer cells needs to be elucidated.

As mentioned above, γ-garcinoic acid and δ-(*E*)-deoxy-amplexichromanol (32) (see below) were isolated together with a 5-methoxy-δ-garcinoic acid derivative (33) from *Cedrus atlantica* (Fig. 5).^45^ All compounds showed only moderate anti-bacterial activity against different bacterial strains (see Table 3).

As a by-product of the isolation of garcinoic acid, garcinal (34) (δ-(*E*)-garcinal), with a terminal aldehyde group, was found in the *G. kola* nut.^41^ So far, the bioactive properties of garcinal are unknown.

Another interesting group of side chain-modified compounds with large structural variability has been isolated from the bark of *Garcinia amplexicaulis*, an endemic shrub from New Caledonia. δ- and γ-amplexichromanol (35) and (36) are terminal-hydroxylated δ- and γ-tocotrienols, respectively, carrying two hydroxy-groups at carbon-13′ and -14′ (Fig. 5).^43^ Both compounds inhibited capillary formation of VEGF-induced human primary endothelial cells at 25 nM concentration. Interestingly, only δ-amplexichromanol decreased the adhesion of VEGF-induced human primary endothelial cells whereas γ-amplexichromanol had no significant effect, suggesting different modes of action. δ-Dihydroxy-amplexichromanol (37) results from dihydroxylation of the double bond between C-7′ and C-8′. Besides γ-(*Z*)- and γ-(*E*)-deoxy-amplexichromanol (38) as well as δ-(*Z*)- and δ-(*E*)-deoxy-amplexichromanol, two aldehydes, namely γ-(*E*)-deoxy-amplexichromanal (39) (which is identical to γ-(*E*)-garcinal) and δ-(*E*)-amplexichromanal (40) were isolated from *Garcinia amplexicaulis*.^43,48^ δ-(*E*)-Deoxy-amplexichromanol (32) has also been described in *Cedrus atlantica*.^45^ δ-(*Z*)-Deoxy-amplexichromanol was earlier described by Teixeira *et al.* in *Clusia obdeltifolia*.^46^ In addition, dimeric oxidation and condensation products of amplexichromanols have been characterized.^43^

**Fig. 6:**
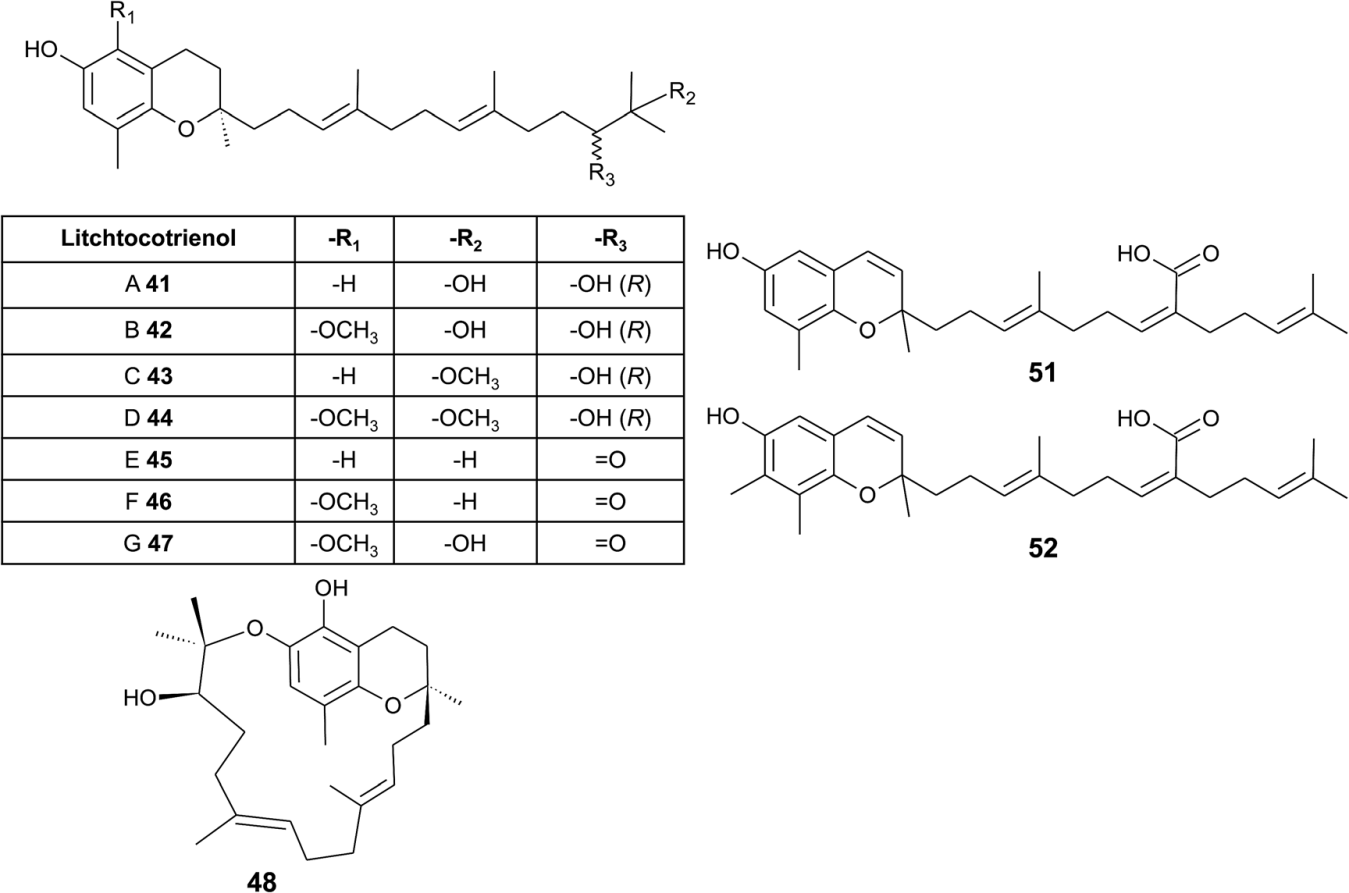
Structures and substitution patterns of litchtocotrienols (41 to 48) from *Litchi chinensis*, sargachromenols (51) and (52).

**Fig. 7:**
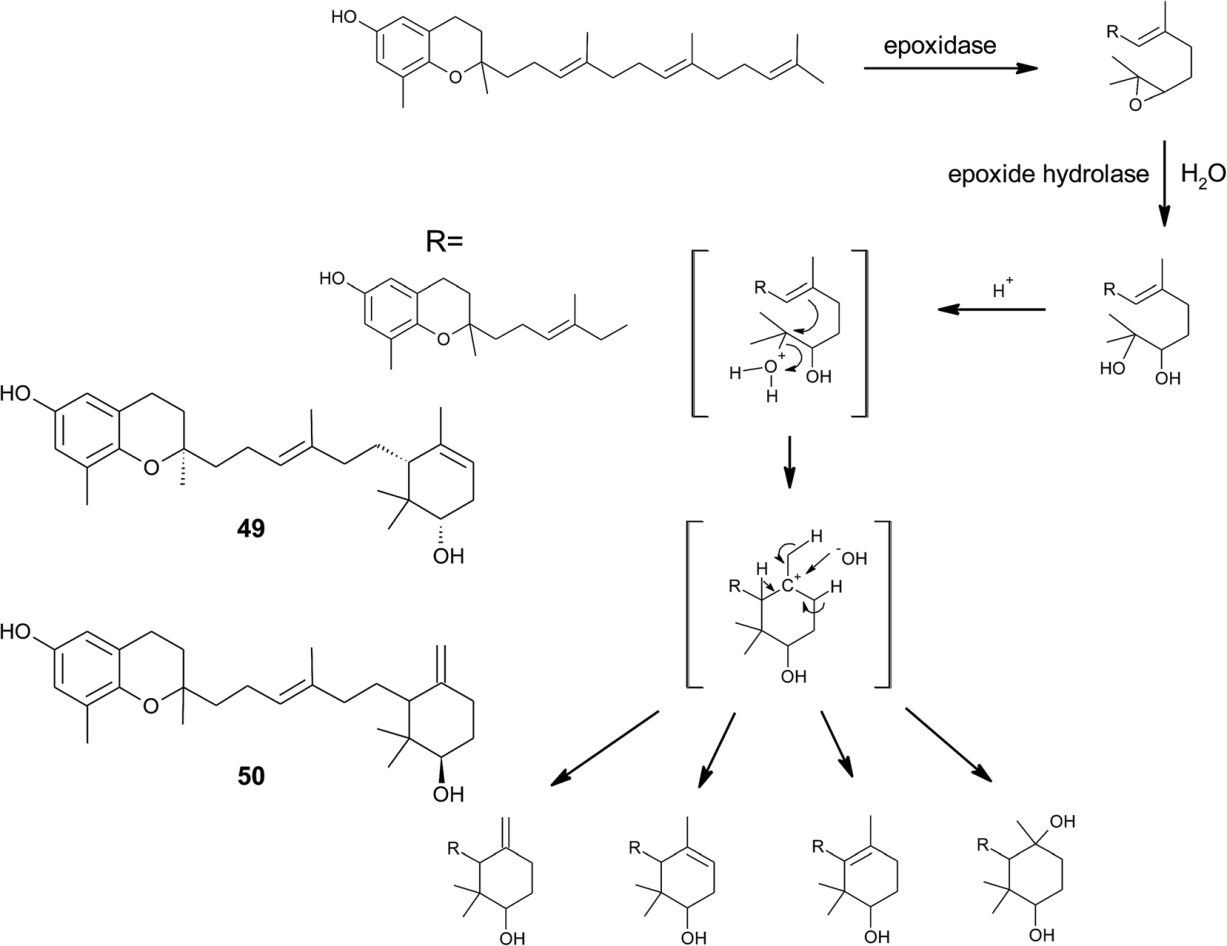
Scheme of an acid-catalyzed cyclization cascade including final cyclization of the chromane ring according to Etse *et al.*^61^ Examples are cyclolitchtocotrienol (49) and walsurol (50).

From the methanolic extract of leaves of *Litchi chinensis* (Sapindaceae), several δ-tocotrienol derivatives with side chain and chromanol modifications were isolated and investigated for their anti-cancerogenic potential.^49^ Litchtocotrienols A–G (41–47) are hydroxylated at C-11′ with *R*-configuration and E-F (45, 46) contain a ketone group at C-11′ (Fig. 6). An additional methoxy-group is introduced at position C-5 of the chromane ring for litchocotrienols B, D, F and G, respectively. Position C-12′ is hydroxylated for A, B, G or methoxylated for C and D. Macrolitchtocotrienol A (48) derives from an intramolecular condensation between C-12′ and C-6 to form an ansa-chromane. The structural motive is similar to the smenochromene sesquiterpenes. Finally, cyclolitchtocotrienol A (49) with a cyclohexene ring within the side chain was isolated. The latter compound is a structural isomer of walsurol (50) with related biosynthesis (Fig. 7). Litchtocotrienols presumably derive from the precursor 11′-12′-epoxide that undergoes nucleophilic ring opening and further modifications. Litchtocotrienols A–G and macrolitchtocotrienol A showed moderate cytotoxicity in HepG2 liver cells and gastric epithelial cells (AGS), with IC_50_ values ranging from 10–50 μM (Table 2).

All isolated compounds from *Garcinia amplexicaulis* and *Litchi chinensis* show high structural similarity to tocochromanols from *Saragassum* species (see section on Algae). In conclusion, *Garcinia amplexicaulis* and *Litchi chinensis* present the highest degree of structural variability among angiosperms.

Side chain-modified tocochromanols have been found in the fruits of the Amazonian Myristicaceae *Iryanthera juruensis* and *Iryanthera grandis*,^50–52^ and in vegetal parts of the Mexican Asteraceae *Roldana barba-johannis*.^53^*Iryanthera* leaves were used by the indigenous population to treat infected wounds and cuts, and the latex of the bark was used against infections.^52^ δ-Sargachromenol (51) was found in all the above-mentioned plants and was obtained in 0.4% and 0.8% yield (dry mass) from *Roldana* and *Iryanthera* species, respectively. δ-Sargachromenol is a δ-dehydrotocotrienol derivative with a carboxyl group located at C-15′ of the side chain and is thus a structurally related form of δ-garcinoic acid (30) (Fig. 5). Sargachromenol was named after the brown algae *Sargassum serratifolium*, from which it was first isolated by Kusumi *et al.*^54^ For a detailed description of the biological properties, please see the section on Algae.

Besides δ-sargachromenol, 7-methyl-sargachromenol (52) (γ-sargachromenol) was isolated from the fruits of *Iryanthera juruensis* by Silva *et al.*^50^

To the best of our knowledge, besides cyclolitchtocotrienol A (49), walsurol (50) obtained from the bark of the Yunnan tree *Walsura yunnanensis* (Meliaceae) is the only meroditerpene in higher plants that forms a 6-membered ring structure within the side chain.^55^ Interestingly, walsurol was obtained as the main lipid constituent from powdered bark (0.08% yield). Here, the authors discussed a possible mechanism that leads to cyclization reactions in the side chain. Epoxidation of the terminal double bonds in isoprenoid structures are well described for squalene and also for tocotrienols.^56,57^ Nucleophilic ring-opening results in a 11′,12′-diol structure that has also been described for algae.^58–60^ Etse *et al.* proposed an acid-catalyzed rearrangement that leads to a variety of cyclic structures formed from the diol. Elimination of water and ring closure between carbon 7′ and 12′ forms endo- (*e.g.* (49).) and exo-double bonds (*e.g.* (50)), respectively (Fig. 7).^61^ The metabolic pathway described here also applies to the formation of chromarols (see section on sponges).

### 3.2 Fungi

Although mushrooms and fungi produce a large number and variety of meroterpenoids,^62,63^ our database search found only scarce information on long-chain or cyclic 6-hydroxy-chromanols or -chromenes. The occurrence of α-, β-, γ-, and δ-tocopherols has been summarized in a review by Ferreira *et al.*^64^ Interestingly, no tocotrienols have been found in fungi so far. Several meroterpenoid structures were described with a 5-hydroxy-chromene ring, which originated from orsellinic acid as the aromatic precursor.^62^

### 3.3 Marine organisms

Since 1960, more than 20 000 distinct chemical compounds were discovered from marine organisms.^65^ Of these, algae and sponges form two third of all natural marine products found from 1965 to 2007.^66^ Marine natural products (MNP) with isoprenoid structures account for almost 60% of all natural products found in marine organisms.^67^ Several excellent reviews have summarized meroterpene structures from marine fungi,^68^ invertebrates,^69^ and algae.^67,70,71^ Tocopherols are well known to be produced by algae as well as marine invertebrates and microorganisms.^69,72^ Most interestingly, δ-tocotrienol (17) is widely distributed (especially in algae and sponges) and appears as the lead structure of most of the diverse compounds described in this review. Among them, sargachromanols, sargachromenols, cystoseira metabolites, chromarols, epitaondiols, smenochromenes and strongylophorines constitute the largest and best studied groups. Anti-bacterial, anti-viral, anti-inflammatory and cytotoxic properties were attributed to these compounds, making them potential lead structures for drug development.^73^

#### 3.3.1 Brown algae (Phaeophyceae)

Brown algae (Phaeophyceae) consist of around 2000 species of which the family of Sargassaceae, Dictypophycidae and Fucaceae produce most of the meroditerpenes described here.^74^

There is increasing interest in and knowledge about the isolation, and structural elucidation of meroditerpenes and their quinone precursors from brown algae. Recently, Culioli and colleagues described the analytical procedure for the extraction, chromatographic isolation and structural determination by sophisticated one- and two-dimensional nuclear magnetic resonance spectroscopic methods.^74^

As mentioned above, sargachromanols and sargachromenols show the highest structural diversity among all meroditerpenes. They derive from the common precursor geranylgeranyltoluquinol and subsequently from δ-tocotrienol and δ-dehydro-tocotrienol, respectively. δ-Tocotrienol-11′-12′-epoxide (53) was one of the first sargachromanols discovered in brown algae by Kato *et al.* in 1975.^57^ The activation of the terminal double bond leads to hydroxyl-, oxo-, and cyclic derivatives, respectively. However, the sequence of the chemical reactions leading to cyclic derivatives remains elusive (see also Fig. 7). Observational studies showed that an extract of *Sargassum tortile* induced the settling of swimming larvae of the hydrozoa *Coryne uchidai*, thus obviously acting as an intercellular signaling molecule.^75^ The epoxide was found by bioactivity-guided fractionation of the lipid extract.

In 2005, Jang *et al.* isolated a series of sargachromanols (A to P) (54–69) from *Sargassum siliquastrum* and characterized them by extensive two-dimensional nuclear magnetic resonance experiments.^76^ Later, Lee *et al.* isolated the structures Q to S (70–72) from the same species.^77^ Since sargachromanols A, B and S are sesquiterpenes, they are described here in the corresponding section.

**Fig. 8:**
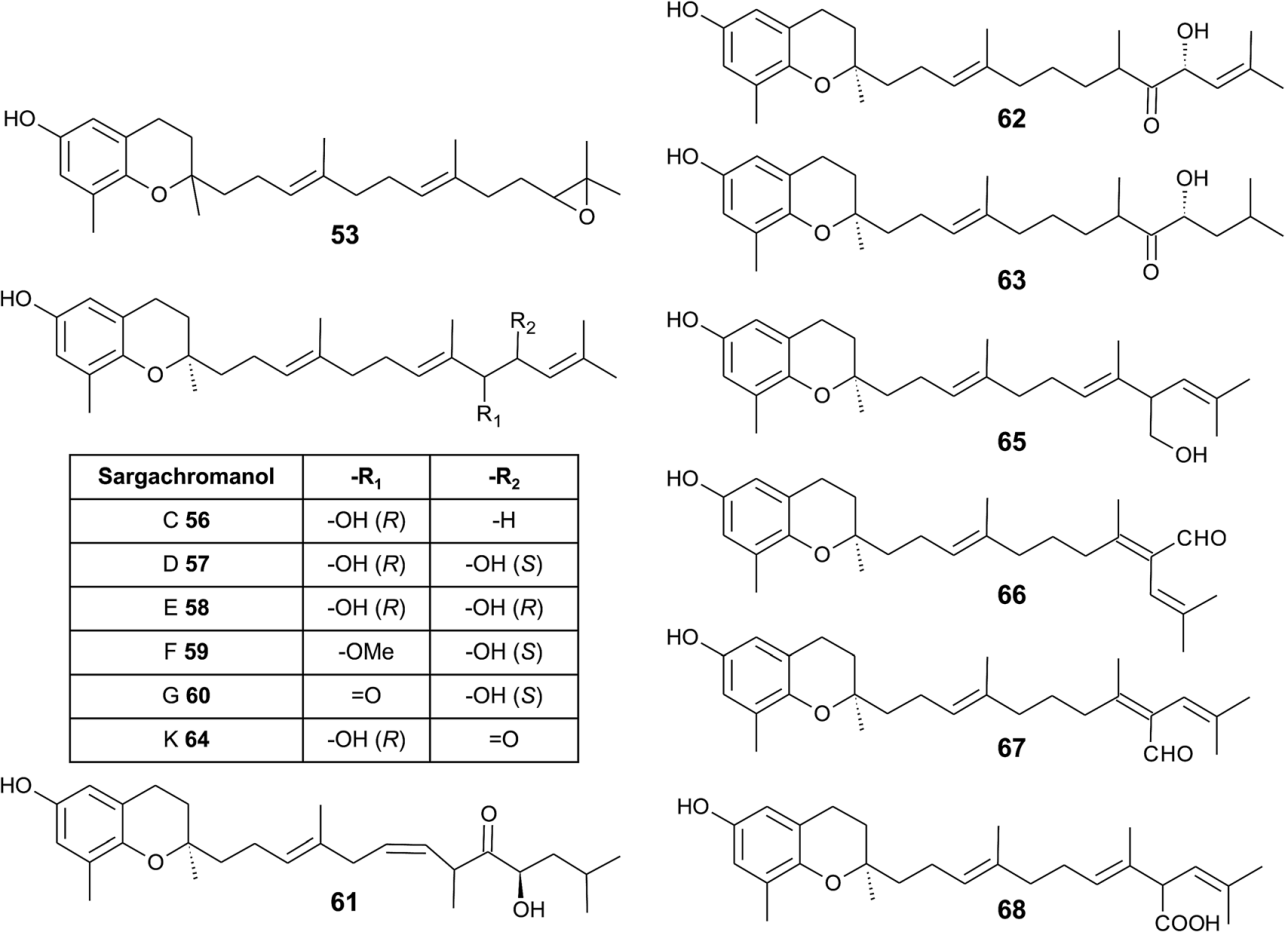
Structures and substitution patterns of sargachromanols (56 to 68) from *Sargassum* species.

Sargachromanol C (56) contains a 9′-hydroxyl group with *R*-configuration in the δ-tocotrienol side chain. The two diols, sargachromanols D (57) and E (58) possess hydroxyl groups at C-9′ and C-10′ and are diastereomers of each other. Sargachromanol F (59) has a methoxy group at C-9′ and a hydroxyl group with *R*-configuration at C-10′. Sargachromanols G to J (60–63) share similar side chain modifications consisting of a C-9′ carbonyl and a C-10′ hydroxyl group. They differ in the numer and type of saturation of the double bonds between C-7′ and C-8′ (I, J) (62, 63) and between C-11′ and C-12′ (H, J) (61, 63), respectively, and a double bond shift from C-7′ to C-6′ (H) (61). Sargachromanol K (64) is an isomer of sargachromanol G, where the carbonyl and hydroxyl groups are shifted to C-10′ and C-9′, respectively. Two-dimensional nuclear magnetic resonance experiments revealed that sargachromanols L to P underwent carbon skeleton rearrangements of the terminal prenyl group. Thus, the C-8′–C-9′ bond is rearranged to C-8′–C-10′. Sargachromanol L (65) contains a hydroxyl group at C-9′, whereas sargachromanols M (66) and N (67) are structural *cis*/*trans*-isomers containing an aldehyde group at C-9′. In addition, the double bond from C-7′ migrated to C-8′–C-10′. Further oxidation of sargachromanol L leads to sargachromanol O (68), which bears a carboxyl group at C-9′ und is thus a structural isomer of δ-garcinoic acid (30) (Fig. 8). The highest isolation yield was obtained for sargachromanols G (60) and I (62) (0.062 and 0.04%, respectively).^76^

**Fig. 9:**
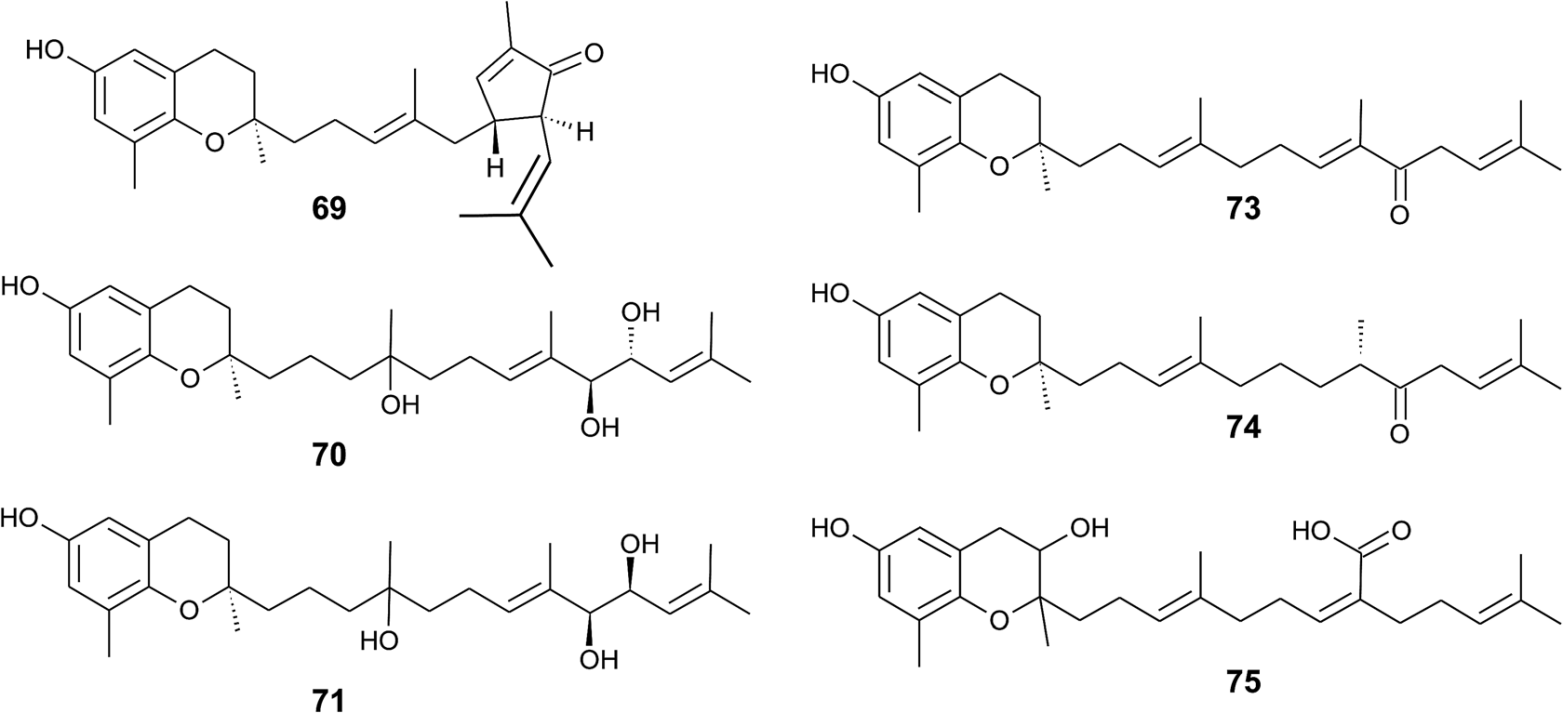
Structures and substitution patterns of sargachromanols (69 to 75) from *Sargassum* species.

The formation of an α,β-unsaturated cyclopentenone within the side chain leads to sargachromanol P (69). Sargachromanols Q (70) and R (71) share high structural similarity to sargachromanols D and E, respectively, but bear an additional *tert*-hydroxyl group at the saturated C-4′ (Fig. 9).^77^

Sargachromanols D, F, H and L are strong Na^+^/K^+^-ATPase ion pump inhibitors, with IC_50_ values of 3.6, 6.0, 4.6 and 7.0 μM, respectively.^78^ The study revealed that the hydroxyl groups at C-9′ and/or C-10′ are important for this inhibitory activity. Bioassay-guided fractionation of *Sargassum siliquastrum* extracts revealed anti-inflammatory action of sargachromanol D.^79^ The compound reduced lipopolysaccharide (LPS)-induced production of nitric oxide and prostaglandin (PG) E_2_ in murine RAW 264.7 macrophages and inhibited the expression of the pro-inflammatory enzymes inducible nitric oxide synthetase (iNOS) and COX-2. In addition, the production of the pro-inflammatory cytokines TNF-α, interleukin-1β (IL)-1β and IL-6 was reduced by sargachromanol D.^79^ Recently, sargachromanol D was suggested as an anti-hypertensive agent, since it showed dual antagonistic activity towards an L-type Ca^2+^-channel and endothelin A/B_2_ receptor (Table 3).^80^ The use of sargachromanols is protected by several patents.^81^

*Sargassum siliquastrum* was also used as a natural source of sargachromanol E and G for bioactivity studies.^82–87^ Both compounds inhibited the expression of pro-inflammatory cytokines in LPS-stimulated murine RAW 264.7 macrophages.^83,84,86^ In addition, sargachromanol E induced apoptosis *via* caspase-3 activation in promyelocytic HL-60 leukemia cells^82^ and inhibited ultraviolet A-induced ageing of human dermal fibroblasts.^88^ Sargachromanol G showed anti-osteoclastogenic effects on the expression of IL-1β-induced osteoclastogenic factors in the human osteoblast cell line MG-63 and suppressed the activation of nuclear factor κB (NF-κB) and mitogen-activated protein kinase (MAPK) in receptor activator of NF-κB ligand (RANKL)-induced RAW264.7 cells.^85,86^

Besides sargachromanol I and K, another two sargachromanols, (2*R*)-9′-oxo-δ-tocotrienol (73) and (2*R*)-7′-8′-dihydro-9′-oxo-δ-tocotrienol (74) were isolated from *Sargassum micracanthum*, however, in very low yield (Fig. 9).^89^

Seo *et al.* isolated a racemic mixture of thunbergol A (75) from *Sargassum thunbergii*. The compound features a 3-hydroxyhydrobenzopyran structure with a 15′-carboxy group and thus presumably derives from sargachromenol (Fig. 9).^90^

**Fig. 10:**
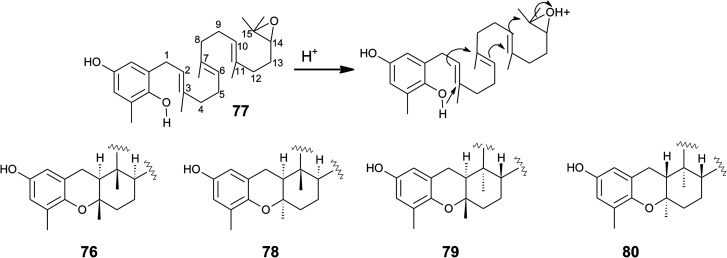
Proposed enzyme-catalyzed cyclization leading to cyclic sargachromanols (76 to 80).

Cyclic sargachromanols are widely distributed in brown algae. Taondiol (76) (Fig. 10) was the first cyclic side chain-derivative of tocotrienol that was isolated in 0.05% yield from *Taonia atomaria* (order Dictyotales).^91^ The authors proposed an enzyme-initiated synchronous cyclization cascade of the prenylated 1,4-hydroquinone leading to the tetracyclic ring system. We and others propose an alternative cyclization mechanism starting from 1,4-hydroquinone-14-15-epoxide (77), analogous to lanosterol synthesis^92,93^ (Fig. 10). The protonation of 77*via* an epoxide-hydrolase enzyme would increase the susceptibility of intramolecular attacks of the C-2–C-3 and C-6–C-7 double bonds. The stereochemistry of the possible isomers of taondiol at C-2 and C-3 and C-6 and C-7 has been a matter of debate. Recently, Areche *et al.* assigned the stereochemistry of isoepitaondiol (78) isolated from *Stypopodium flabelliforme* to the formerly described isotaondiol.^94^ By now, the structures of taondiol, isoepitaondiol, epitaondiol (79) and 2β,3α-epitaondiol (80) (Fig. 10) have been unambiguously assigned.^94–96^

Epitaondiol (79) was isolated from *Stypopodium zonale* and *Stypopodium flabelliforme* (both species are members of the order *Dictyotales*) and its bioactivity was intensively studied.^93,95–102^ The polycyclic compound shows ichthyotoxic, anti-herpes and anti-human metapneumovirus (HMPV) activity and acts as an anti-inflammatory agent *in vitro* and *in vivo* (see Tables 1–3).^98,99,102^ Further, epitaondiol inhibited cell proliferation of human colon adenocarcinoma (Caco-2), human neuroblastoma (SH-SY5Y), rat basophilic leukemia (RBL-2H3) cells, and murine macrophages (RAW.267), but not of non-cancer Chinese hamster fibroblasts (V79) (Table 2).^100^ 2β,3α-Epitaondiol (80) exhibited moderate neurotoxicity towards mouse neuro-2a cells with LC_50_ values of 2 μM.^92^ Epitaondiol was effective in the prevention of HCl/ethanol-induced gastric lesions in mice at an ED_50_ value of 40 mg kg^−1^ bodyweight.^101,103^ Anti-insecticide activity was found against *Spodoptera frugiperda*.^96^ Finally, the compound induced the settlement of the mussel *Perna perna*.^98^

**Table 1:** Inhibition of inflammatory markers by chromanols and chromenols^a^

Compounds	Test system	Effective concentrations^b^	References
***Meroditerpenes***			
α-Tocopherol (3)	IL-1β-stimulated A549 cells	8% nitric oxide inhibition at 10 μM	279
		25% iNOS inhibition at 10 μM	
		IC_50_ PGE_2_ inhibition: > 50 μM (A549 cells)	
γ-Tocopherol (5)	IL-1β-stimulated A549 cells	IC_50_ PGE_2_ inhibition: 7.5 μM (RAW264.7)	279
		IC_50_ 5-LOX inhibition: > 50 μM	245,257
		IC_50_ COX-2 (A549 cells) inhibition: > 50 μM	
δ-Tocopherol (6)	IL-1β-stimulated A549 cells	IC_50_ COX-2 (A549 cells)) inhibition: > 50 μM	257
		IC_50_ 5-LOX inhibition: > 50 μM	
		IC_50_ PGE_2_ inhibition: 3 μM (A549 cells)	
α-Tocotrienol (14)	LPS-induced RAW 264.7 cells	5% NO inhibition at 33 μM	280
δ-Tocotrienol (17)		31% NO inhibition at 26 μM	
γ-Tocotrienol (16)		19% NO inhibition at 30 μM	
	IL-1β-stimulated A549 cells	IC_50_ PGE_2_ inhibition: 1 μM (A549 cells)	245
13′-carboxy-α-tocopherol (205) (α-13′-COOH)	LPS-induced RAW 264.7 cells	Total inhibition at 2.7 μM	255
		IC_50_ NO production: 0.2–0.5 μM^b^	
	LPS-induced RAW 264.7 cells	88% NO inhibition at 5 μM	256
		100% iNOS inhibition at 5 μM	
13′-carboxy-δ-tocopherol (229) (δ-13′-COOH)	Inhibition of COX-1 and COX-2	IC_50_ COX-1 (bovine) inhibition: 5.0 μM	245
		IC_50_ COX-2 (human) inhibition: 4.0 μM	245
		IC_50_ COX-2 (A549 cells)) inhibition: 4.0 μM	257
	Inhibition of human recombinant 5-LOX	IC_50_ 5-LOX inhibition: 0.5–1.0 μM	253
		IC_50_ (LTB_4_) generation: 4–7 μM	
	Neutrophils and promyelocytic HL-60		
	Leukemia cells generated LTB_4_	79% NO inhibition at 5 μM	
	LPS-induced RAW 264.7 cells	56% iNOS inhibition at 5 μM	256
13′-Hydroxy-α-tocopherol (204) (α-13′-OH)	LPS-induced RAW 264.7 cells	49% COX-2 inhibition at 10 μM	241,256
		53–60% iNOS inhibition at 10 μM	
		54% PGE_2_ inhibition at 10 μM	
		44–69% NO inhibition at 10 μM	
13′-Hydroxy-δ-tocopherol (231) (δ-13′-OH)	LPS-induced RAW 264.7 cells	49% NO inhibition at 10 μM	256
		53% iNOS inhibition at 10 μM	
δ-Garcinoic acid (30)	Inhibition of COX-2 and 5-LOX	IC_50_ COX-2 inhibition: 9.8 μM	257
		IC_50_ 5-LOX inhibition: 1.0 μM	
	Inhibition of LPS-stimulated NO production in RAW 264.7 macrophages	IC_50_ NO production: 1.0 μM^b^	260
α-, β-,< γ-,< δ-Garcinoic acid (209, 232, 231, 230)	Inhibition of PGE2-synthase (PGES-1)	IC_50_ 7.8 (α-), 2.8 (β-), 2.0 (γ-), 6.7 (δ-) garcinoic acid	44
δ-Sargachromenol (51)	TPA-induced mouse ear edema	IC_50_ edema reduction: 0.36 mg per ear	53
	Inhibition of COX-1 and -2	98% COX-1 inhibition at 100 ppm	52
		84% COX-2 inhibition at 100 ppm	
	LPS-induced RAW 264.7 and BV-2 cells	IC_50_ NO production: 82 μM (RAW264.7)	129
		IC_50_ PGE_2_ inhibition: 30.2 μM (RAW264.7)	
		IC_50_ NO production: 1.3–2.7 μM (BV-2)	134,261
Sargachromanol D (57)	LPS-induced RAW 264.7 cells	IC_50_ NO production: 40 μM^b^ (RAW264.7)	79
		IC_50_ PGE_2_ inhibition: 15 μM^b^ (RAW264.7)	
Sargachromanol E (58)	LPS-induced RAW 264.7 cells	IC_50_ NO production: 16.3 μM	83
Sargachromanol G (60)	LPS-induced RAW264.7 cells	IC_50_ NO production: Ca. 15 μM^b^	84
Chromarols (A–D) (113–116) *cyclic*	Inhibitors of 12- and 15-LOX	15-hLO IC_50_: 0.6(A), 4.0(B), 0.7(C), 1.1 μM(D)	154
		12-hLO IC_50_: all >100 μM	
Epitaondiol (79) *cyclic*	TPA-induced mouse ear edema	IC_50_ edema reduction: 20.7 μg per ear	99
		IC_50_ myeloperoxidase activity: 17.8 μg per ear	
	Eicosanoid inhibition	IC_50_ (TXB_2_) generation: 3.8 μM	99
		IC_50_ (LTB_4_) generation: 30.1 μM	

***Merosesquiterpenes***			
Capillobenzopyranol (172) *cyclic*	LPS-induced RAW 264.7 cells	36.7% NO inhibition at 10 μM	202
9′-Carboxy-δ-tocopherol (206) (δ-9′-COOH)	Inhibition of COX-1, -2	IC_50_ COX-1 (bovine) inhibition: >20 μM	245
		IC_50_ COX-2 (human) inhibition: >20 μM	
		IC_50_ COX-2 (A549 cells)) inhibition: 6.0 μM	

***Monoterpenes***			
Cordiachromene A (173)	Inhibition of PGI_2_ biosynthesis	IC_50_ 8.2 μM	216
	Carrageenan induced rat paw endema	IC_50_ 18.9 μM	211
	Inhibitor of 15-LOX	IC_50_: 0.82 μM	214
		IC_50_: 2 μM	
α-CMBHC (207)		IC_50_ COX-1 (bovine) inhibition: 160 μM	245
		IC_50_ COX-2 (human) inhibition: 140 μM	
α-CEHC (208)	TNFα-stimulated NO and PGE_2_ production in RAEC cells	IC_50_ PGE_2_ inhibition: 59 μM	244
		IC_50_ NO production: 56 μM	
γ-CEHC (233)		IC_50_ COX-1 (bovine) inhibition: 300 μM	245
		IC_50_ COX-2 (human) inhibition: 450 μM	
		IC_50_ COX-2 (A549 cells)) inhibition: 35–70 μM	
		IC_50_ PGE_2_ inhibition: 30.0 μM (RAW 264.7)	279

***Hemiterpenes***			
Quercinol (199)	*In vitro* cytokine inhibition	IC_50_ COX-1 inhibition: 4.7 μM	235
		IC_50_ COX-2 inhibition: 0.63 μM	
		IC_50_ 3α-HSD inhibition: 114 μM	
		IC_50_ XO inhibition: 21 μM	
		IC_50_ HRP: 68 μM	

a Abbreviations: thromboxane B_2_ (TXB_2_), leukotriene B_4_ (LTB_4_), cyclooxygenase (COX)-1 and -2, 3α-hydroxysteroid dehydrogenase (3α-HSD), lipoxygenase (LOX), xanthine oxidase (XO), horseradish peroxidase (HRP).

b Estimated from original publication.

**Table 2:** Cytotoxic activities of by chromanols and chromenols against cancer cells^a^

Compound	Isolated from	Cell line/organism	Effective concentration^c^	Reference
***Meroditerpenes***				
α-Tocopherol (3)	Plant oils	HepG2 cells,	>100 μM	39
		MDA-MB-231, MCF7 cells	Not achieved	281
γ-Tocopherol (5)	Plant oils	Jurkat, HBTII, MCF7, MCF7-C3 cells	>50 μM	267
δ-Tocopherol (6)	Plant oils	Jurkat, HBTII, MCF7, MCF7-C3 cells	>50 μM	267
α-Tocotrienol (14)	Palm oil	MDA-MB-435, MCF7, B16 cells	IC_50_: 210 μM, 14 μM, 110 μM	25,273
		MDA-MB-231, MCF7 cells	IC_50_: 24 μM, 26 μM	281
γ-Tocotrienol (16)		Jurkat, HBTII, MCF7, MCF7-C3 cells	∼50%, 35%, 30%, 35% at 50 μM	267
		MDA-MB-231, MCF7 cells	IC_50_: 11 μM, 15.6 μM	281
		SKBR3 cells, BT474 cells	IC_50_: 4.1 μM, 4.4 μM	275
δ-Tocotrienol (17)		MCF7, B16 cells	IC_50_: 15 μM, 10 μM	25,276
		MDA-MB-231, MCF7 cells	IC_50_: 17 μM, 17 μM	281
Sargaol (95)	*Stypopodium flabelliforme*	Human epithelial gastric cells	IC_50_: 18 μM	103
		Human fibroblasts	IC_50_: 12 μM	
	*Sargassum tortile*	P338 leukemia cells	EC_50_: 52 μM	120
Desmethyltocotrienol (18) (P_21_-tocotrienol)	Rice bran	B16 cells (suppression of proliferation)	IC_50_ > 1 μM^b^	24
Didesmethyltocotrienol (19) (P_25_-tocotrienol)			IC_50_: 0.9 μM	25
13′-Carboxy-δ-tocopherol (229)	Semisynthetic from *Garcinia kola*, human metabolites	Glioma C6 cells	n.d.	38
		HepG2 cells	EC_50_: 6.5 μM (δ-13-COOH)	39
		THP-1 macrophages	EC_50_: 11.1 μM (δ-13-COOH)	277
		HCT-116 cells	EC_50_: 8.9 μM (δ-13-COOH)	257
		HT-29	EC_50_: 8.9 μM (δ-13-COOH)	
13′-Hydroxy-α-tocopherol (204)		HepG2 cells	EC_50_ > 100 μM	39
		THP-1 macrophages	EC_50_ > 100 μM	277
13′-Carboxy-α-tocopherol (205)		HepG2 cells	EC_50_: 13.5 μM (α-13-COOH)	39
		THP-1 macrophages	EC_50_: 7.4 μM (α-13-COOH)	277
δ-Garcinoic acid (30)	*Garcinia kola*	Glioma C6 cells	EC_50_: 10 μM	38
		RAW264.7 macrophages	EC_50_: 5.5 μM	255
		HCT-116 cells	EC_50_: 16 μM (δ-garcinoic acid)	257
		HT-29	EC_50_: 17 μM (δ-garcinoic acid)	
		Inhibition of DNA polymerase β	IC_50_: 4 μM	47
δ-Sargachromenol (51)	*Sargassum sagamiamum*	Caspase-3 induced apoptosis in HaCaT cells	EC_60_: 11.8 μM^b^	126
Fallachromenoic acid (105)	*Sargassum fallax*	P338 leukemia cells	IC_50_ > 27–29 μM	133
δ-Amplexichromanol (35)	*Garcinia amplexicaulis*	Antiangiogenicity in VEGF-induced HUVECs	Effective at 25 nM and 2.5 μM	43
γ-Amplexichromanol (36)				
Litchtocotrienol A-G (41–47)	*Litchi chinensis*	HepG2 cells	IC_50_: 11.1 (A), 14.2 (B), 22.7 (C), 10.7 (E), 12.3 (F), 34.1 (G) μM	49
		AGS cells	IC_50_: 10.9 (A), 32 (B), 24.2 (C), 26.8 (D), 27.4 (E), 49.2 (F) 43.2 (G) μM	
Crassumtocopherol A (134)	*Lobophytum crissum*	P338 leukemia cells	IC_50_: 6.7 μM	169
			IC_50_: 5.2 μM	
Crassumtocopherol B (135)		Cytotoxicity in HT-29 cells	IC_50_: 7.5 μM	
Sargachromanol E (58)	*Sargassum siliquastrum*	Caspase-3 induced apoptosis in promyelocytic HL-60 leukemia cells	EC_50_: 20 μM^b^	82
Sargatriol (98)	*Sargassum tortile*	P-338 leukemia cells	EC_50_: 42 μM	118
Sargadiol-I (96)			EC_50_: 34 μM	120
Sargadiol-II (97)			EC_50_: 41 μM	
Sargadiol I (96)	*Desmaretia menziesii*	*Artemia salina*	EC_50_: 233 μM	123
Epitaondiol (79) *cyclic*	*Stypopodium flabelliforme*	Human epithelial gastric cells	IC_50_: 29 μM	103
		Human fibroblasts	IC_50_: 19 μM	
		RAW 264.7	IC_50_: 12.7 μM	100
Isoepitaondiol (78) *cyclic*		Human epithelial gastric cells	IC_50_: 42 μM	103
		Human fibroblasts	IC_50_: 65 μM	
		Neuro-2a cell line	LC_50_: 2 μM	92
		NCI-H460	LC_50_: 24 μM	
Macrolitchtocotrienol A (48) *cyclic*	*Litchi chinensis*	HepG2 cells	IC_50_: 16.5 μM	49
Cystoseirol A (86) *cyclic*	*Cystoseira mediterranea*	Crown-gall potato bioassay	73% tumor inhibition at 10^−2^ M	110
Demethoxy cystoketal chromane (89) *cyclic*	*Cystoseira amentacea*	Cytotoxicity in HepG2 cells	IC_50_: 35 μM	113
	*Cystoseira tamariscifolia*			
Strongylophorine 2 (117)	*Petrosia corticata*	Cytotoxicity in HeLa cells	IC_50_: >100 μM	163
Strongylophorine 3 (118)			IC_50_: 45.2 μM	
Strongylophorine 4 (119)			IC_50_: 50.5 μM	
Strongylophorine 22 (128)			IC_50_: 26.6 μM	
Strongylophorine 23 (129)			IC_50_: 62.0 μM	
Strongylophorine 24 (130)			IC_50_: >100 μM	
*All cyclic*				

***Merosesquiterpenes***				
Riccardiphenol C (146) *cyclic*	*Riccardia crassa*	P338 leukemia cells	IC_50_ > 80 μM	177
Aureol (155) *cyclic*	*Smenospongia aurea*	A549,	IC_50_: 13.6 μM	189
		HT-29,	IC_50_: 14.9 μM	
		EL-4	IC_50_: 31.5 μM	
Panicein A2 (160) *cyclic*	*Reniera mucosa*	P388, A549, MEL20, HT29	EC_50_: 14.8 μM	195
Panicein F2 (161) *cyclic*			EC_50_: 14.2 μM	

***Monoterpenes***				
Cordiachromene A (173)	*Aplidium antillense*	Human carcinoma KB	CI_50_: 14.3 μM	212
		Murine leukemic P388	CI_50_: 20.5 μM	
	*Aplidium aff. densum*	Lymphoblastic leukemia CEM-WT cells	IC_50_: 30 μM	213
Didehydroconicol (185)	*Aplidium aff. densum*	Lymphoblastic leukemia CEM-WT cells	IC_50_: > 10 mM	213
Epiconicol (184)			IC_50_: 60 mM	
Didehydroconicol (185)	*Aplidium aff. densum*	Sea urchin eggs of *P. lividus* and *S. granularis*	IC_50_: >25 μM and 9.8 μM	218
Epiconicol (184)			IC_50_: >11.3 μM and >25 μM	
Chaetopyranin (188)	*Chaetomium globosum*	HMEC, SMMC-7721, A549	IC_50_: 49 μM, 90 μM, 124 μM	220
α-CEHC (208)	Human metabolites		Growth inhibition at 50 μM	282
γ-CEHC (233)		Prostate cancer PC-3	42% (α-), 83% (γ-)	
		HTB-82	34% (α-), 58% (γ-)	
		HECV	9% (α-), 19% (γ-)	
Sargasal-I (176)	*Sargassum tortile*	P-338 leukemia cells	EC_50_: 20.3 μM	118
Sargasal-II (177)			EC_50_: 21 μM	120

***Hemiterpenes***				
Mollugin (189)	*Rubica cordifolia*	MCF-7/adriamycin	IC_50_ of doxorubicin decreased from 60 to 7.5 μg ml^−1^ at 10 μM mollugin	226

a Abbreviation: human gastric adenocarcinoma cells (AGS), human microvascular endothelial cells (HMEC), hepatocellular carcinoma cells (SMMC-7721), human lung epithelial cells (A549), human hepatocellular carcinoma cells (HepG2), B16 melanoma cells, vascular endothelial growth factor (VEGF), human umbilical vein endothelial cells (HUVEC).

b Estimated from original publication.

c For better comparison μg ml^−1^ were converted to μM.

A series of cyclic meroditerpenes was isolated from different *Cystoseira* species collected along the Mediterranean and contiguous Atlantic coasts.^104^ According to AlgaeBase,^105^ more than 289 species (and infraspecific) names were found, of which 42 have been marked as currently accepted taxonomically.

**Fig. 11:**
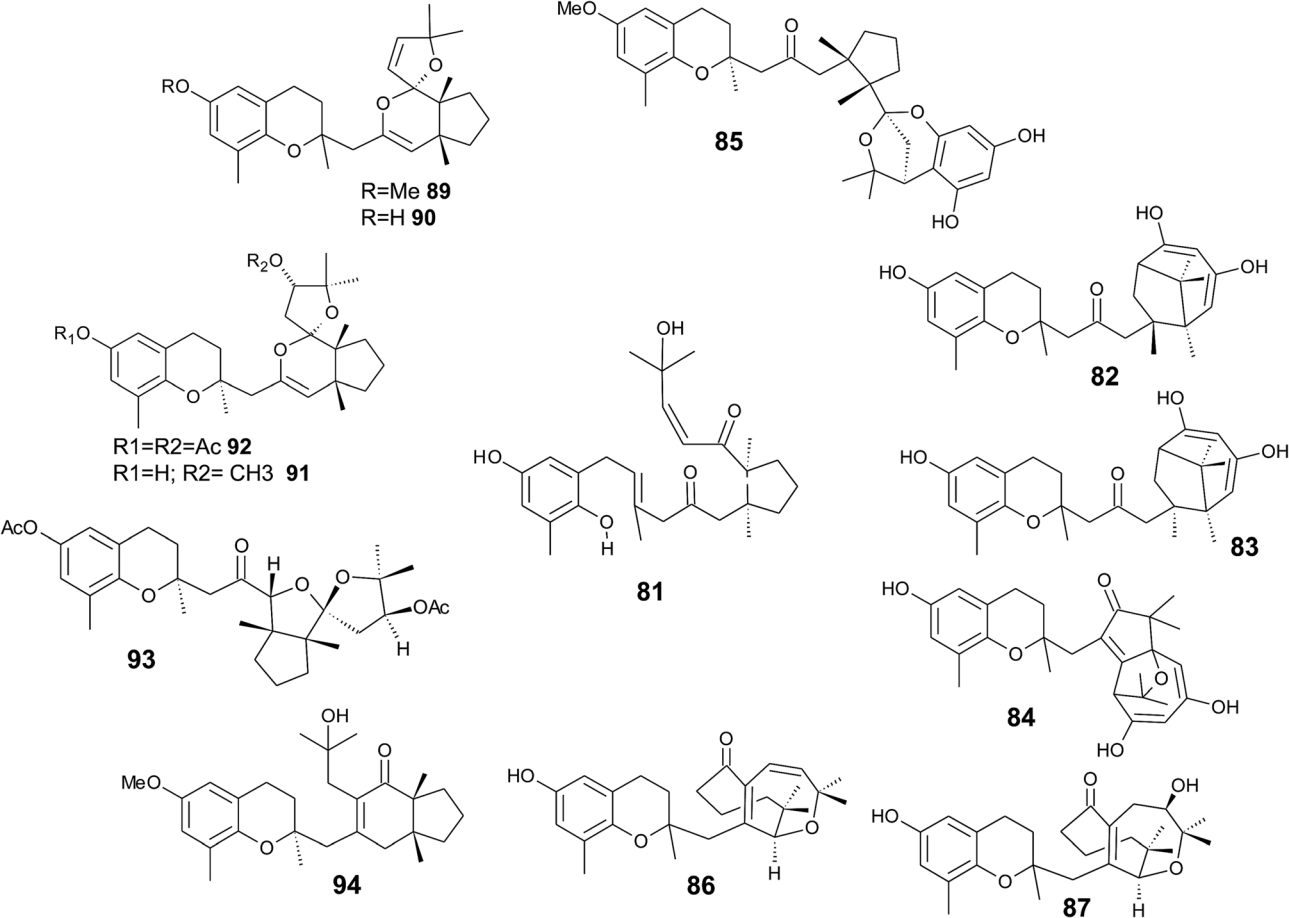
Structures of mediterraneols (82 to 84), cystophloroketal E (85), cystoseirols (86 to 88), chromanes (90 to 92), (94) and cystoseirone (93) from *Cystoseira* species. Bifurcarenone (81) as the common biosynthetic precursor is depicted in the center of the figure.

It was suggested that the following cyclic diterpenes origin from a common biosynthetic precursor, namely bifurcarenone (81) (Fig. 11). Among them, mediterraneols C (82), D (83), and E (84) have been isolated as their trimethoxy-derivatives from *Cystoseira mediterranea* in high yield (0.11, 0.14 and 2.0% from dry weight algae, respectively).^106,107^ Mediterraneols C and D are stereoisomers at C-4′ and compromise a bridged cyclooctane structure with two dienol moieties. Mediterraneol E (84) is a tricyclic oxygen-bridged diterpene with antineoplastic activity.^107^ So far, the biosynthesis of mediterraneols is largely unknown.^106,108^ Mediterraneols have been found to inhibit the mobility of sea urchin sperm and the mitotic cell division (ED_50_ values of 2 μg ml^−1^) of fertilized urchin eggs.^106^

Recently, cystophloroketal E (85), a meroditerpene with a 2,7-dioxabicylo[3.2.1]octane core was isolated from *Cystoseira tamariscifolia*.^108^ The authors assumed that ketal formation was preceded by a Michael addition of phloroglucinol onto the unsaturated carbonyl of 4-methoxy-bifurcarenone. The compound showed anti-bacterial, anti-microalgal and anti-invertebrate activity (Table 3).

**Table 3:** Miscellaneous biological activities of chromanols and chromenols^a^

Compound	Activity	Species	Effective concentration	reference
***Meroditerpenes***				
γ-Dehydrotocopherol (12)	Proliferation, wound healing			21
Desmethyltocotrienol (18) (P_21_-tocotrienol)	Hypocholesterolemic activity	Chicken	Reduction of total cholesterol (mmol L^−1^) *vs.* control diet: 26% and 31%	24
Didesmethyltocotrienol (19) (P_25_-tocotrienol)			Reduction of LDL cholesterol (mmol L^−1^) *vs.* control diet: 41% and 48%	
δ-Garcinoic acid (30)	Anti-bacterial	*Staphylococcus aureus*, *Bacillus cereus*, *Pseudomonas aeruginosa*	1 mm zone of inhibition	42
Sargaol (95)	Gastroprotective against HCl/ethanol-induced gastric lessions	Mice	30 mg kg^−1^	103
Taondiol (76)				
Sargachromanol D, F, H, L (57, 59, 61, 65)	Ion pump inhibitor Na^+^,K^+^-ATPase	*In vitro*	IC_50_: 3.6 μM, 6.0 μM, 4.6 μM, 7.0 μM	78
Epitaondiol (79)	Anti-viral	HSV-1	EC_50_: 1.34 M	98
Strongylophorine 3 (118)	Insecticidal activity	*Spodoptera littoralis*	EC_50_: 60 ppm	157
Bifurcarenone chromane (94)	Photoprotection	Human fibroblasts	IC_50_: 5–20 μg ml^−1^	116
Bifurcarenone chromene (107)	Anti-macroalgal	*Sargassum muticum*	IC_50_: 2.5 μg ml^−1^	143
Sargadiol-I (96)	Anthelmintic	*Nippostrongylus brasiliensis*	EC_50_: 307 μg ml^−1^	123
11′,12′-dihydroxy-3,4-dehydro-δ-tocotrienol (102)	Anti-viral	Human cytomegalovirus	IC_50_ virus absorption: 0.2 μM	60
			IC_50_ antiviral activity: 0.49 μM	58
		HSV-1	2.8 μM	
		HSV-2	2.6 μM	
		Mumps virus	7.6 μM	
		Measles virus	2.7 μM	
		Adeno virus	14 μM	
		Influenza virus	4.4 μM	
		Poliovirus	9.0 μM	
		Coxsackievirus	6.8 μM	
11′,12′-dihydroxy-3,4-dehydro-δ-tocotrienol (102)	Inhibition of bone resorption	Osteoclast-like cells (OCLs)	IC_50_: ∼8 μM^b^	59
Sarcochromenol sulfate A (110)	Ion pump inhibitor Na^+^,K^+^-ATPase		IC_50_: 1.6 μM	152

***Merosesquiterpenes***				
Chromazonarol (154)	Algicidal activity	*Heterosigma akashiwo*	Mean mortality after 4h at 1 μg ml^−1^:	182
		*Chattonella antiqua*	78%	
		*Heterocapsa circularisquama*	42%	
			93%	

***Monoterpenes***				
Cordiachromene A (173)	Anti-bacterial	*Staphylococcus aureus*	2–64 μg ml^−1^	212
		*Streptococcus faecalis*	1–64 μg ml^−1^	
		*Escherichia coli*	MIC > 2 mmol	213
		*Micrococcus luteus*	MIC > 0.51 mmol	
Didehydroconicol (185)	Anti-bacterial	*Escherichia coli*, *Micrococcus luteus*	MIC > 2 mmol, MIC > 0.51 mmol	213
Epiconicol (184)			MIC > 2 mmol, MIC > 0.13 mmol	
Cymobarbatol (181) and 4-isocymobarbatol (182)	Antimutagenic inhibition of 2AN and EMS mutagenicity towards *Salmonella thyphimurium*	T-98 stain	75, 150 and 300 μg/plate	208
		T-100 stain	(EMS): 32.5–300 μg/plate	

***Hemiterpenes***				
Precocene 2 (194)	Anti juvenile hormone	Induction of precocious metamorphosis in milkweed bug	0.7 μg cm^−2^ 90% precocious adults	230
Daedalin A (199)	Inhibition of melanin synthesis	Tyrosinase inhibition	IC_50_: 194 μM	234

a Abbreviations: herpes simplex virus 1 and 2 (HSV-1 and -2), minimum concentration that inhibits (MIC) bacterial growth, 2-aminoanthracene (2AN), ethyl methanesulfonate (EMS).

b Estimated from original publication.

Another group of complex bicyclic compounds was isolated from *Cystoseira stricta*, *Cystoseira mediterranea* and *Cystoseira tamariscifolia*. Cystoseirols A (86), B (87) and C (88) possess a oxabicyclo[5:4:1]dodecane ring that results from a single methyl group displacement, supplementary bridges and ring fissions.^107,109,110^ Cystoseirol A (86) inhibited plant tumor formation in a crown-gall potato bioassay (73% at 10 μM).^110^

Amico *et al.* isolated cystoketal chromane (89) from the Sicilian brown alga *Cystoseira balearica*. Structural elucidation revealed a tricyclic ring system within the side chain and an epimeric mixture at C-2 (Fig. 11). Thus, the authors proposed cystoketal chromane to be an artefact of the extraction process.^111^ Later, demethoxy cystoketal chromane (90) was isolated from Mediterranean *Cystoseira amentacea* and recently from *Cystoseira tamariscifolia*.^112,113^ Demethoxy cystoketal chromane showed cytotoxic activity with high selectively towards HepG2 cells (IC_50_ = 14.77 μg ml^−1^).^113^ A screening of 55 *Cystoseira* species found several bicyclic meroditerpenes, namely 14-methoxyamentol chromane (91), amentolchromane and cystoseirone, respectively.^114^ The latter two compounds were unstable and were therefore isolated as their acetate derivatives. Interestingly, isolated amentolchromane acetate (92) could be transferred to cystoseirone acetate (93) by chemical oxidation with *meta*-chloroperoxybenzoic acid in methylenchloride. Again, it was suggested that amentols and cystoseirone have a common biosynthetic precursor, bifurcarenone (81) bearing the typical *cis* orientation for the bridgehead methyls.

Finally, bifurcarenone chromane (94), the cyclization product of 81, was found in C*ystoseira baccata*,^104,115^ and *Sargassum muticum*,^116^ from which it was isolated as epimeric mixture at C-2 (Fig. 11). The mixture showed anti-leishmanial activity at IC_50_ values of 44.9 μM and decreased the intracellular infection index (IC_50_ value of 25.0 μM).^117^

**Fig. 12:**
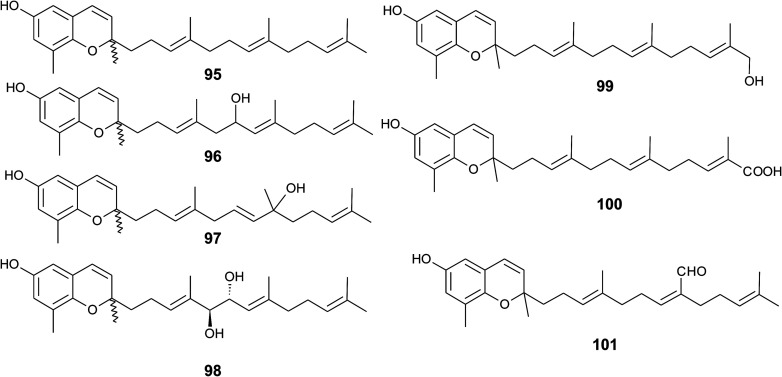
Structures of sargachromenols (95 to 101) from *Sargassum* species.

Sargaol (95) or dehydro-δ-tocotrienol is the potential biosynthetic precursor for most of the chromenols found in brown algae. It was originally isolated from *Sargassum tortile* collected at the Japanese Tanabe Bay. A lipid extract of the algae exhibited high cytotoxic activity and was used as a skin-lightening agent.^118,119^ Fractionation of the extract resulted in the isolation of sargaol (95), sargadiols-I (96) and -II (97), and sargatriol (98) (Fig. 12).^120,121^ All compounds were moderately cytotoxic towards murine P-388 leukemia cells with ED_50_ values of 52, 34, 41 and 42 μM, respectively (Table 2).^118,120^ Sargadiols (96) and (97) bear a hydroxyl group at C-6′ and C-8′, respectively, and sargatriol has two hydroxyl groups at C-5′ and C-6′. All compounds were suggested to be artefacts of the isolation since epimers at C-2 were found in all cases. In addition, heating of the corresponding 1,4-hydroquinones in organic solvents led to the epimeric chromenes described in this paragraph.

Two chromenols were isolated as minor compounds from *Desmarestia menziesii* collected from the Antarctic King George Island, one bearing a hydroxy group at C-13′ (99) and the other a carboxy group at C-13′ (100). The latter is a structural isomer of δ-sargachromenol (51) (see below) and shares structural similarity with garcinoic acid (30).^122,123^ Again, no optical activity was found for the two chromenes suggesting an epimeric center at C-2. However, the authors suggested a non-enzymatic ring closure within the living algae since no corresponding 1,4-benzoquinone was found as a potential precursor.

A C-15′-aldehyde-bearing chromenol (101) with anti-leishmanial activity was found as minor compound in the Southern Australian brown alga *Sargassum paradoxum* and the Japanese algae *Sargassum yamadae*.^124^

δ-Sargachromenol is one the most investigated meroditerpenoid obtained from marine organisms. As mentioned above, its unique structure resembles a δ-chromenol ring system with an unsaturated side chain containing a carboxy group at C-15′. δ-Sargachromenol is widely distributed in *Sargassum* species such as *Sargassum sagamianum*,^73,125,126^*Sargassum serratifolium*,^54,127,128^*Sargassum micracanthum*,^129^*Sargassum horneri*,^130^*Sargassum macrocarpum*,^131,132^ and *Sargassum fallax*.^133^ The latter species contains δ-sargachromenol as high as 0.13% of the dry weight. It has also been isolated from *Myagropsis myagroides* (Sargassaceae),^134^ from the tunicate *Botryllus tuberatus*^135^ and other algae.^134^

Kusumi *et al.* claimed 51 to be an artefact that is produced from sargaquinoic acid during the clean-up procedure. Although there is an asymmetric carbon center at C-2, the authors found no optical rotation. Literature data on the stereochemistry of sargachromenol are inconsistent. δ-Sargachromenol isolated from plant species showed optical rotation with an [*α*]_D_ of +5°,^53^ whereas δ-sargachromenol isolated from *Sargassum fallax* showed an [*α*]_D_ of -23.7°.^133^ Choi *et al.* isolated a racemic mixture from *Botryllus tuberatus* and separated δ-sargachromenol stereoisomers by chiral HPLC coupled with circular dichroism spectroscopy. They determined the absolute configuration for *R*-sargachromenol with an [*α*]_D_ of -68° and *S*-sargachromenol with an [*α*]_D_ of +88°.^135^ It is yet not clear whether sargachromenol should be considered as an artefact of the work-up procedure or as a natural product.^136^

Sargachromenol received attention in drug research since it has inhibitory activity against enzymes related to Alzheimer's disease, strong anti-inflammatory activity and anti-hyperproliferative properties in skin cells (Tables 1–3). Several patents are pending on the use of sargachromenol as drug candidate.^137^

Choi *et al.* found acetylcholinesterase- and butyrylcholinesterase-inhibitory activity with IC_50_ values of 32.7 and 7.3 μM, respectively.^125^ Recently, Seong *et al.* repeated the enzyme assays and determined slightly higher IC_50_ values (97.3 and 9.4 μM, respectively) for these enzymes. In addition, the authors found that 51 is a non-peptidic, noncompetitive inhibitor of β-site amyloid precursor protein-cleaving enzyme 1 (BACE1) with an IC_50_ value of 7.0 μM and a *K*_*i*_ value of 2.9 μM.^127^ Molecular docking experiments revealed that sargachromenol interacts with the allosteric side of BACE1.^127^ In line with these results, sargachromenol promotes neurite outgrowth and survival of rat PC12D pheochromocytoma cells *via* activating phosphatidylinositol-3 kinase.^131^ Based on its lipid-solubility and low molecular weight (<500 Dalton), sargachromenol should be able to cross the blood brain barrier, making δ-sargachromenol an interesting drug candidate for treating Alzheimer's disease and other neurodegenerative diseases.

Similar to δ-garcinoic acid, sargachromenol is a potent anti-inflammatory compound that prevented TPA-induced ear edema in mice with an IC_50_ value of 0.36 mg per ear.^53^ In addition, δ-sargachromenol inhibits lipoxygenase (LOX) (76% at 100 ppm) and cyclooxygenase (COX)-1 and -2 (98% and 84% at 100 ppm; Table 1).^52^

Sargachromenol inhibited LPS-induced inflammation markers in murine RAW 264.7 macrophages. Production of PGE_2_ and nitric oxide was inhibited (IC_50_ values of 30.2 and 82 μM, respectively) accompanied by a reduced protein expression of iNOS and COX-2.^129^ Kim *et al.* reported the inhibition of nitric oxide formation in LPS-stimulated murine microglial BV-2 cells with an EC_50_ value of 1.14 μg ml^−1^ (2.7 μM). These effects are accompanied by a suppression of the release of TNF-α, IL-1β, and IL-6.^134^ Several markers of vascular inflammation were also decreased in primary endothelial cells by δ-sargachromenol, namely TNF-α induced ICAM-1 and VCAM-1 expression, adhesion of monocytes to HUVEC and decreased production of monocyte chemoattractant protein-1 and matrix metalloproteinase-9 (MMP-9).^128^ Both epimers of sargachromenol bind to human farnesoid X receptor and inhibit its transactivation (IC_50_ values of 9.0 μM (*R-*epimer) and 17.0 μM (*S*-epimer), respectively). It is known that farnesoid X receptor agonists decrease plasma triacylglycerides and increase HDL cholesterol by regulating the expression of apolipoprotein C-I and C-IV.^135^ Summarizing the evidence (also from plant species), δ-sargachromenol (51) clearly is a candidate for an anti-atherogenic drug.

Sargachromenol has also been suggested as a drug for skin health, since it induced apoptosis in hyperproliferative human keratinocyte HaCaT cells and suppressed MMP-1, -2 and -9.^126,130^ Finally, insecticidal activity was found against the larvae of *Spodoptera frugiperda* with a LD_50_ value of 2.94 μg ml^−1^.^136^

**Fig. 13:**
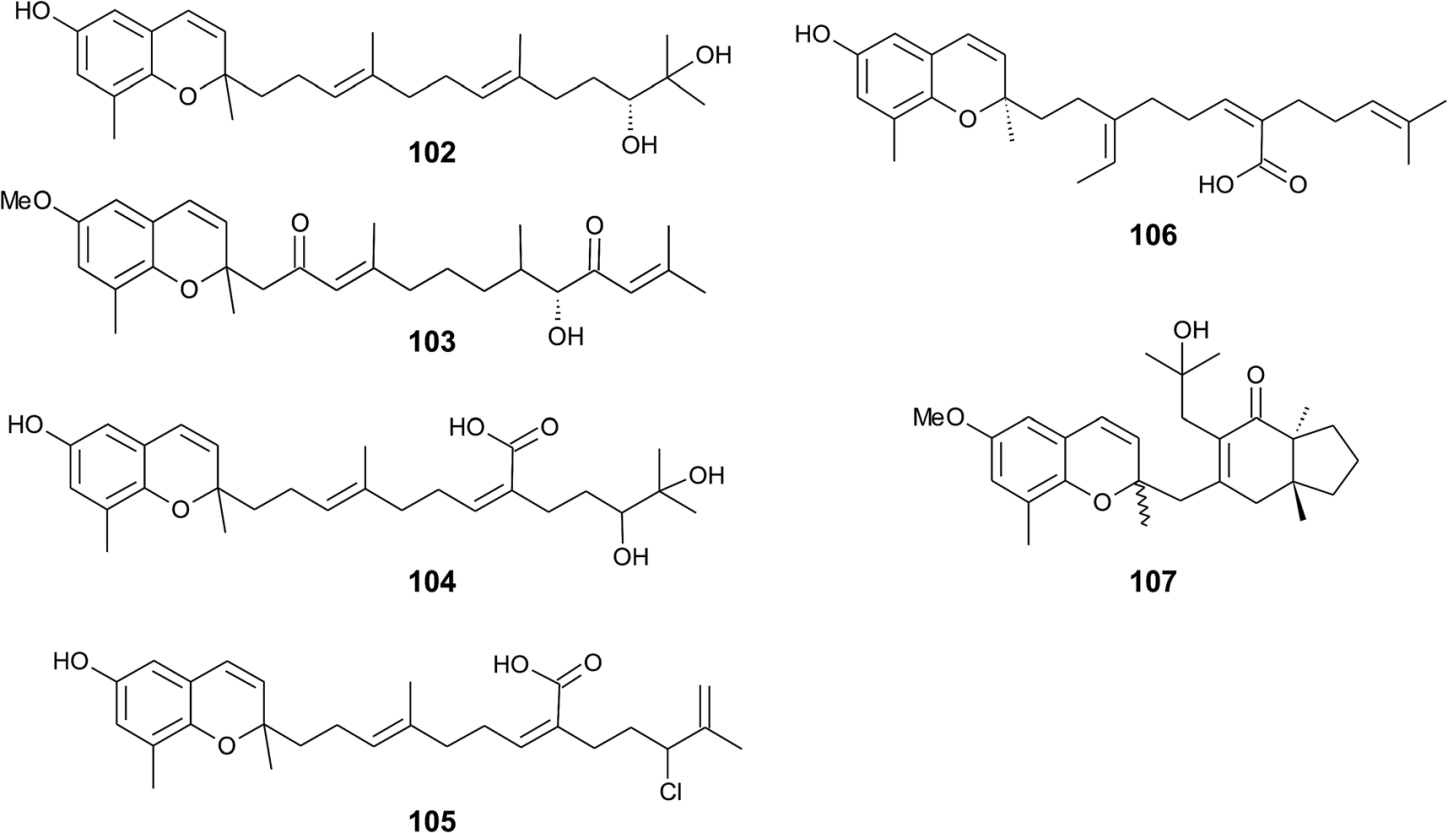
Structures of sargachromenols (102 to 107) from *Sargassum* species.

Iwashima *et al.* synthesized a dihydroxylation product of sargachromenol from the corresponding plastoquinone precursor that had been isolated from *Sargassum micracanthum*.^58^ To the best of our knowledge, 11′-,12′-dihydroxy-sargachromenol (102) (Fig. 13) has never been isolated as a natural product from algae before. However, the compound has been investigated for its anti-viral activity against human cytomegalovirus,^58,60^ its anti-ulcer activity in ethanol-induced gastric lesions in rats,^138^ and inhibitory activity in osteoclastogenesis (bone resorption), thus suggesting that this compound is an interesting pharmacological lead structure.^59^

Multiple biosynthetic oxidation steps lead to a highly oxidized chromane (103), which was found in *Halidrys siliquosa* (Sargassaceae) from the French Atlantic coast.^139^ Two keto groups at positions C-2′ and C-10′ and a hydroxyl group at C-9′ with *R*-configuration could be assigned by two-dimensional NMR spectroscopy.

Natural derivatives of δ-sargachromenol 52 have been isolated from different algae species. Besides δ-sargachromenol, sargothunbergol A (104), a sargachromenol with two additional hydroxyl groups at C-11′ and C-12′, was isolated as a minor compound from *Sargassum thunbergii*, collected from the shore of the Korean Youngdo Island.^66,140^ Fallachromenoic acid (105) from the Australian alga *Sargassum fallax* is an interesting variation as it bears a chlorine atom at C-11′ and a terminal double bond (Fig. 13).^133,141^ Fallachromenoic acid was isolated in 0.06% yield (dry mass) and exhibited moderate anti-tumor activity in the murine leukemia P388 cell assay (IC_50_ value of 29 μM).

Along with the sargachromanols described by Jang *et al.*,^76^ mojabanchromanol (106) has been isolated from *Sargassum siliquastrum*,^142^ showing a rearranged carbon skeleton at C-3′ of the side chain.

Only two chromenols with cyclic side chain modifications were found in the literature. A 3,4-unsaturated analogue of bifurcarenone chromane (107) was identified in *Cystoseira amentacea* collected from the French Riviera and an unsaturated analogue of compound 107 from *Cystoseira baccata*.^143^

#### 3.3.2 Phytoplankton (green algae, cyanobacteria, phytoflagellates)

Green algae, cyanobacteria, phytophlagellates and other microalgae are members of the phytoplankton that produces α-tocopherol, which is essential for higher marine organisms. In addition, spirulina (*Arthrospira platensis*) is nowadays used in human nutrition as a food supplement. A screening of microalgae for α-tocopherol content reported various amounts starting from 58.2 μg g^−1^ (dry weight) for *Isochrysis galbana* up to 669 μg g^−1^ (dry weight) for *Chlorella stigmatophora*.^144^ The amount of α-tocopherol in spirulina varied between 5 and 14 μg g^−1^ dried spirulina.^145^ As a subject of culture conditions, the phytoflagellate *Euglena gracilis Z* produces high amounts of α-tocopherol and -tocotrienol (7 mg g^−1^ and 2.6 mg g^−1^ dry weight, respectively).^146^

**Fig. 14:**
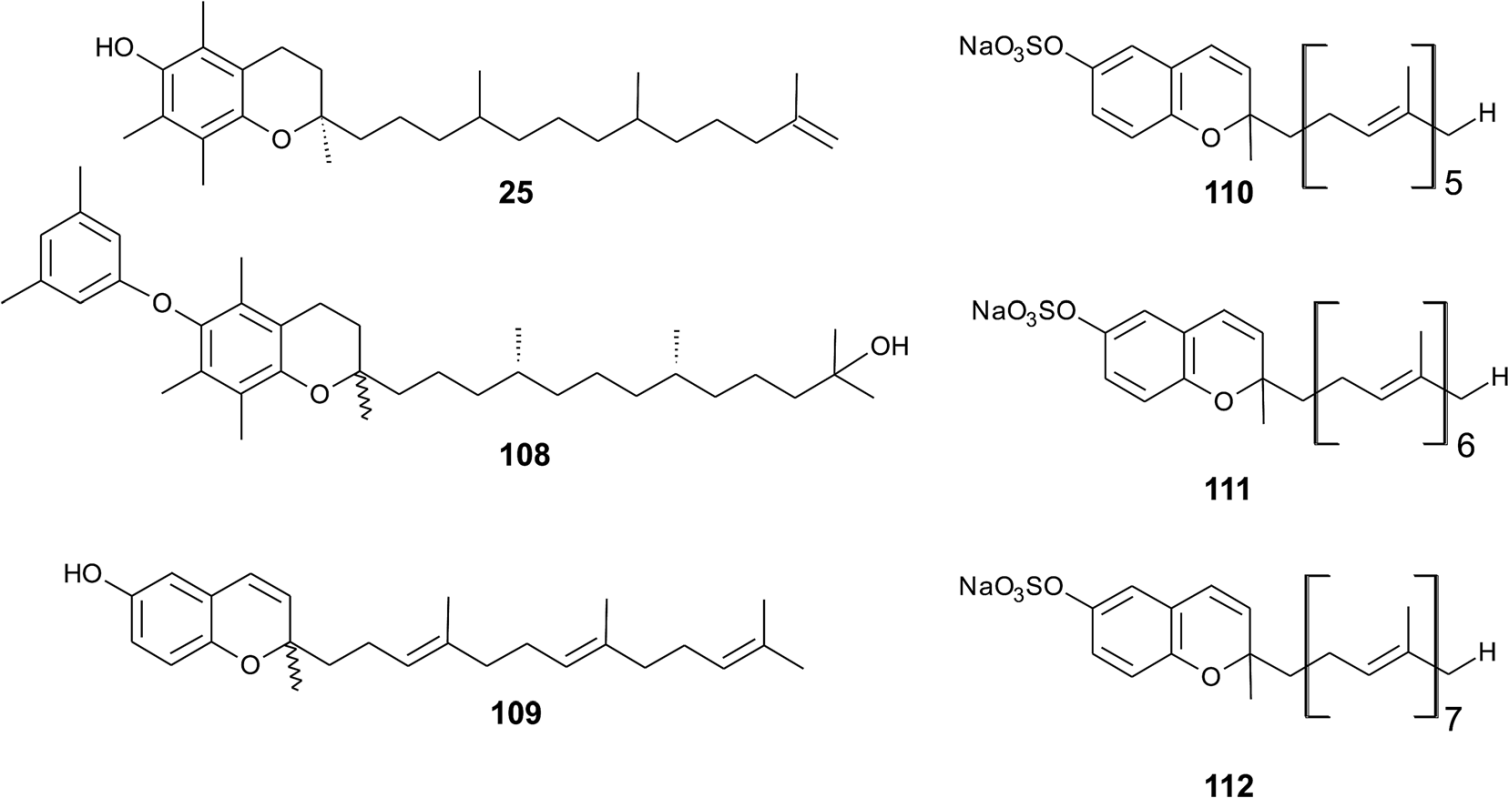
Structures of meroditerpenoids (25) and (108) from phytoplankton and sarcochromenols (110 to 112) from *Sarcotragus spinulosus*.

As reported by Yamamoto *et al.*, cold water fish contains a substantial amount of marine-derived tocopherol (25) (MDT), an α-tocomonoenol with a terminal double bond between C-12′ and C-13′ (Fig. 14).^147^ Since tocochromanols are only synthesized by photoactive organisms, the authors suggested a dietary source for MDT in fish. In fact, phytoplankton contains up to 21% (of total tocopherol) MDT. Also Antarctic krill (*Euphasia superba*) contains up to 8% (of total tocopherols) MDT.^148^ The biosynthesis of 25 is largely unknown; however, the authors suggested that the terminal double bond is introduced by side chain desaturation of α-tocopherol, similar to that of fatty acids.

Recently, an unusual α-tocopheroid, α-tocoxylenoxy (108), containing a 3,5-dimethylphenoxyl moiety was isolated from the seaweed *Caulerpa racemosa*,^149^ taxonomically also belonging to the green algae (*Chlorophyta*).

#### 3.3.3 Invertebrates (sponges, Ascidiacea, soft corals)

Sponges or *Porifera* comprise a group of more than 9000 species. In the last decades, sponges have attracted scientists to investigate the diversity of natural products and their properties.^150^ From a chemotaxonomic point of view, it is worth to note that some of the structures found in sponges that contain a chromene core lacking the typical methylation pattern. These sarcochromenols and the group of strongylophorines possess the highest structural variability in the organisms presented in this review.

A hypothetic biosynthetic precursor of the chromene structure was found in the Western Australian sponge *Fasciospongia* species (order of *Dictyoceratida*, family of *Thorectidae*).^151^ Fascioquinol F (109) is a demethylated 3-4-dehydro-tocotrienol that might undergo cyclization to form complex ring systems in analogy to taondiols (see the section on Brown algae). The structure is similar to sargaol (95), but lacks the methyl group at C-8 (Fig. 14). Fascioquinol F revealed moderate antibacterial activity against *Staphylococcus aureus* and *Bacillus subtilis* (IC_50_ values of 13 and 30 μM, respectively).

Sarcochromenols A (110), B (111) and C (112) are a group of long-chain tocochromenols with five, six and seven isoprene units, respectively (Fig. 14). They were isolated from the Pacific Ocean sponge *Sarcotragus spinulosus* (Schmidt) (family of Thorectidae) and showed Na^+^/K^+^-ATPase inhibitory activity similar to that of the sargachromanols D, F, H and L (IC_50_ value for sarcochromenol A of 1.6 μM).^78,152^ The compounds have also been isolated from the Indian sponge *Ircinia fasciculate* (Spongillidae).^153^ In addition, an un-sulfated form of sarcachromenol B was isolated in 0.1% yield.

**Fig. 15:**
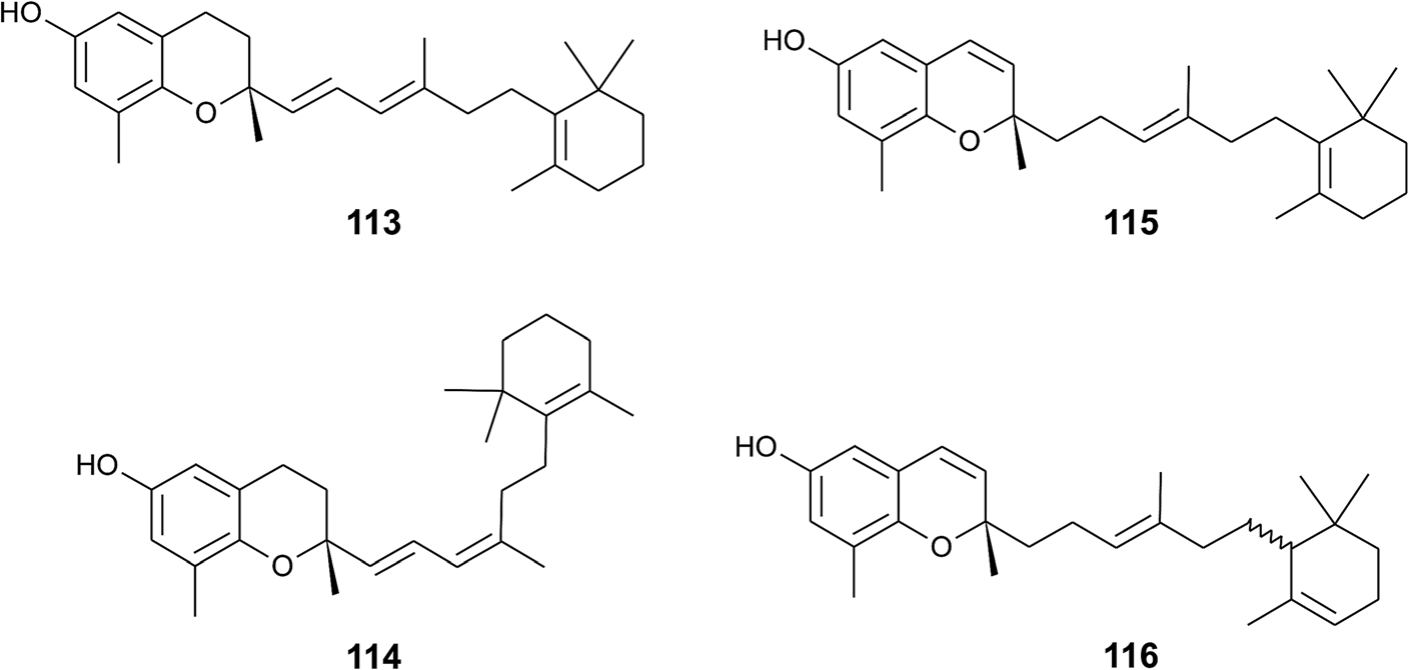
Structures of chromarols (113 to 116) from *Psammocinia* species.

A screening for selective human 15-LOX inhibitors from an extract of the Papua New Guinean sponge *Psammocinia* (order of Dictyoceratida, family of Irciniidae) revealed chromarols A to D (113 to 116; Fig. 15).^154^ The IC_50_ values for chromarols A to D were 0.6, 4.0, 0.7 and 1.1 μM, respectively. The authors found high selectivity since the IC_50_ values for 12-LOX were above 100 μM. The biosynthesis of the cyclohexene ring system in the side chain of chromarols presumably derives from an acid-catalyzed cyclization.

**Fig. 16:**
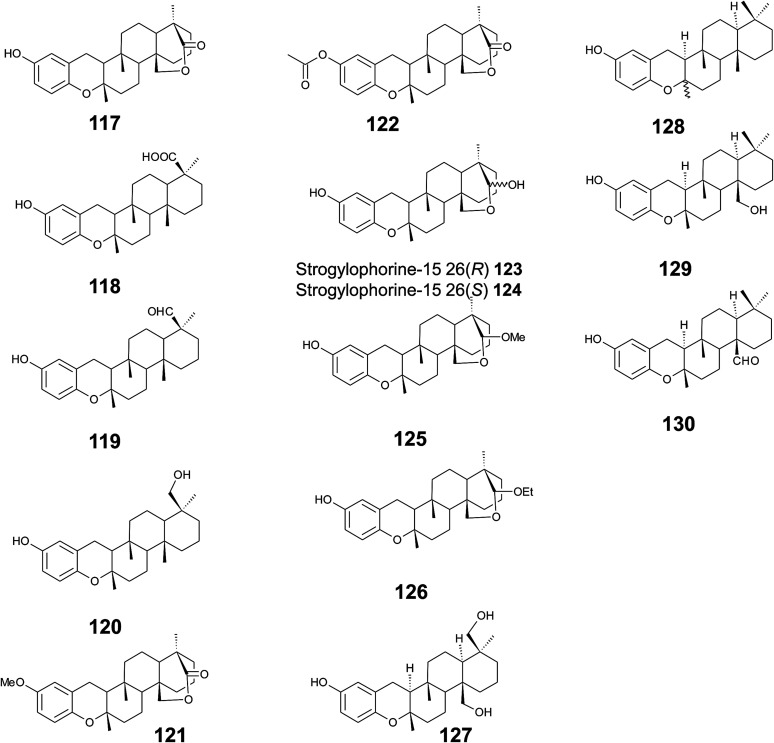
Structures of strongylophorines (117 to 130) from *Strongylophora* species.

Several sponges produce a group of eight polycyclic strongylophorines that resemble taondiol structural motives (Fig. 16). They contain a demethylated aromatic ring and modifications at the methyl groups at C-13′ and/or C-15′. They were discovered by Braekman *et al.* because of their ichthyotoxic activity.^155^ The biosynthesis follows that of taondiol and is an enzyme-catalyzed cyclization cascade (see Fig. 10). Strongylophorines 2 (117), 3 (118), 4 (119), and 5 (120) were isolated from *Strongylophora durissima* from Maricabiin Island, Philippines,^156^ and a different, as yet undescribed *Strongylophora* species from Ilocos Sur, Philippines.^157^ These molecules contain a cyclic lactone, a carboxy, an aldehyde or a hydroxyl group moiety at C-13′, respectively. Strongylophorine 3, bearing a terminal carboxy group, was isolated with 0.1% yield (dry weight).^156^ Furthermore, the known strongylophorines 3, 9 (121) and 11 (122) were isolated from a Taiwanese species of *Strongylophora durissima*. The 6-methoxy (121) and 6-acetyl (122) derivatives are structurally related to strongylophorine 2, which contains a cyclic lactone moiety.^158^ Liu *et al.* isolated the strongylophorines 15 (26*R*) (123) and 16 (26*S*) (124), respectively, from the Okinawan sponge *Strongylophora strongylata* as epimers at the hemiacetal carbon.^159^ Biosynthetic *O*-methylation and *O*-ethylation gave the acetals 26-*O*-methoxystrongylophorine 16 (125) and 26-*O*-ethoxystrongylophorine 16 (126), respectively.^160,161^ Noda *et al.* found a mixture of strongylophorines 15 and 16 to be strong inhibitors of the proteasome with IC_50_ values of 3.6 μM.^160^ The same study compared the proteasome-inhibitory activity of structurally related strongylophorines and found the following order: hemiacetal > acetal ∼ carboxy > lactone > no modification.

On their search for inhibitors of protein tyrosine phosphatase 1B, an enzyme that plays a crucial role in the regulation of insulin and leptin signalling, Lee *et al.* found inhibitory activity for 125, 117, 118, 123, and strongylophorine 17 (127) with IC_50_ values of 8.5, 24.4, 9.0, 11.9, and 14.8 μM, respectively.^161^ Strongylophorines 2 and 3 also inhibited hypoxia-inducible factor-1-dependent luciferase expression in engineered U251-HRE glioma cells with EC_50_ values of 8 and 13 μM.^162^ Strongylophorines 22 (128), 23 (129), 24 (130), and 17 (127) were isolated from the Okinawan sponge *Petrosia corticata* and displayed moderate cytotoxic activity against uman cervical carcinoma epithelial (HeLa) cells (Table 2).^163^ All strongylophorines exhibited ichthyotoxic, insecticidal, anti-bacterial, fungicidal, and cytotoxic properties. Strongylophorine 22 and fascioquinol D are epimers at C-2 and were isolated from *Fasciospongia* sp.^151^ The latter compounds displayed anti-microbial activity against *Staphylococcus aureus* and *Bacillus subtilis* with IC_50_ values of 25 and 2.3 μM (for strongylophorine 22) and 7.8 and 2.8 μM (for fascioquinol D), respectively.

Recently, Yu *et al.* presented the first semi-synthesis of strongylophorine 2 starting from isocupressic acid.^164^

#### 3.3.4 Ascidiacea/tunicates

**Fig. 17:**
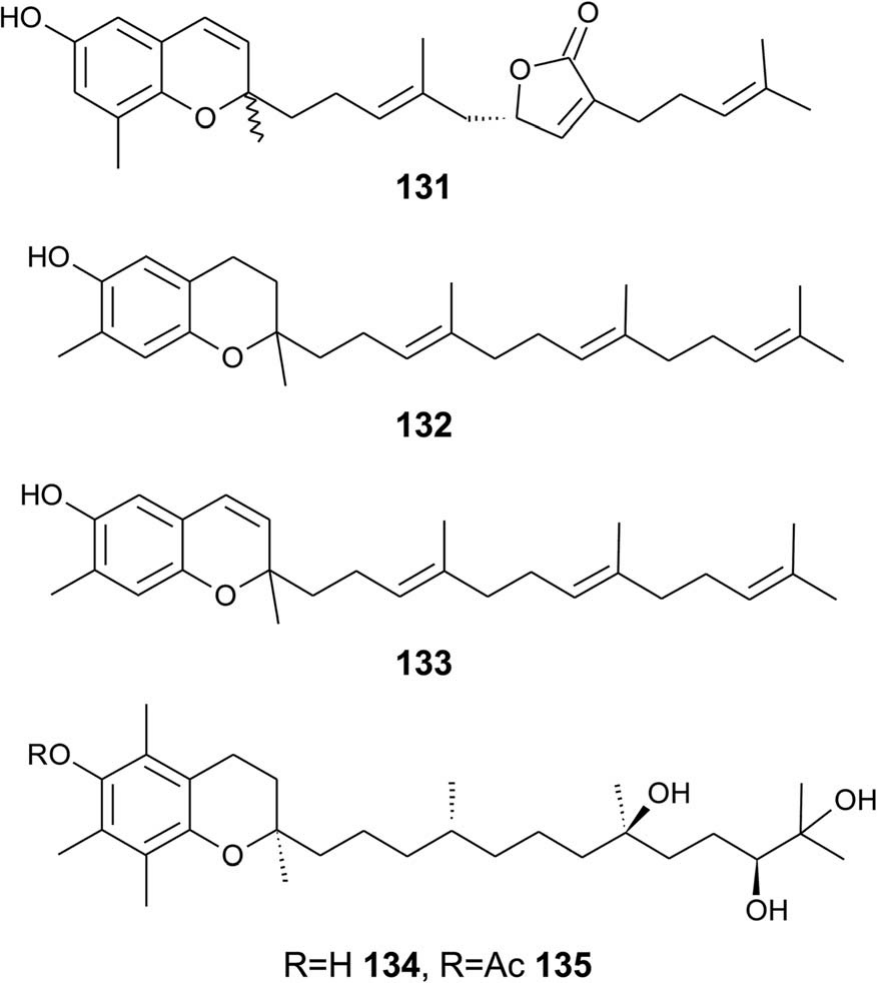
Structures of tuberatolide (131) from the tunicate *Botryllus tuberatus* and meroditerpenoids (132 to 135) from soft corals.

Ascidians, tunicates or sea squirts belong to a group of more than 3000 species, most of them not investigated in terms of bioactive metabolites. In a recent review, Palanisamy *et al.* described almost 600 chemical structures found in tunicates.^165^ Here, we describe meroditerpenes, such as an epimeric mixture of *R*- and *S*-sargachromenol and two epimeric chromenes called tuberatolide B and 2′-*epi*-tuberatolide B (131), obtained from the tunicate *Botryllus tuberatus*.^135^ Tuberatolide B contains a γ-lactone moiety within the side chain that presumably derives from a C-15′-carboxy, C-6′-hydroxy-precursor (Fig. 17). Both tuberatolides were strong farnesoid X receptor agonists with IC_50_ values of 1.5 and 2.5 μM, respectively.^135^

#### 3.3.5 Soft corals

Soft corals (Alcyonacea) belong to the class of Anthozoa and compromise approximately 800 species living mostly in warm seawater. In recent years, the number of new metabolites discovered from soft corals was estimated to represent 22% of the total new marine natural products.^166^ Many metabolites showed anti-tumor, anti-viral, anti-fouling and anti-inflammatory activities (reviewed in (ref. 167)).

Bowden *et al.* isolated tocotrichromenol (132), an isomer of sargaol (95), and its dihydro derivative (133) from an unknown Australian *Nephthea* species.^168^ The precursor quinone was also isolated, but did not convert into the chromenol under the work-up conditions; however, no optical activity was found at C-2.

Two α-tocopherol derivatives with three hydroxyl groups at C-8′, C-11′, and C-12′, respectively, were isolated from *Lobophytum crissum* (Fig. 17). Crassumtocopherol A (134) (R=H) and B (135) (R=acetyl) showed moderate cytotoxicity against murine P-388 leukemia cells with IC_50_ values of 6.7 and 5.2 μM, respectively.^169^ Compound 135 also showed cytotoxicity against the human colon adenoma cell line HT-29 with an IC_50_ value of 7.5 μM.

## 4. Merosesquiterpenes

### 4.1 Plants

The biosynthesis of chroma(e)nols with sesqui-, mono- and hemi-terpene moieties within the plant kingdom is only poorly understood. These molecules most likely derive from homogentisate condensed with farnesyl-, geranyl- and isoprenyldiphosphates, respectively.

**Fig. 18:**
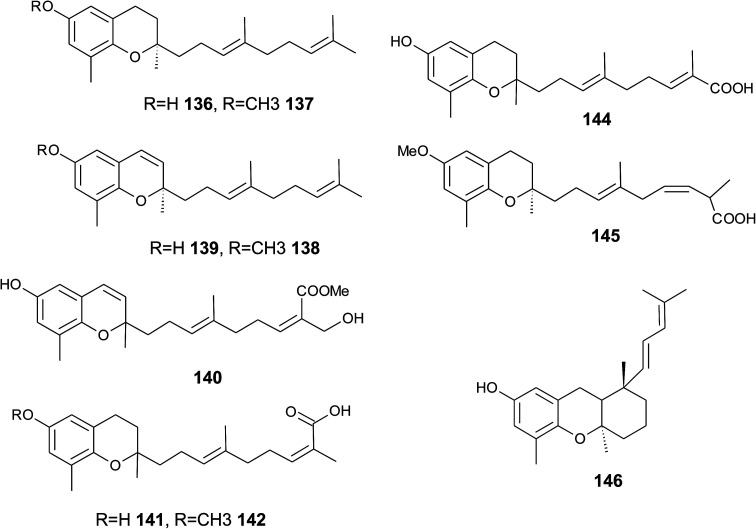
Structures of plant sesquiterpenes (136 to 146).

Oligandrol (136), a sesquiterpenechromane with an unsaturated side chain, was isolated together with methoxy-oligandrol (137) from the bark of the Australian tree *Beilschmiedia oligandra* (Lauraceae),^170^ and from the leaves of the genus *Pseuduvaria indochinensis* Merr, an Annonaceae variety from the Yunnan province in China (Fig. 18).^171^ Cytotoxic assessment revealed no activity against promyelocytic HL-60 leukemia cells and human SMMC-7721 hepatocarcinoma cells.^171^

The methoxy-derivative of dehydrooligandrol (138) was obtained from the root of *Beilschmiedia erythrophloia*^172^ and the free dehydrooligandrol (139) from the leaves of *Seseli farreynii* (Umbelliferae). However, the latter was suggested to be an artefact from the work-up procedure.^173^ Zhao *et al.* isolated a dehydrooligandrol with a terminal (*Z*)-carboxy and a 13′-hydroxy group, respectively, which the authors named pseudindochin (140).^171^

Polycerasoidol (141), an oligandrol derivative with a terminal (*Z*)-carboxy-group and its 6-methoxy-derivative polycerasoidin (142) were found in the stem bark of the Papua New Guinean *Polyalthia cerasoides*.^174^ Later, the methyl ester of polycerasoidin (143) and the *E*-isomer of polycerasoidol, termed isopolycerasoidol (144), were identified in the same species.^175^ Polycerasoidin was isolated at 0.13% yield (dry weight). Polyalthidin (145), a structural isomer of polycerasoidin with a double bond shift from C-7′–C-8′ to C-6′–C-7′ was isolated from *P. cerasoides* at a yield of 0.09% (Fig. 18).^176^ Polycerasoidol, polycerasoidin and polyalthidin were found to be inhibitors of the mitochondrial electron transfer chain that block NADPH oxidase activity with IC_50_ values of 37, 11 and 4.4 μM, respectively.^176^

Riccardiphenol C (146), a sesquiterpene from the New Zealand liverwort *Riccardia crassa*, is an example of a chromanol that undergoes intramolecular cyclization to form a condensed ring system. Purification of the crude extract yielded riccardiphenol C in 4 mg g^−1^ of dry liverwort (Fig. 18). The compound showed cytotoxicity against African green monkey BSC-1 kidney cells and inhibited the growth of *Bacillus subtilis*.^177^

### 4.2 Fungi

**Fig. 19:**
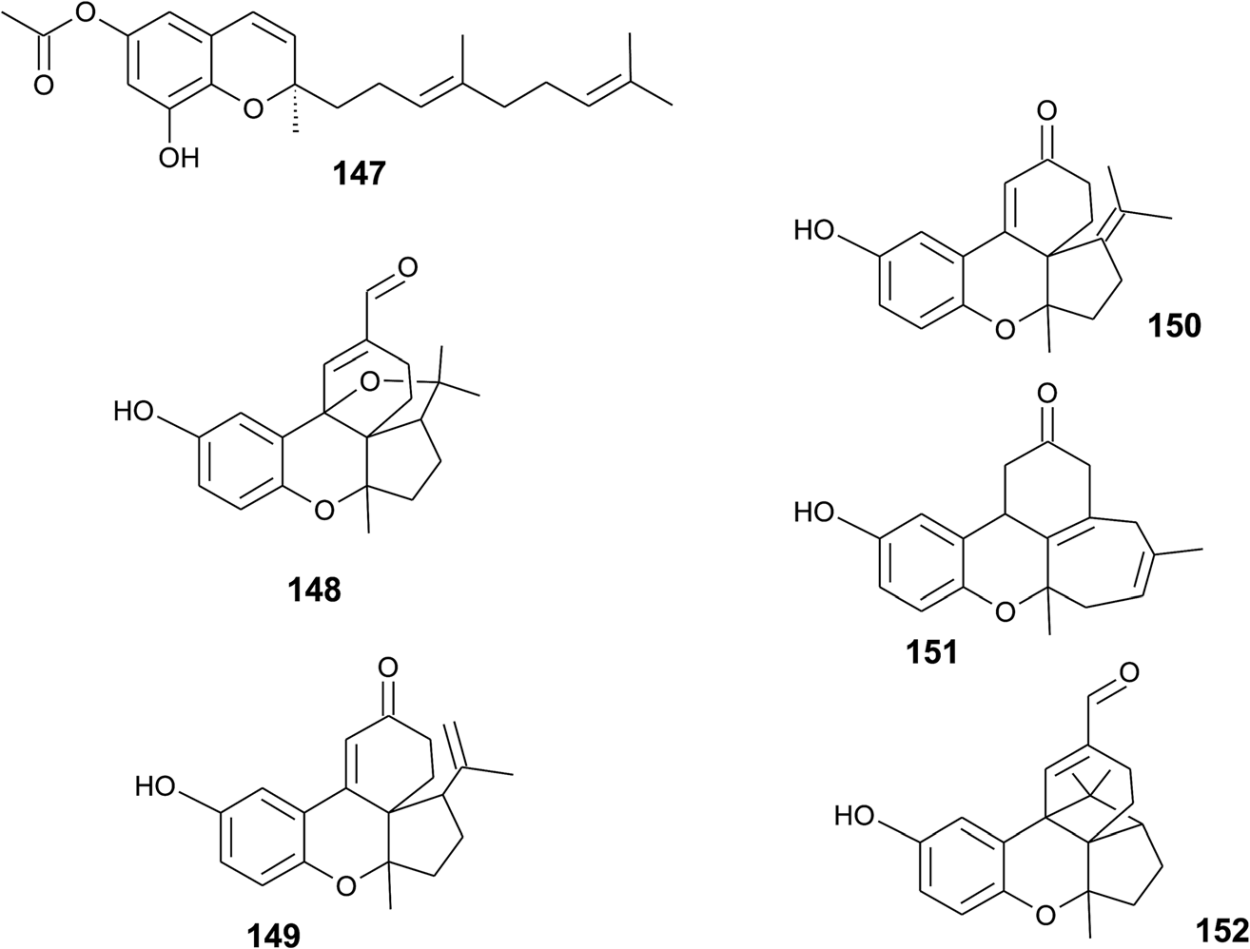
Structures of sesquiterpenes (147 to 152) from fungi species.

A sesquiterpene chromene (147) with a truncated tocochromene-like structure was isolated from *Chroogomphus rutilus*.^178^ The mushroom is also known as brown simecap and lives ectomycorrhizally with *Pinus* species. The compound shows *R*-configuration at the chiral center C-2 (Fig. 19).

Polycyclic sesquiterpenes were isolated from the fruiting bodies of the tropical rot fungus *Ganoderma cochlear*.^179^ Ganocin A to C (148–150) possess a spiro[4,5]decane ring, and ganocin D (151) has an eight-membered ring system. As a biosynthetic key step, the authors suggest a Diels–Alder reaction of fornicin C to build up the polycycles. In the same fungus, Dou *et al.* found cochlearol B (152) with an unusual 4/5/6/6/6 polycyclic ring system (Fig. 19). The compound is a strong inhibitor of the TGF-β/Smad signaling pathway.^180^

### 4.3 Marine organisms

#### 4.3.1 Brown algae

**Fig. 20:**
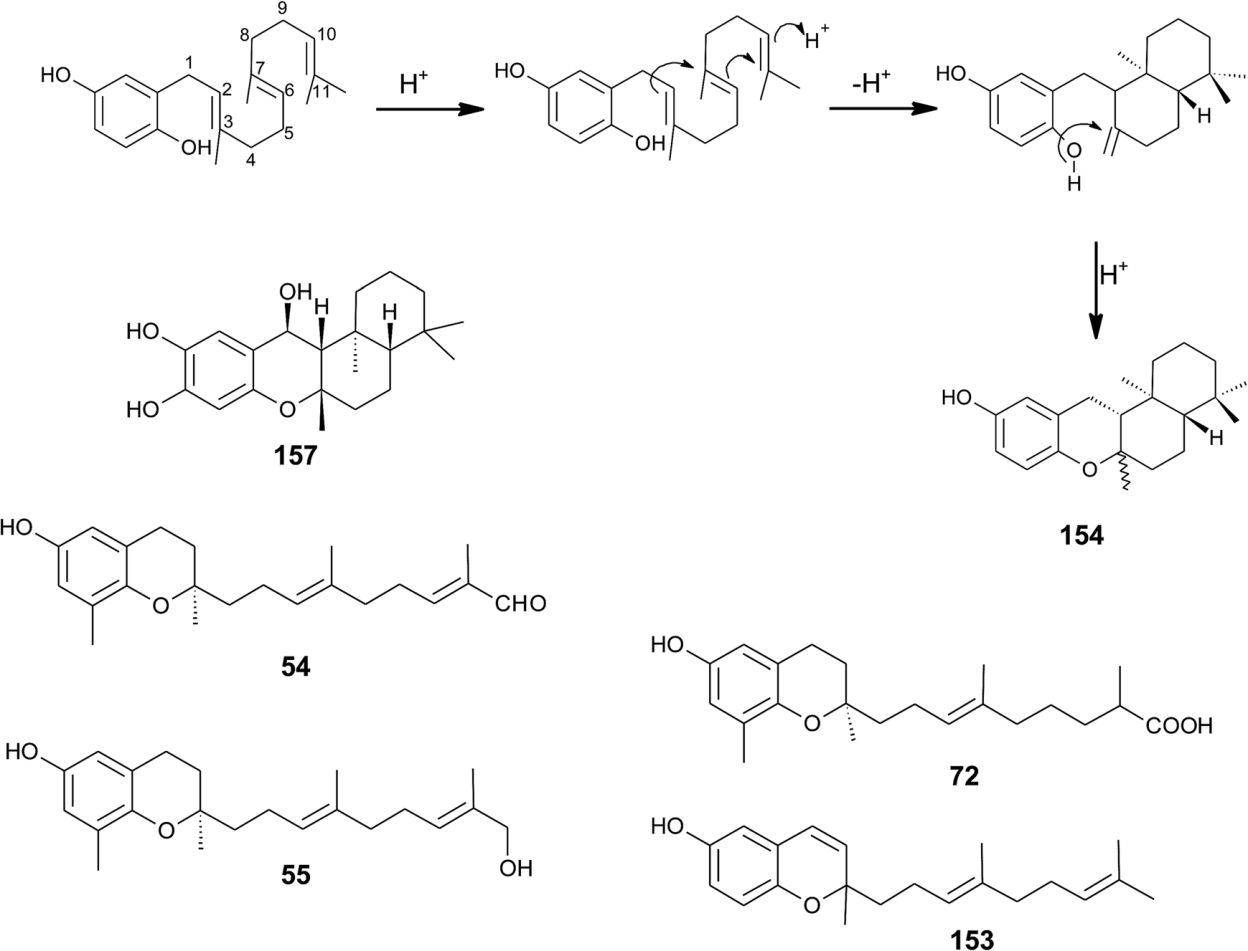
Scheme of multiple acid-catalyzed cyclizations of farnesyl hydroquinone towards chromazoranol (154) as suggested by Kurata *et al.*^186^ Structures of cyclic (157) and linear sesquiterpenoids (54, 55, 72 and 153).

Jang *et al.* described a series of meroditerpenes, the sargachromanols (A to P), from *Sargassum siliquastrum*. Sargachromanols A, B and S possess a sesquiterpene skeleton with a terminal aldehyde- (sargachromanol A) (54), alcohol- (sargachromanol B) (55) or carboxy-function (sargachromanol S) (72), respectively.^76–78^ All compounds were assigned *R*-configuration at C-2 (Fig. 20).

Dictyochromenol (153) and its cyclization product chromazonarol (154) were both isolated from the Japanese brown alga *Dictyopteris undulata*.^181–183^ Dictyochromenol is comprised of a demethylated chromanol ring which is attached to an unsaturated sesquiterpene moiety. A chemical synthesis route of dictyochromenol was described by Aoki *et al.*^184,185^ Kurata *et al.* suggested an acid-catalyzed cyclization of farnesyl hydroquinone towards zonarol (1,4-hydroquinone) followed by a second acid-catalyzed formation of the epimeric center at C-2 of chromazonarol (Fig. 20).^186^ Chromazonarol showed algicidal activity towards *Heterosigma* and *Chattonella* species.^182^

#### 4.3.2 Sponges

A structural isomer of chromazonarol, aureol (155), and its 5-chloro-derivative (156) were isolated from the Caribbean sponge *Smenospongia aurea*.^187,188^ It has been suggested that aureol results from a rearrangement of the drimane skeleton of chromazonarol. Aureol showed moderate cytotoxic activity against several cell lines, such as human adenocarcinomic A549 alveolar basal epithelial cells, human colon adenocarcinoma HT-29 cells, and murine EL4 lymphoma cells with IC_50_ values of 13.6, 14.9 and 31.5 μM, respectively.^189^ In 2002, Nakamura *et al.* presented a chemical synthesis of aureol.^190^ Besides aureol, 2-epichromazonarol was isolated (2.2% dry weight) from *Smenospongia aurea*.^187^ Recently, a structurally related meroterpenoid, puupehenol (157), with potent anti-microbial properties was isolated from the Hawaiian sponge *Dactylospongia* sp. (Fig. 20).^191^ The authors suggested that the well-known puupehenone may be a work-up artefact of the natural precursor puupehenol.

Two epimeric sesquiterpene chromenols, named cyclorenierin A and B (158), were found in *Haliclona* sp., an Indo-Pacific sponge from Vanuatu.^192^ The biosynthesis of the cyclohexenone ring system seems to follow that of walsurol (Fig. 6).

**Fig. 21:**
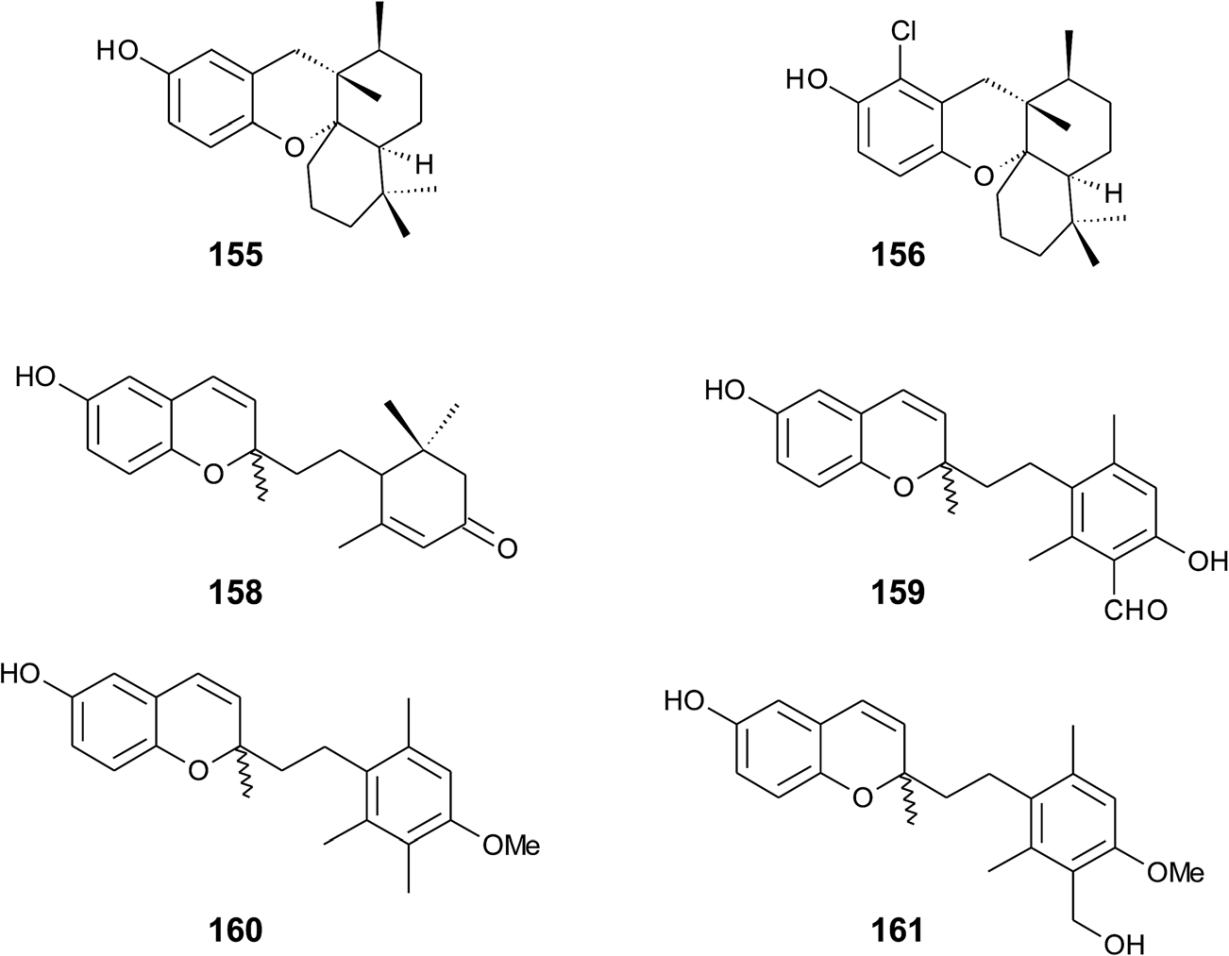
Structures of sesquiterpenes (155 to 161) from sponge species.

Panicein B2 (159) bears a chromene ring and an aromatic ring system in the side chain (Fig. 21). It was first isolated by Cimino *et al.* from *Haliclona panacea* and later from the Mediterranean sponge *Reniera fulva*.^193,194^ Panicein B2 was also found in *Reniera mucosa* along with panicein A2 (160) and F2 (161).^195^ It has been suggested that the aromatic group of the side chain is formed from cyclorenierin A/B by a 1,2-methyl migration and subsequent oxidation.^193^ All paniceins show racemic carbon centers at C-2 suggesting that these compounds may be artefacts from the work-up procedure.

**Fig. 22:**
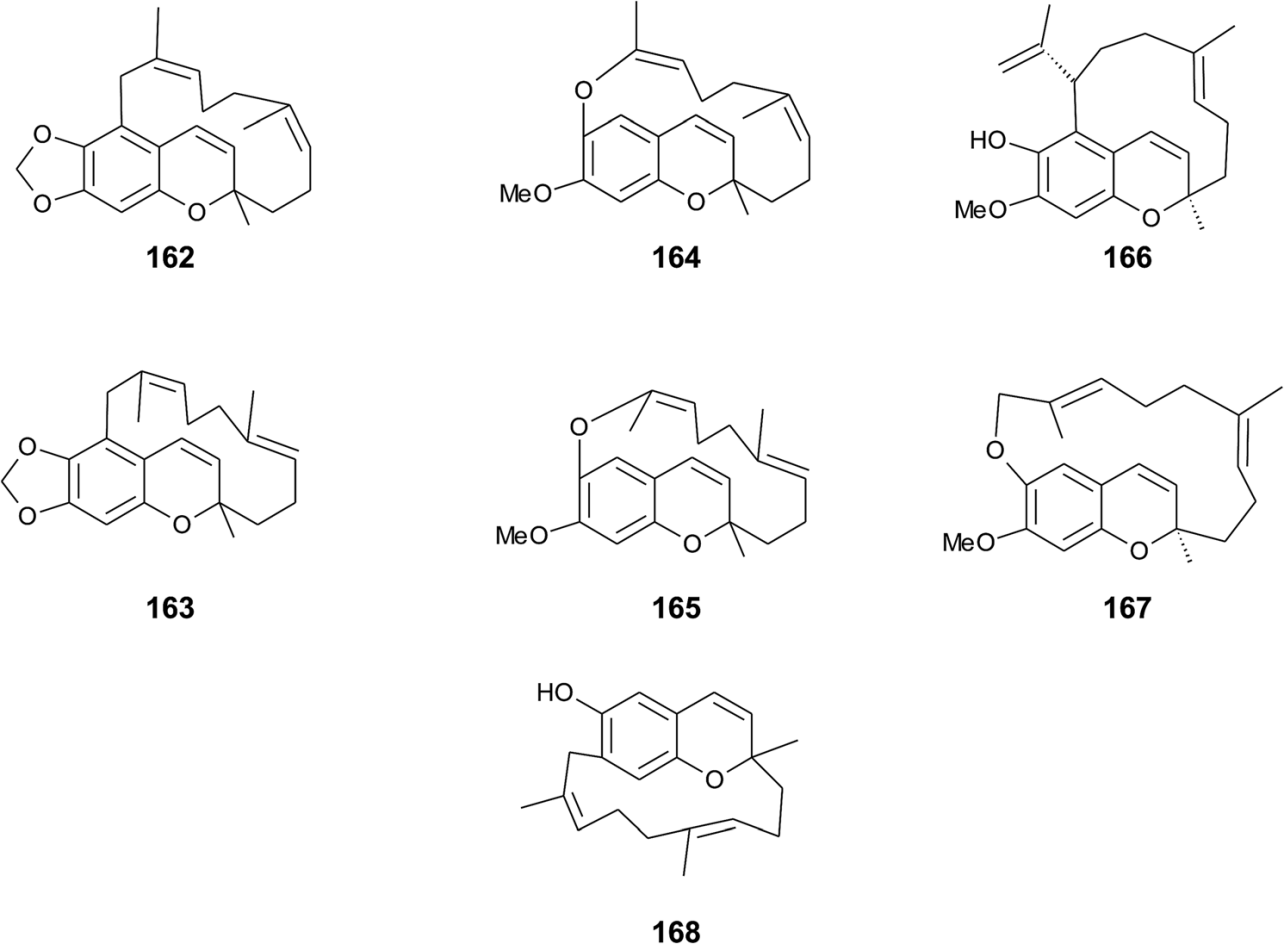
Structures of ansa chromane macrocycles (162 to 167) from *Smenospongia* species and (168) from tunicate.

Faulkner *et al.* isolated a series of unusual ansa chromene macrocycles from *Smenospongia* sp., a sponge from the Seychelles.^196^ Smenochromes A to D (162–165) were isolated with 0.26% yield (dry weight) for A and 0.037% for B, C and D, respectively (Fig. 22). The compounds showed no optical activity and thus occurred as racemic mixtures. The structurally related likonides A (166) and B (167) were isolated from the Kenyan sponge *Haytella* sp. with 0.06 and 0.04% yield.^197^ The biosynthesis of ansa chromenes presumably starts from a farnesylated hydroquinone followed by alkylation at C-5 of the activated hydroquinone ring or alternatively by *O*-alkylation of the terminal double bond.^197^

#### 4.3.3 Ascidiacea/tunicates

Longithorol E (168) was isolated as a minor metabolite from the Australian ascidian *Aplidium longithorax* (Fig. 22).^198^

#### 4.3.4 Molluscs

**Fig. 23:**
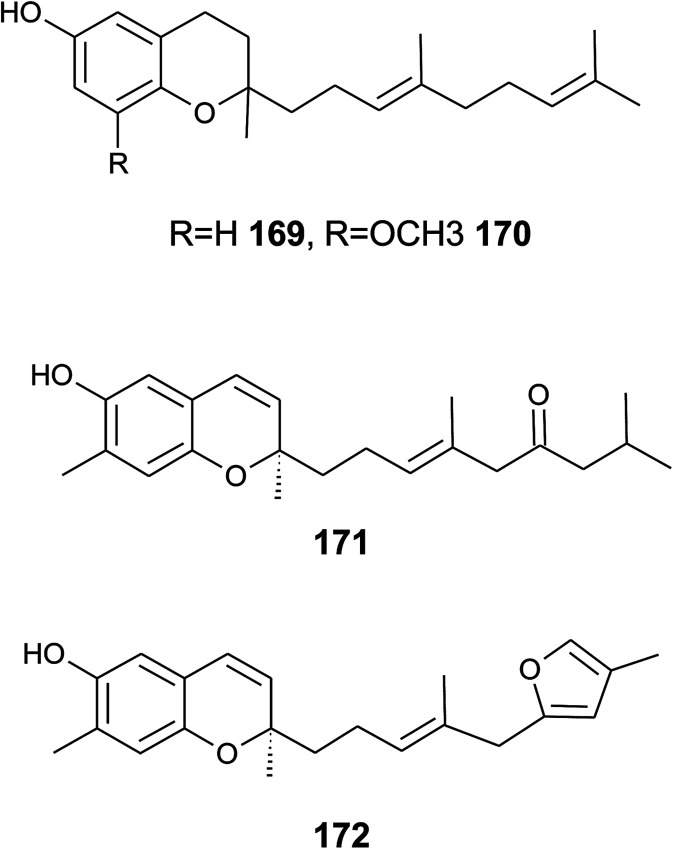
Structures of sesquiterpenes (169–171) from marine nudibranchs and soft coral (172).

There is emerging interest in the metabolites of marine nudibranchs. Since these animals completely lost their protective shell, the production or accumulation of toxins from their prey is used as defense systems.^199^ Two oligandrol-like structures (169) and (170) were isolated from *Cratena peregrine*, and a chromenol (171) with a C-6′ ketone moiety was found in the frilled nudibranch *Leminda millecra* that is only found in South Africa^200^ (Fig. 23).

#### 4.3.5 Soft corals

Although sesquiterpenes are widely distributed in soft corals, we only found sparce information on sesquiterpene chromanes and chromenes, respectively.^201^ Capillobenzopyranol (172) was isolated from the Australian soft coral *Sinularia capillosa* and showed moderate cytotoxicity against P-388 cells (ED_50_ values of 12.7 μM).^202^ Its quinone precursor has been isolated from *Sinularia lochmodes*.^203^ The compound with a terminal furanyl moiety showed *in vitro* anti-inflammatory activity against LPS-activation in murine RAW 264.7 macrophages. Protein expression of iNOS was inhibited by 36.7% at 10 μM concentration of 172 (Table 1), however expression of COX-2 was not affected.

## 5. Monoterpenes

Monoterpenes from plant origin have been used since ancient times to treat certain diseases, such as inflammation or cancer. De Sousa and colleagues summarized the anti-cancer and anti-inflammatory activities of monoterpenes in an outstanding recent review.^204^

### 5.1 Plants

The monoterpene cordiachromene A (173) was isolated from the heartwood of the tropical American tree *Cordia alliodora* (Boraginaceae) by Manners *et al.*^205^ The authors proposed geranyl benzoquinol as the biogenic precursor of the compound. Interestingly, the woods of *Cordia alliodora* are recognized for their durability in marine uses. Cordiachromene A was also isolated from the extract of different tunicates and was further tested for its bioactivity (see section on Tunicates (5.2)).

**Fig. 24:**
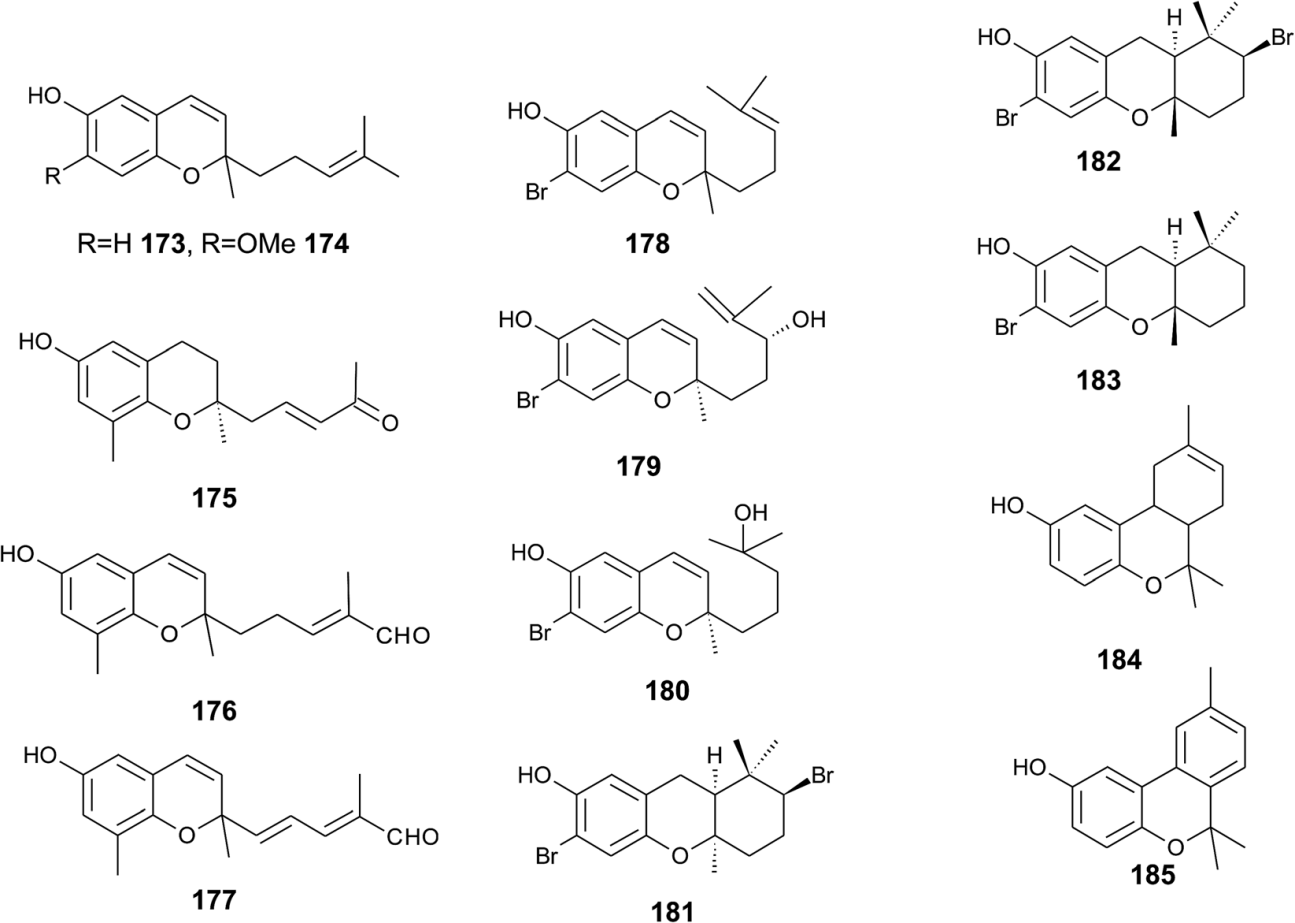
Structures of monoterpenes (173 to 185) from plants, marine algae and tunicates.

As part of the investigation of *Garcinia amplexicaulis* (see section on Diterpenes), a short-chain chromane (175) with a truncated C-9 carbon skeleton was found (Fig. 24).^48^

### 5.2 Marine organisms

#### 5.2.1 Brown algae

Next to the large number of di- and sesqui-terpenes, only a few monoterpenes have been described in the literature. Numata *et al.* isolated side chain truncated aldehydes and named them sargasal-I (176) and sargasal-II (177), respectively (Fig. 24).^71,120^

#### 5.2.2 Green algae

Cymopochromenol (178) was the first halogenated metabolite found in green algae. The 7-bromo-chromene was isolated from the Bermudan *Cymopolia barbata* as an optically inactive oil with 0.17% yield and also from Canary Island species with a yield of 0.02% dry weight.^181,206^ Later, Dorta *et al.* isolated two further chromenes, namely 3′- (179) and 4′-hydroxycymopochromenol (180), from the same source.^207^ Interestingly, both compounds showed optical activity with *R*-configuration at C-2. Two cyclic chromenes with two bromo atoms were isolated from *Cymopolia barbata* found in Puerto Rico.^208^ Cymobarbatol (181) and its epimer isocymobarbatol (182) showed anti-mutagenic activity. Debromo-isocymobarbatol (183) was isolated from *Cymopolia babata* (yield 0.2%, dry weight) collected at the Florida Keys and exhibited anti-feedant activity.^209^

#### 5.2.3 Ascidiacea/tunicates

Targatt *et al.* first reported the occurrence of cordiachromene A (173) in the marine ascidian *Aplidium constellatum* found around the Georgian coast.^210^ Later, cordiachromene A was isolated from *Aplidium antillense* from Guadeloupe, *Aplidium aff. densum* from Masirah Island (Oman), Japanese *Aplidium multiplicatum* and *Aplidium conicum*, respectively.^211–215^ Cordiachromene A showed anti-inflammatory activity *in vitro* and *in vivo*.^211,214,216^ The compound reduced carrageenan-induced rat paw edema with an IC_50_ of 18.9 μM and inhibited PGI_2_ synthesis in arachidonic acid-stimulated peritoneal rat macrophages (IC_50_ value of 8.2 μM).^211,216^ Sato *et al.* isolated cordiachromene A and 7-methoxy-cordiachromene A (174) from Japanese *Aplidium multiplicatum* and observed strong inhibitory activity against 15-LOX with IC_50_ values of 0.82 μM and 1.9 μM, respectively.^214^

Cordiachromene A showed anti-bacterial activity against methicillin resistant *Staphylococcus aureus* and *Streptococcus faecalis*,^212^ but weak activity against *Micrococcus luteus* (the minimum inhibitory concentration was 0.51 mmol L^−1^).^213^ Cytotoxic activity was found against a panel of cancer cell lines, such as murine leukemia P388 cells, human adenocarcinomic A549 alveolar basal epithelial cells, human colon adenocarcinoma HT-29 cells, and African green monkey CV-1 kidney fibroblasts, and drug-sensitive human leukemic lymphoblasts (IC_50_ value of 30 μM).^213,217^

So far, three cyclization products of cordiachromene A were found; conical (184), a mixture of C-3, C-4 epimers called epiconicol, and didehydroconicol (185) with a condensed aromatic ring system (Fig. 24).^213,215,217,218^ All compounds showed cytotoxic and weak anti-bacterial activity.

**Fig. 25:**
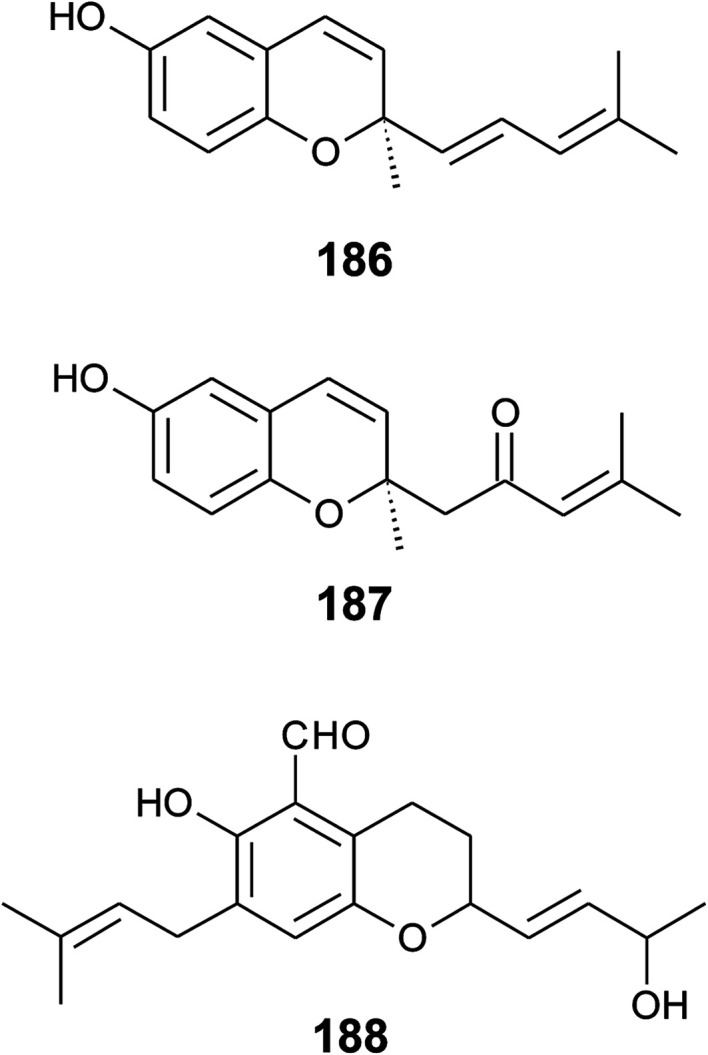
Structures of monoterpenes (186 to 188) from tunicates and algal derived endophytic fungi.

Two optically active cordiachromenes were isolated from the Australian tunicae *Aplidium solidum*, one with an additional 2′–3′ double bond (186), the other with a saturated side chain and a 2′-ketone group (187; Fig. 25).^219^

#### 5.2.4 Marine algal-derived endophytic fungi

Chaetopyranin (188) with a C-7 skeleton was isolated from the marine red algal-derived endophytic fungus *Chaetomium globosum* (Fig. 25).^220^ Biosynthetically, it may be generated from a meromonoterpene and loss of two methyl groups or – more likely – from a derivative of flavoglaucin, which is quite common in different fungi strains. The fungus was derived from the red alga *Polysiphonia urceolata*. Chaetopyranin was cytotoxic against human microvascular endothelial cells, hepatocellular carcinoma cells (SMMC-7721) and human lung epithelial cells (A549) with IC_50_ values of 15.4, 28.5 and 39.1 μM, respectively.

## 6. Hemiterpenes

### 6.1 Plants

**Fig. 26:**
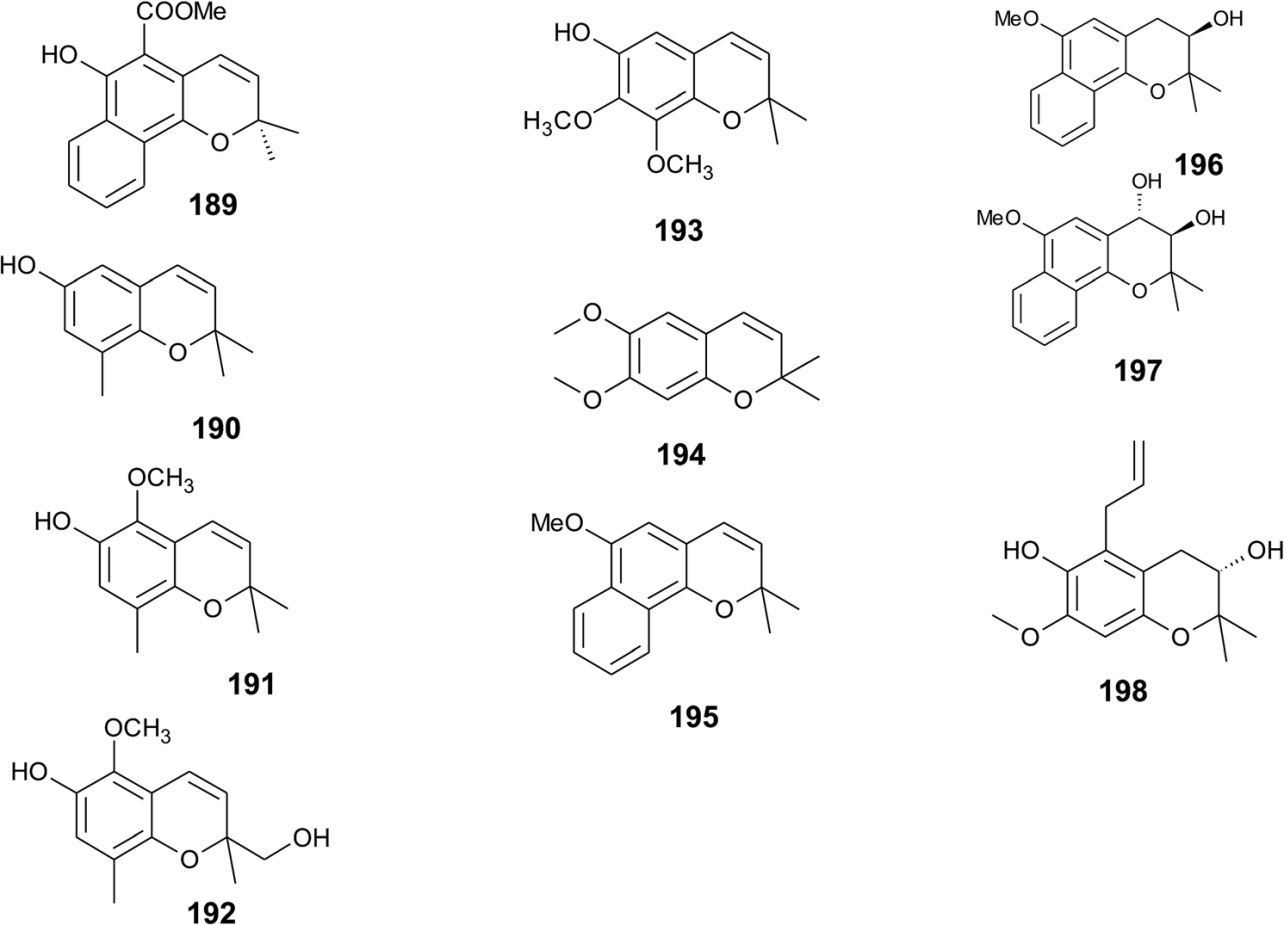
Structures of hemiterpenes (189 to 198) from plant species.

The following hemiterpenes exhibit interesting biological and pharmacological activities, among them mollugin (189) (methyl 2,2-dimethyl-6-hydroxy-2H-naphtho[1,2-*b*]pyran-5-carboxylat) from the Chinese medicinal plant *Rubia cordifolia*. Biogenetically, mollugin was formed by a cyclisation of a prenylated naphthoquinone and is not related to the biosynthetic pathway of tocopherols (Fig. 2). Mollugin has been first reported by Schildknecht *et al.* as a pigment from the rhizomes of *Galium mollugo*^221^ and was further investigated for pharmacological effects, such as anti-platelet aggregation activity and anti-viral activity against hepatitis B virus.^222^ Mollugin has been shown to induce apoptosis in different types of cancer cells. It exhibited IC_50_ values of 12.3 μM, 23 μM and 60.2 μM on human colon cancer cells (Col2),^223^ human Jurkat T-cells^224^ and murine NIH 3T3-L1 preadipocytes,^225^ respectively. In the presence of 10 μM mollugin, a human multidrug-resistant breast cancer cell line (MCF-7/adr) was more susceptible to doxorubicin (IC_50_ decreased from 60 to 7.5 μg ml^−1^).^226^ Several patents describe the use of mollugin for different applications (Fig. 26).^227^

Several low molecular weight chromenes, such as pterochromenes L1 (190), L2 (191) and L4 (192), were isolated from Taiwanese *Pteris lingipinna*^228^. 6-Hydroxyeupatoriochromene B (193) was obtained from *Ageratina riparia* (Asteraceae)^229^ and finally, one of the first insect anti-juvenile hormones, precocene 2 (194) (6,7-dimethoxy-2,2-dimethylchromene) was found in *Ageratum houstonianum* (Asteraceae).^230^ Lapachenol (195), a naphthalene derivative, was isolated from the heartwoods *Paratecoma peroba*, *Tabebuia chrysantha* and *Tabebuia heptaphylla*.^231,232^ 3-Hydroxy (196) and 3-,4-dihydroxy-chromanes (197) were isolated from a trunkwood extract of *Tabebuia heptaphylla*.^232^

Zhuang *et al.* isolated illihenryipyranol A (198) from roots of *Illicium henryi* in minor amounts.^233^

### 6.2 Fungi

Deadalin A (199), also called quercinol, was independently discovered by Morimura *et al.* and Gebhardt *et al.* from the mycelial culture broth of *Daedalea dickinsii* and *Daedalea quercina*, respectively.^234,235^

**Fig. 27:**
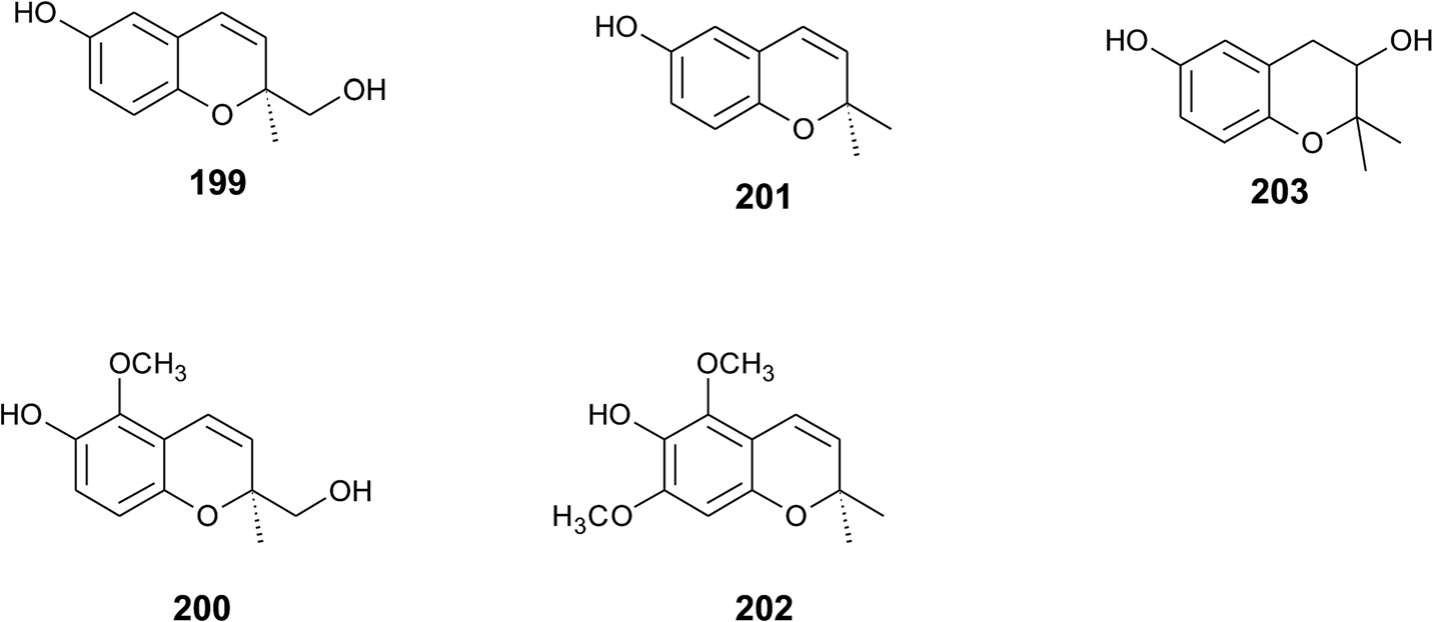
Structures of hemiterpenes (199 to 202) from plants and fungi.

Later, 5-methoxy-deadalin A (200), 6-methoxy-deadalin A (201), and 9-deoxy-deadalin A (202) were isolated from *Daedalea dickinsii* by Morimura and colleagues (Fig. 27).^236^ Deadalin A has been shown to have anti-tyrosinase activity (IC_50_ of 194 μM) and thus to inhibit melanin synthesis in a three-dimensional human skin model.

Tanaka *et al.* isolated a 3-hydroxy-chromane (203) from *Acremonium murorum*,^237^ a hyaline phialide.

All hemiterpenoid chromanols from fungi are derived from simple prenylated phenols and not related to the biosynthetic pathway of tocopherols.

## 7. Animal tocochromanol metabolism

For a complete overview of side chain-modified 6-hydroxy-chromanols, we present in the following animal and human vitamin E metabolites. In recent years, these metabolites have been intensively studied for anti-inflammatory and cytotoxic activity (see also sections below) and were discovered as novel regulatory and signaling molecules. Studies on vitamin E metabolism were summarized in several outstanding reviews;^17,238,239^ we therefore describe here only briefly the formation and activities of these metabolites.

**Fig. 28:**
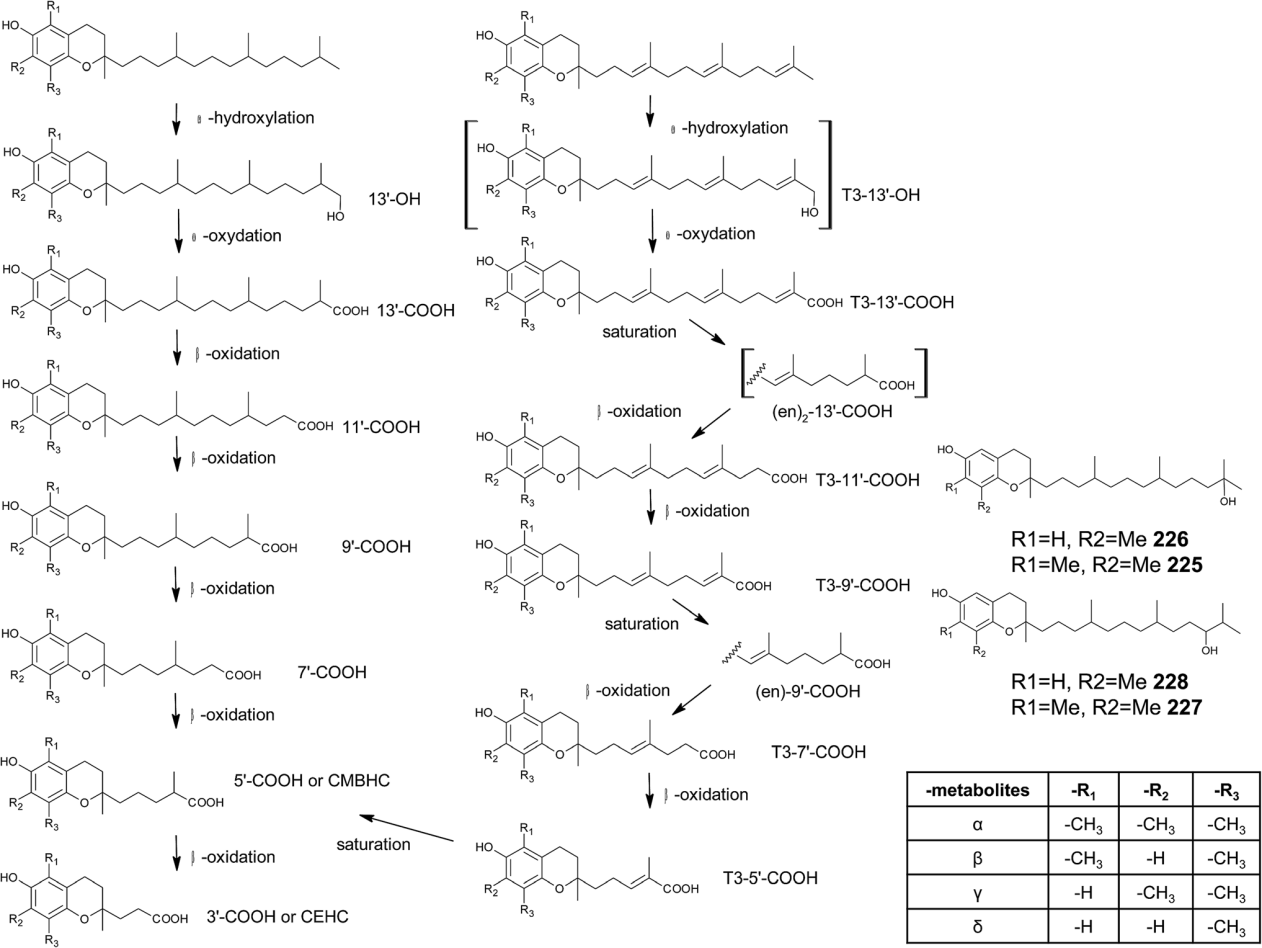
Mammalian metabolism of tocopherols and tocotrienols. α-TOH: α-tocopherol; α-tocotrienol: α-tocotrienol; 13′-OH: 13′-hydroxy-chromanol; 13′-COOH: 13′-carboxy-chromanol; CDMD(en)2HC: carboxy-dimethyl-decadienyl-hydroxy-chromanol; CDMOenHC: carboxy-dimethyl-octenyl-hydroxy-chromanol; CDMHenHC: carboxy-methyl-hexenyl-hydroxy-chromanol; CMBenHC: carboxy-methyl-butadienyl-hydroxy-chromanol; CDMOHC: carboxy-methyl-octyl-hydroxy-chromanol; CDMHHC: carboxy-methyl-hexyl-hydroxy-chromanol; CMBHC: carboxy-methyl-butyl-hydroxy-chromanol; CEHC: carboxy-ethyl-hydroxy-chromanols.

The hepatic metabolism of tocopherols follows the classical activation of branched chain hydrocarbons by cytochrome P_450_ enzymes (most likely CYP4F2) within the endoplasmic reticulum.^240^ ω-Hydroxylation of α-tocopherol forms α-13′-hydroxy-tocopherol (204) (13′-OH) with subsequent oxidation to α-13′-carboxy-tocopherol (205) (13′-COOH) by aldehyde dehydrogenase (Fig. 28). Both metabolites were detected in human plasma and show anti-inflammatory and cytotoxic activity in *in vitro*- and *in vivo* systems (see corresponding Section 8 and 9).^241,242^

Further degradation of the long-chain metabolites (LCM) occurs like that of methyl branched-chain fatty acids by β-oxidation, subsequently cutting out 2- and 3-carbon units, respectively. The β-oxidation takes place in the peroxisomes and results in 11′-COOH and 9′-COOH LCM.^240^ Mustacich *et al.* suggested that further degradation occurs within the mitochondrial matrix where intermediate-chain metabolites (7′-COOH and 5′-COOH) and short-chain metabolites 3′-COOH (or carboxy-ethyl-hydroxy-chromanol (CEHC)) were detected.^240^ CEHCs were the first metabolites identified in human and animal (rats and mice) studies, since they are secreted in urine.^243^ δ-9′-COOH (206), α-5′-COOH (also known as α-carboxy-methylbutyl-hydroxy-chromanol, α-CMBHC; 207), and α-3′-COOH (α-CEHC; 208) were investigated for their anti-inflammatory properties *in vitro*.^244,245^ Interestingly, the degradation of tocotrienols results in the formation of CEHC, suggesting a similar degradation mechanism as seen for tocopherols. Indeed, analogous catabolic steps were found for tocotrienol *in vitro* and *in vivo*^242,246^ (Fig. 28). Initial ω-hydroxylation followed by five cycles of β-oxidation follows in principle that of tocopherols; however, the double bonds between C-4′–C-5′, C-7′–C-8′ and C-11′–C-12′ undergo a saturation step, which is catalyzed by 2,4-dienoyl-CoA reductase and 3,2-enoyl-CoA isomerase (Fig. 28). The following metabolites of tocotrienol were identified by *in vitro* experiments in hepatic adenoma HepG2 cells and in mice feces, respectively:^242,246^ 13′-carboxy-trienols (α-, γ-, δ-tocotrienol-13′-COOH; 209, 31, 30) which are identical with the naturally occurring garcinoic acids, *e.g.* α-, γ-, and δ-garcinoic acid, carboxy-dimethyl-decadienyl-hydroxy-chromanol (α-, γ-, δ-CDMD(en)_2_HC or α-, γ-, δ-(en)_2_-11′-COOH, (210, 211, 212)), carboxy-dimethyl-octadienyl-hydroxy-chromanols (α-, γ-, δ-CDMO(en)_2_HC or α-, γ-, δ-(en)_2_-9′-COOH, (213, 214, 215)), as well as carboxy-dimethyl-octenyl-hydroxy-chromanols (α-, γ-, δ-CDMOenHC or α-, γ-, δ-(en)-9′-COOH, 216, 217, 218), carboxy-methyl-hexenyl-hydroxy-chromanol (α-, γ-, δ-CMHenHC or α-, γ-, δ-(en)-7′-COOH, (219, 220, 221)), and carboxy-methyl-butadienyl-hydroxy-chromanol (α-, γ-, δ-CMBenHC or α-, γ-, δ-(en)-5′-COOH (222, 223, 224),). Tocotrienols are metabolized with a higher rate than α-tocopherol; thus, depending on dietary tocotrienol intake, the plasma concentrations exceed that of α-tocopherol metabolites. Except for the 13′-carboxy-trienols (see next section), biological properties of tocotrienol metabolites are largely unknown.

All tocopherol and tocotrienol metabolites can occur in free form or as phase II conjugates, such as sulfates or glucuronides.^242,247^

Bardowell *et al.* investigated the role of the murine *Cyp4f14* gene, an orthologue of the human *CYP4F2* gene, in vitamin E metabolism in Cyp4f14-knockout mice and found two new metabolites, namely 12′-hydroxy-tocopherol (12′-OH: γ- and δ-12′-OH, (225 and 226)) and 11′-hydroxy-tocopherol (11′-OH: γ- and δ-11′-OH, (227 and 228)) in fecal pellets of mice fed a diet rich in γ-tocopherol.^248,249^ The metabolites derive from ω-1 and ω-2-hydroxylation and were excreted *via* bile into feces of mice and humans.

## 8. Anti-inflammatory activity of toco-chromanols and -chromenols

Many diseases, including atherosclerosis, diabetes or even cancer, are related to inflammatory processes. A decreased grade of inflammation could lead to a reduced risk for these diseases. In the past, human clinical trials with α-tocopherol as an anti-inflammatory agent revealed contradictory results.^17,239,250,251^ We here like to broaden the view to structurally related chromanols and chromenols and compare their anti-inflammatory *in vitro* activity.

The anti-inflammatory activities of tocopherols and tocotrienols from the human diet are well known and are compiled in Table 1.^239,245,252^ In general, the chromanols with saturated and unsaturated side chains showed good to moderate inhibitory activity depending on the anti-inflammatory marker measured and the *in vitro* system used.^253,254^ For example, Jiang *et al.* investigated the inhibition of COX-2 catalyzed PGE_2_ synthesis in IL-1β stimulated human lung epithelial A549 cells of a series of tocopherols and tocotrienols, respectively.^245^ The inhibitory activities reached from IC_50_ > 50 μM for α-tocopherol to IC_50_ = 1–3 μM for δ-tocopherol and γ-tocotrienol.

The LCM of tocopherols and tocotrienols with a terminal C-13′-carboxy and -hydroxyl group, respectively, were found in nanomolar concentration in human plasma and intensively studied as anti-inflammatory agents. The research on the LCM was promoted by the facile semi-syntheses from garcinoic acid that can be efficiently isolated from *Garcinia kola*.^38,39^

α-13′-Carboxy-tocopherol (205) (α-13′-COOH) inhibited the expression of iNOS by 100% at 5 μM and the formation of nitric oxide by 100% at 2.7 μM, respectively.^255,256^ A recent investigation on the anti-inflammatory activity of α-13′-COOH showed strong inhibition of recombinant 5-LOX and only moderate inhibition of COX-1, leukotriene (LT) C_4_ synthase, PGES-1 and epoxide hydrolase.^254^ Human recombinant COX-2 was not inhibited by α-13′-COOH at low concentrations.

The δ-tocopherol metabolite δ-13′-carboxy-tocopherol (229) (δ-13′-COOH) showed a slightly reduced activity, and inhibited iNOS protein synthesis by 56% at 5 μM and nitric oxide formation by 79% at 5 μM, respectively, in LPS-activated murine RAW264.7 macrophages. δ-13′-COOH further inhibited the LPS-induced upregulation of COX-2 expression in the same cells with IC_50_ values of 4–5 μM.^245,257^ 5-LOX activity was inhibited in the range from 0.5–2.0 μM, depending on the assay used.^253^

The 13′-Hydroxy-tocopherols of α-, and δ-tocopherol (α-13′-OH (204) and δ-13′-OH (231)) are synthetically available and have been therefore intensively studied. α-13′-OH reduced COX-2 expression (49%) and PGE_2_ synthesis (54%) and both alcohols inhibited iNOS expression by 53–60% at 10 μM in LPS-induced murine RAW264.7 macrophages.^241,256^ Both alcohols can be biochemically converted by mammalian cells to the corresponding acids, thus making it difficult to distinguish between the activity of 13′-OH and 13′-COOH (unpublished results).

As described in the section on human metabolism, the metabolic truncation of the LCM leads to several medium- and short-chain metabolites, such as 9′-carboxy-tocopherols (9′-COOH), CMBHC and CEHC, respectively. In general, the anti-inflammatory activities of the medium- and short-chain metabolites seem to decrease with the decreasing lengths of the side chains, resulting in higher IC_50_ values (Table 1).^245^

In summary, although the number of *in vitro* studies ist still limited and different markers of inflammation cannot be compared directly, we roughly estimate the anti-inflammatory activity of tocopherols, tocotrienols and their metabolites as follows: α-tocopherol < non-α-tocopherol ∼ tocotrienols ≪ 13′-OH ∼ 13′-COOH ≫ 9′-COOH > CMBHC ∼ CEHC. However, it must be kept in mind that the molecular modes of action of these molecules seem to be quite different. It is obvious that the impact of the metabolites depends on individual metabolism rates (pharmacokinetics) of the tocochromanols from the diet. Grebenstein *et al.* proposed that the affinity of vitamers towards the α-tocopherol transfer protein (α-TTP) may predict their degradation by cytochrome P_450_ enzymes.^258^ α-TTP has the strongest affinity for α-tocopherol with *K*_d_ of 25 nM and much higher *K*_d_ values for the other vitamin E forms, depending on their methylation pattern and side chain saturation.^239,259^ Accordingly, the catabolism of non-α-tocopherol vitamers into the corresponding LCM may occur much faster than that of α-tocopherol, thus generating more anti-inflammatory metabolites. As a result, α-tocopherol *per se* is less active than all other vitamers following the order: δ-tocopherol ∼ γ-tocotrienol > γ-tocopherol ≫ α-tocopherol.^239^

δ-Garcinoic acid (30) is the main constituent of several *Garcinia* species, which are known for their anti-inflammatory properties in African ethnomedicine.^37^ δ-Garcinoic acid was reported to inhibit COX-2 (IC_50_ = 10 μM) and, even stronger, 5-LOX with IC_50_ ranging from 0.04 to 1.0 μM.^257^ δ-Garcinoic acid down-regulated the LPS-induced expression of pro-inflammatory cytokines, such as TNF-α, IL-6, IL-1β, COX-2 and iNOS in macrophages and reduced production of nitric oxide (IC_50_ value of 1 μM).^255,260^ A direct comparison of several carboxy-tocotrienols (tocotrienol-13′-COOH metabolites) from plant origin as inhibitors of microsomal PGE_2_ synthase-1 revealed the following order of activity: γ-garcinoic acid (31) > β-garcinoic acid (232) > δ-garcinoic acid (30) > α-garcinoic acid (209); however the methylation pattern had only moderate impact.^44^

Structurally related forms of δ-garcinoic acid, such as δ-sargachromenol (51) with a 15′-COOH group and a chromene ring system, showed only moderate inhibitory activity on in LPS-stimulated production nitric oxide and PGE_2_ in murine RAW 264.7 macrophages (IC_50_ values of 82 μM and 30.2 μM, respectively);^129^ however, much higher activity was observed in BV-2 microglial cells (IC_50_ value for inhibition of nitric oxide production of 1.3–2.7 μM).^134,261^

As described above, 13′-OH metabolites have a similar anti-inflammatory potential than the corresponding 13′-COOH. Thus, natural products such as sargachromanols D (57), E (58) and G (60), respectively, with hydroxyl-groups at C-9′ and C-10′ are interesting intermediates. They all showed moderate inhibitory activity on nitric oxide production in LPS-stimulated murine RAW 264.7 cells (IC_50_ values of 15–40 μM).^79,83,84^

Cyclic meroditerpenes such as epitaondiol (79) and the chromarols A to D (113 to 116) exhibited anti-inflammatory activity *in vitro* and *in vivo* (Table 1).^99,154^ The four chromarols A to D inhibited 15-LOX with IC_50_ = 0.6(113), 4.0(114), 0.7(115) and 1.1 μM (116), respectively, but not 12-LOX. Epitaondiol was effective in a TPA-induced mouse ear edema study (IC_50_ = 20.7 μg per ear) and inhibited eicosanoid synthesis with an IC_50_ of 3.8 μM for thromboxane B_2_ (TXB_2_) and an IC_50_ of 30.1 μM for LTB_4_.

The cyclic sesquiterpenes capillobenzopyranol (172) only moderately inhibited nitric oxide production in LPS-stimulated macrophages by 37% at 10 μM.^202^ The monoterpene cordiachromene A (173) inhibited soybean 15-LOX with an IC_50_ of 0.82 μM and lipid peroxidation with an IC_50_ of 2 μM.^214^

Only moderate anti-inflammatory activity was observed for the hemiterpene quercinol (199), whereas it inhibited COX-2 expression with an IC_50_ of 0.63 μM.^235^

**Fig. 29:**
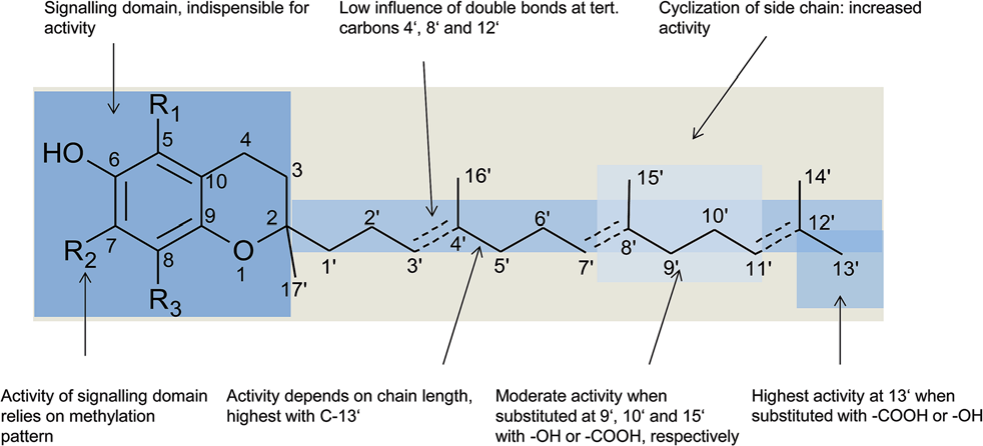
General domains modulating the anti-inflammatory activity of meroterpenoids.

In Fig. 29 we postulate the structural motives that are essential for the anti-inflammatory activity based on the structures and properties discussed above. The most effective compounds described are the diterpenes 13′-COOH, 13′-OH, garcinoic acid and δ-sargachromenol, respectively, with strong potential as anti-inflammatory drug candidates. A recent SAR study revealed that the effects of human LCM depend on the presence of the chromanol ring and modifications in the side chain and less on the substitution pattern at the aromatic ring.^256^ This study is in line with the observation of Silva *et al.* with δ-sargachromenol (51) and its precursor 1,4-benzoquinone sargaquinoic acid; the latter had less inhibitory activity towards LOX- and COX-enzymes.^52^ In addition to the natural compounds described above, the anti-inflammatory and anti-diabetic drug troglitazone exhibits a 6-hydroxy-chromane ring system. Troglitazone was used a PPAR-γ-receptor agonist but was withdrawn from the market since it caused hepatotoxicity.^262^ Obviously, anti-inflammatory activity is enhanced by the occurrence of a 6-hydroxy-chromane and -chromene moiety, respectively.

In conclusion, meroditerpenoids with a functional group (COOH, OH) at the side chain have much higher anti-inflammatory activity than the parent chromanols and chromenols, respectively.

## 9. Anti-proliferative and cytotoxic activity of chromanols and chromenols

Dietary tocopherols and tocotrienols have been extensively investigated for their cancer-preventive potential in several human intervention trials (reviewed in^263^ and^6^), but widely failed to prove beneficial effects.^264^ However, *in vitro* studies with tocopherols and tocotrienols in cell cultures and *in vivo* studies have shown pronounced anti-neoplastic and anti-carcinogenic effects.^265,266^

The susceptibility of the cell lines tested for anti-carcinogenic activity varied tremendously and makes thus it difficult to compare the compounds discussed in this section. For example, HepG2 liver cells exhibit greater resistance to drugs and toxins compared to other cells lines, since they actively express phase I and II enzymes. As a result, higher IC_50_ values are expected for drug resistant cell lines, such as HepG2.

Structure–activity relationship studies revealed that chemical modifications at C-6 of the aromatic ring (ethers or esters) magnified the cytotoxic potential of vitamin E compounds.^266,267^ In general, drug candidates that were ‘redox-silent’ at C-6, such as tocopherol-succinate, showed promising results in animal studies.^268^ Although most of the redox-silent compounds were chemically synthesized, distinct structure–activity relationships have been derived from these experiments.^266^ The studies revealed the importance of three major domains of the chromanols tested: first, the functional domain (I) that needs to be ‘redox silent’ to exert the cytotoxic properties. Second, the signaling domain (II) modified by the methylation pattern of the chromanol ring system. Third, the hydrophobic domain (III) that is mostly covered by saturated and unsaturated side chains.^267^ Reviewing the structural features of the molecules presented here, we further specify the domains that are relevant for cytotoxicity.

Tocopherols seem to have low or moderate anti-cancer activity in the different cell model systems.^267,269^ α-Tocopherol (3) in particular is well-tolerated by adenoma and cancer cells in supra-physiological concentrations (above 100 μM) (see Table 2).^269^ γ-Tocopherol (5) at 25 μM, however, had anti-proliferative activity on colon carcinoma (CaCo-2), androgen-sensitive (LNCaP) and androgen-resistant (PC-3) prostate, lung adenocarcinoma (A549), and osteosarcoma (SaOs-2) cells, but did not induce apoptosis in these cell lines.^269,270^ Accordingly, we postulate the following order of activity for tocopherols: γ-tocopherol (5) > δ-tocopherol (6) ≫ α-tocopherol (3).

Tocotrienols showed anti-proliferative and pro-apoptotic effects *in vitro* and *in vivo* and are in general more potent in the prevention of cancer than tocopherols.^271^ Several molecular targets were identified for γ- and δ-tocotrienols (16) and (17) (γ- and δ-tocotrienol), respectively (summarized by^272^). Induction of mitochondrial apoptosis, demonstrated by activation of caspase-3 and -9, along with modulation of apoptogenic genes such as Bcl-2, Bcl-xl and Bax, respectively, has been observed for most of the tocotrienols tested.

α-Tocotrienol (14) showed low to moderate pro-apoptotic activity against different breast cancer (MDA-MB-435, MDA-MB-231, and MCF7) and melanoma (B16) cells, respectively (Table 2).^25,273,274^ In contrast, γ- (16) and δ-tocotrienol (17) induced apoptosis at low micromolar concentrations in most of the cell lines tested.^25,267,275,276^ Sargaol (95) (3-4-dehydro-δ-tocotrienol), has been tested with moderate activity in human gastric epithelial cells, human fibroblasts and murine lymphocytic leukemia P338 cells.^103,120^ Interestingly, fascioquinol F (109) (3-4-dehydro-desmethyl-tocotrienol) showed no inhibitory activity against human gastric adenoma (AGS) and human neuroblastoma (SH-SY5Y) cells, respectively.^151^ In contrast, desmethyl- and didesmethyl-tocotrienol (18) and (19) strongly induced apoptosis in B16 melanoma cells (IC_50_ values of about 1 μM).^24,25^ In conclusion, the activity order is determined by the methylation pattern of the chromanol ring: desmethyl-tocotrienol (18) ∼ didesmethyl-tocotrienol (19) > γ-tocotrienol (16) ∼ δ-tocotrienol (17) ∼ 3-4-dehydro-δ-tocotrienol (95) ≫ α-tocotrienol (14).

Only recently, tocopherol- and tocotrienol-metabolites were investigated for their anti-carcinogenic activity. 13′-Carboxylic acids, including garcinoic acid, induced apoptosis in the lower micromolar range with slight differences depending on their methylation pattern and double bonds in the side chain, respectively. The tocopherol metabolites 13′-carboxy-α-tocopherol (205) and 13′-carboxy-δ-tocopherol (229) induced caspase-3-dependent apoptosis in human HepG2 liver cells (IC_50_ values of 13.5 μM and 6.5 μM, respectively).^39^ Similar activities were observed in human THP-1 macrophages, glioma C6, colon carcinoma HCT-116 and colon adenocarcinoma HT-29 cells (Table 2).^38,257,277^ The natural product and tocotrienol metabolite garcinoic acid (30) showed similar activities. δ-Sargachromenol (51) and fallachromenoic acid (105) were both active in the lower micromolar range.^126,133^ Thus, the shift of the carboxylic group at C-15′ does not affect the pro-apoptotic activity.

The introduction of hydroxyl group(s) within the side chain is associated with pro-apoptotic effects. Crassumtocopherols A (134) and B (135) (C-8′–C-11′–C-12′-triols) showed strong inhibitory activity towards murine P338 leukemia and human colon adenocarcinoma HT-29 cells (IC_50_ values of 5.2–7.5 μM).^169^ Somewhat lower activities were found for diols such as δ- and γ-amplexichromanols (35) and (36) (C-13′–C-14′-diols), litchtocotrienol A (41) (C-11′–C-12′-diol), and sargachromanol E (58) (C-9′–C-10′-diol) (Table 2).^43,49,82^ Sargatriol (98) (C-5′–C-6′-diol), as well as sargadiol-I (96) (C-6′–OH) and -II (97) (C-8′–OH) are weak inhibitors in P338 leukemia cells with IC_50_ values between 30 and 40 μM.^118,120^ All compounds with mono-hydroxy-substituted side chains showed low to no cytotoxic activity. Based on the hydroxylation pattern of the side chain, we estimate the following activity order: C-8′–C-11′–C-12′-triol > C-13′–C-14′-diol ∼ C-11′–C-12′-diol ∼ C-9′–C-10′-diol > C-8′–OH ∼ C-5′–C-6′-diol ∼ C-6′–OH ≫ C-13′–OH.

Cyclizations of the tocotrienol side chain lead to epitaondiols, strongylophorines and bifurcarenone-derived chromanols. All compounds tested showed moderate to weak anti-cancerogenic activities (Table 2)

Unfortunately, only few data exist for the anti-cancer activities of sesquiterpenes. Paniceins A2 (160) and F2 (161) both inhibited growth of P330, lung adenocarcinoma A549 cells, uveal melanoma MEL20 cells, and colon adenocarcinoma HT-29 cells with IC_50_ values of around 15 μM (ref. 195) and riccardiphenol C (146) was not active.^177^

Monoterpenes and hemiterpenes both demonstrated medium to low inhibitory activity towards cancer cells (Table 2).

**Fig. 30:**
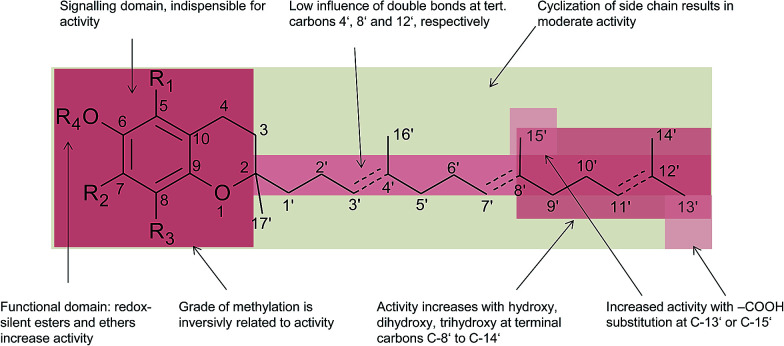
General structural motives important for the anti-cancer activities of meroterpenoids.

In conclusion, meroditerpenoids exhibited the strongest inhibitory activity towards cancer cells among all meroterpenoids described, especially when a carboxy or more than one hydroxyl group is present at the terminal end of the side chain (Fig. 30).

## 10. Discussion

**Fig. 31:**
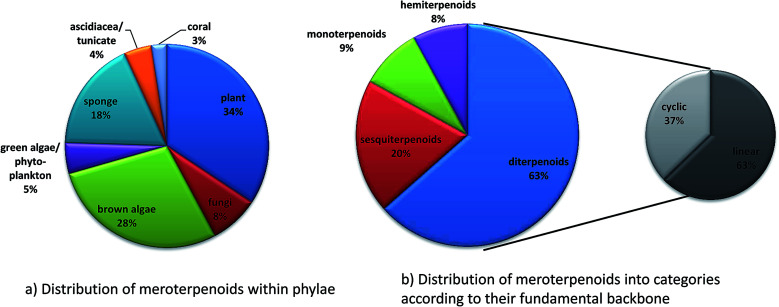
Statistical distribution of meroterpenoids.

This review describes more than 230 6-hydroxy-chromanols and -chromenols, respectively that were found in terrestrial and marine organisms. Fig. 31a highlights the distribution of meroterpenes within different phylae. Marine organisms, led by brown algae (Phaeophyceae), cover two thirds of the molecules presented in this review, followed by plants and fungi. Interestingly, sponges (porifera) produce 18% of the natural products presented here, mainly cyclic di- and sesqui-terpenes.

Meroditerpenes represent almost two thirds of all compounds discussed and are divided into 63% with linear and 37% with cyclic side chains, respectively (Fig. 31b). The occurrence of sesquiterpenes was dominant in sponges, whereas hemiterpenes were only found in plants and fungi.

During the course of this compilation, the question arose whether or not the stereo-controlled cyclization of toluquinols to a chromane or chromene ring with *R*-configuration at C-2 occurs exclusively in terrestrial species. The evidence for this process in plants is well documented and the isolation of several cyclases substantiates the biosynthetic step. Marine-derived meroterpenes were often isolated as mixtures of stereoisomers at C-2 and several authors debated the isolation of chrom(e)anols as artefacts of the work-up procedures or as non-enzymatic reaction products within the organism. In addition, the monocyclic 1,4-benzoquinone precursors were isolated in most cases with high yields, whereas toluquinols in plant species occur only in trace amounts and were rarely described. From the 49 diterpenes isolated from plants, 46 (94%) were described with *R*-configuration. A statistical analysis of chromanols and chromenols from marine species revealed that 73% of chromanols were isolated as *R*-enantiomers, whereas only 26% of all chromenols show optical activity with *R*-configuration. In conclusion, we postulate that marine organisms most likely produce chromanols *via* enzyme-catalyzed cyclization, whereas chromenols may mostly originate from non-enzymatically cyclization or as an artefact during sample work-up.

The structural variability of the compounds described in this review is remarkable. Side chain modifications by oxidation and/or cyclization occur widely, especially in marine organisms. Cytochrome P_450_ enzymes are most likely responsible for the initial oxidation to epoxy-, hydroxy- and carboxy-derivatives, respectively, although the corresponding enzymes were studied only in animal vitamin E metabolism and are not fully understood yet.^248,249^

Cyclization of the prenylated side-chain occurs *via* different pathways. The first pathway begins with an acid-catalyzed cyclization cascade between C-2–C-7, C-6–C-11 and C-10–C-15 of the sesquiterpenes and diterpene backbone, respectively, that leads to di- or tricyclic 1,4-hydroquinones. This is followed by a second acid-catalyzed formation of the chromane ring as described by Kurata *et al* (Fig. 20).^186^ Several examples for this cyclization, such as chromazoranol (154) or strongylophorines (117–130), are described above.

The second cyclization pathway occurs *via* an epoxidation of the terminal double bond, followed by an acid-catalyzed cyclization cascade with a final cyclization of the chromane ring, as first described by Etse *et al.*^61,92,93^ (see also Fig. 7 and 10). It remains unclear, if these mechanisms occur simultaneously or sequentially. Walsurol (50), cyclolitchtocotrienol A (49) or the taondiols (76–80) are examples of the second pathway.

A third cyclization pathway occurs *via* the formation of the 1,4-hydroquinone precursor bifurcarenone (81) by an acid-catalyzed anti-Markovnikoff cyclization between C-7 and C-11.^278^ Subsequent cyclization reactions lead to cystoketal chromane (89), mediterraneols (82–84) and cystoseirols (86, 87).

Only three meroterpenes with a cyclic side chain have been described in plants, namely walsurol (50), cyclolitchtocotrienol A (49) (Fig. 7) and riccardiphenol C (146) (Fig. 19).

With some exceptions, all higher plants produce side chain-saturated tocopherols with the typical methylation pattern α-, β-, γ-, and δ-, respectively. Next, several algae have the ability to produce tocopherols, although in low yields. 8-Methyl- or desmethyl-tocotrienol moieties were found in most of the structures described from marine organisms. Only three tocopherol-derivatives with a full methylation pattern (α-) were identified in marine organisms, namely marine-derived tocopherol (25) from phytoplankton, α-tocoxylenoxy (108) from the green alga *Caulerpa racemosa* and chrassumtocopherol from the soft coral *Lobophytum crissum*.

The primary biological function of the side chain modifications remains unclear. On the one hand, cytotoxicity, algicidal and anti-macroalgal activity was found for several metabolites. On the other hand, the settling of sea urchins and perna eggs was induced by several compounds. Thus, side chain-modified metabolites are presumably used as chemical protectants or as signalling molecules for intercellular communication or both.

Recent advances in the research on human vitamin E metabolites led us to a comprehensive search for chromanol- and chromenol-structures with anti-inflammatory and cytotoxic properties (see Tables 1 and 2). The number of structurally related compounds exceeded our expectations. We therefore merged the available information on over 30 compounds and identified structural motives that correspond to high anti-inflammatory activities (Table 1). Most of the compounds described here affected arachidonic acid metabolism and also the synthesis of pro-inflammatory cytokines. Inhibition of COX-1 and COX-2 expression, respectively, reduced prostaglandin metabolite formation and inhibition of 5- and/or 12-LOX blocked leukotriene synthesis. Further studies will have to reveal if meroterpenoids have the potential to be developed into anti-inflammatory drug candidates.

Cytotoxicity data of approximately 50 compounds were collected (Table 2). Like the anti-inflammatory activities of meroterpenoids, diterpenes showed the strongest activity, led by side chain-modified chromanols. Anti-proliferative and cytotoxic properties were modulated by the presence of hydroxyl and carboxy groups. Activation of caspases-3 and -9, respectively, suggested that most of these compounds induce a mitochondrial death pathway.

Rangasany *et al.* evaluated the drug-likeness of several natural products isolated from algae and found δ-sargachromenol (51) and epitaondiol (79) as good fits to Lipinski's ‘Rule of Five’. This rule estimates the potential of a drug candidate based on physio-chemical properties, such as molecular weight, number of hydrogen bond acceptors and donors, and distribution coefficient (log *P*).^73,141^ We screened a series of compounds described in this review (ESI Table 1†) and found many with good predicted oral bioavailability, based on these calculations which were conducted *via* Molinspiration WebME editor version 1.16 (http://www.molinspiration.com).

We and others tested several vitamin E metabolites for their biological activity *in vitro* and *in vivo* and found them to have anti-bacterial, anti-viral, anti-inflammatory and cytotoxic properties (Tables 1 to 3). In general, any modification of the prenyl side chain increased their biological activity.

In this review, we thoroughly described the class of 6-hydroxy-chromanols and -chromenols within living nature and summarize their biological properties, in particular their anti-inflammatory and anti-carcinogenic potential. Based on the presented evidence, we conclude that the presence of a hydroxyl or carboxy group in the side chain enhances the anti-inflammatory activity of natural chromanols and chromenols. With respect to anti-proliferative and anti-cancer activities, we conclude that, among all meroterpenoids described, meroditerpenoids have the strongest inhibitory activity towards cancer cells, in particular when, again, bearing a carboxy or more than one hydroxyl group at the terminal end of the side chain. We therefore propose that the presence of a terminal hydroxyl or carboxy group in the side chain of the long-chain vitamin E metabolites warrants further investigation and might help us to unravel the as yet unknown essential biological function(s) and modes of action of vitamin E in animals.

## Conflicts of interest

There are no conflicts to declare.

## Supplementary Material

RA-008-C7RA11819H-s001
