# Supplementary material for: Natural 6-hydroxy-chromanols and -chromenols: structural diversity, biosynthetic pathways and health implications
Source: RSC Adv. 2018 Jan 26;8(9):4803–41. doi: 10.1039/c7ra11819h (PMC9078042; doi:10.1039/c7ra11819h)
Supplement: RA-008-C7RA11819H-s001 [file RA-008-C7RA11819H-s001.pdf]

Electronic Supplementary Material (ESI) for RSC Advances.
This journal is © The Royal Society of Chemistry 2018

Supplementary Table 1: Physio-chemical properties of anti-inflammatory chromenols and chromanols.[a] Thresholds proposed by "Lipinski's Rule of Five" are given in brackets.

**Compound**	**Molecular weight [<500 Da]**	**Octanol-water partition coefficient [<5 log *P*]**	**Number of atoms [20-70]**	**Number of hydrogen bond acceptors (O and N atoms) [<10]**	**Number of hydrogen bond donors (N-H and O-H bonds) [<5]**	**Polar surface area [≤140 Ǻ2]**	**Number of rotable bonds [<10]**	**Number of violations**	**Lipinski's Rule[b]**
δ-Sargachromenol (**51**)	424.58	7.44	31	4	2	66.76	10	1	Y
Epitaondiol (**79**)	412.61	6.36	30	3	2	49.69	0	1	Y
δ-Garcionic acid (**30**)	426.6	7.59	31	4	2	66.76	10	1	Y
13'-α-COOH (**205**)	460.7	8.37	33	4	2	66.76	13	2	Y
13'-α-OH (**204**)	446.7	8.45	32	3	2	49.69	13	2	Y
13'-δ-OH (**231**)	418.66	7.56	30	3	2	49.69	13	2	Y
13'-δ-COOH (**229**)	432.64	7.43	31	4	2	66.76	13	2	Y

Sargachromanol D/E (**57/58**)	428.6	6.89	31	4	3	69.92	9	1	Y
Sargachromanol G (**60**)	426.6	6.7	31	4	2	66.76	9	1	Y
α-CMBHC (**207**)	320.4	4.86	23	4	2	66.76	5	0	Y

[a] Values were calculated by Molinspiration WebME editor version 1.16 (http://www.molinspiration.com).

[b] Y = passed the rule (max. 1 violation)
